# In-Situ Formation of High-Performance β‑NiOOH OER Electrocatalysts Using Boron and Phosphorus-Enriched Ni Core–Shell Nanoparticles

**DOI:** 10.1021/acsami.4c22116

**Published:** 2025-05-16

**Authors:** Patrick Guggenberger, Prathamesh Patil, Bernhard Fickl, Christian M. Pichler, Bernhard C. Bayer, Martin Stockhausen, Thilo Hofmann, Guenter Fafilek, Freddy Kleitz

**Affiliations:** † Department of Functional Materials and Catalysis, Faculty of Chemistry, 27258 University of Vienna, Währinger Straße 42, 1090 Vienna, Austria; ‡ Vienna Doctoral School in Chemistry (DoSChem), University of Vienna, Währinger Straße 42, 1090 Vienna, Austria; § CEST Centre of Electrochemical and Surface Technology, Viktor Kaplan-Straße 2, 2700 Wiener Neustadt, Austria; ∥ Institute of Applied Physics, Technische Universität Wien, Wiedner Hauptstraße 8-10, 1040 Vienna, Austria; ⊥ Institute of Materials Chemistry, Technische Universität Wien, Getreidemarkt 9, 1060 Vienna, Austria; # Department for Environmental Geosciences, Centre for Microbiology and Environmental Systems Science, 27258 University of Vienna, Josef-Holaubek-Platz 2, 1090 Vienna, Austria; ∇ Institute of Chemical Technologies and Analytics, Technische Universität Wien, Getreidemarkt 9, 1040 Vienna, Austria

**Keywords:** water electrolysis, oxygen evolution reaction (OER), nickel-based electrocatalysts, electrochemical activation, core−shell nanoparticles

## Abstract

Electrocatalytic water splitting is key to achieving UN Sustainable Development Goal 7, clean energy. However, electrocatalysts with increased activity and reasonable costs are needed. Ni–B, Ni–P, and Ni–B–P-based systems have recently been proposed as particularly promising candidates, but lacked either an active surface or sufficiently high B and P concentrations, which hindered their catalytic performance. Therefore, we developed a tailored synthesis of Ni–B–P electrocatalysts. The resulting core–shell nanoparticles featured a highly porous borate-phosphate shell and a metallic core. This design provided an abundance of active sites for the oxygen evolution reaction (OER) while ensuring high electrical conductivity. Furthermore, screening of the annealing temperature was performed, and significant changes in surface chemistry were observed, as revealed by X-ray photoelectron (XPS) and low-energy ion scattering (LEIS) spectroscopy. Comprehensive cyclic voltammetry (CV) and operando electrochemical impedance spectroscopy (EIS) measurements revealed that leaching of P and B facilitated the formation of β-NiOOH, a compound recognized for its highly active sites in the OER, leading to excellent performance. Our results present a facile and scalable chemical reduction procedure to obtain tailored mesoporous Ni–B–P core–shell nanoparticles, and we believe that their pronounced activation for the OER can inspire the development of in situ-activated electrocatalysts.

## Graphical abstract

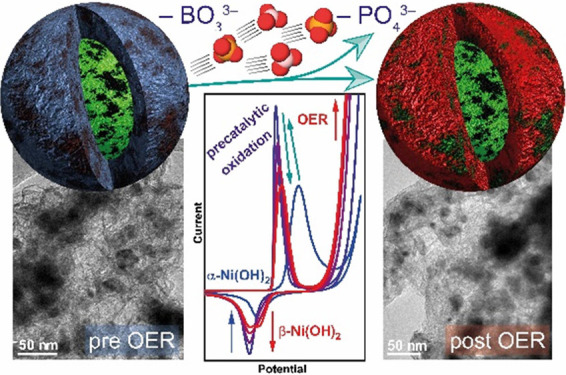

## 1 Introduction

To counteract the imminent climate crisis, a drastic cut in global greenhouse gas emissions from energy production and transportation is urgently needed, as reflected in the United Nations Sustainable Development Goal 7. Green hydrogen, generated from renewable-energy-driven electrocatalytic water splitting, is a promising sustainable alternative to fossil fuels, particularly where high energy density is needed. However, the reliance on expensive and scarce noble metal catalysts like Pt, IrO_2_, and RuO_2_ for efficient catalysis, combined with their limited energy efficiency due to large overpotentials for overall water splitting, hampers the economic competitiveness of green hydrogen. Among the nonprecious metals, nickel, as an abundant and relatively cheap element, has proven to be a sufficiently active electrocatalyst and is currently employed in commercial alkaline electrolyzers, although further improvement in activity and stability is desirable.^1^ Heteroatom doping (e.g., with B, P, S, ...) is a versatile method for improving the electrocatalysts' performance, enabling modulation of the atomic arrangements and electronic states to optimize the adsorption energies of reactants, improve the selectivity, and enhance the electrical conductivity, stability, and corrosion resistance.^2–7^ Lately, several reports demonstrated high water-splitting activities for Ni–B^8–13^ and Ni–P^14–17^ -based electrocatalysts. Furthermore, it was shown that the combination of Ni–B–P can greatly improve the materials’ performance for the hydrogen evolution reaction (HER) and oxygen evolution reaction (OER).^18–21^ While the hydrothermal synthesis methods reported by Zhao et al. and Ma et al. relied on subsequent Ni oxide/hydroxide intermediate phosphidation and boronation steps, Habib et al. developed a hydrothermal synthesis approach to directly grow Ni–B–P on the surface of Ni-foam.^18–21^ While these Ni–B–P catalysts exhibited remarkable performances, they lacked distinct mesoporosity; hence, a synthesis approach leading to high surface area nanostructured materials might lead to even better electrocatalysts due to facilitated diffusion of reactants and an increased number of accessible catalytic sites. Further, the preparation of nonsupported catalysts allows us to more thoroughly scrutinize the intrinsic OER activity using a rotating disc electrode (RDE). A simple aqueous solution precipitation procedure was developed to synthesize mesoporous Ni–B–P nanoparticles. Comprehensive bulk and surface chemical analysis was conducted, which revealed the formation of a core–shell nanoparticle structure with a metallic core and a phosphate/borate shell. We further observed significant activation of the catalyst during the initial cyclic voltammetry (CV) sweeps, which eventually led to excellent OER performance and could be linked to boron and phosphorus content, as these facilitated the formation of the highly active β-NiOOH. We further conducted post-OER X-ray photoelectron spectroscopy (XPS) and transmission electron microscopy (TEM) imaging to determine the structural and chemical stability of these new electrocatalysts.

## 2 Experimental Section

### 2.1 Materials

Nickel nitrate hexahydrate (Ni­(NO_3_)_2_·6H_2_O, 98%, Thermo Fisher Scientific, Germany), sodium borohydride (NaBH_4_, 98%, Alfa Aesar), sodium hypophosphide (NaH_2_PO_2_·*x*H_2_O, 98%, Honeywell Fluka, Germany), ethanol (96%, Brenntag, Austria), multielement standard solution 4 (for ICP, TraceCERT, Supelco, VWR Chemicals, Germany), nitric acid (HNO_3_ 65%, 3-fold subboiled, provided in analytically pure quality, Merck, Germany) and (HNO_3_ Trace SELECT, ≥69%, Honeywell Fluka, Germany), Nafion-117, (around 5% in a mixture of water and lower aliphatic alcohols, Sigma-Aldrich, Germany), KOH pellets (85%, VWR Chemicals, Germany), 2-propanol (Sigma-Aldrich, Germany), Al_2_O_3_ suspension (1 and 0.05 μm, MicroPolish suspension, Buehler Ltd., Germany) were used as purchased.

### 2.2 Molar Mass of NaH_2_PO_2_·*x*H_2_O

Due to the unspecified amount of crystal water in the NaH_2_PO_2_·*x*H_2_O precursor, thermogravimetric analysis (TGA) was performed to determine it. At 200 °C, the crystal water should be completely removed while no hypophosphite decomposition is expected.^22^ The measured weight loss of 14.09%, as depicted in Figure S1a, accounts for 9.93 mg (0.551 mmol) of water and the residual mass of 60.54 mg to 0.688 mmol of dehydrated NaH_2_PO_2_. Hence, the formula can be written as NaH_2_PO_2_·0.8 H_2_O, and the actual molecular mass is 100.8 g mol^–1^.

### 2.3 Synthesis of Ni–B–P Samples

The Ni–B–P sample was prepared by dropping (1 drop every two seconds) 75 mL of NaBH_4_ solution (1.5 g, 39.7 mmol) into 925 mL of a stirred solution of Ni­(NO_3_)_2_·6 H_2_O (4.361 g, 15.0 mmol) and NaH_2_PO_2_·0.8 H_2_O (4.361 g, 43.3 mmol) at room temperature (25 °C). The formation of a black precipitate and gas bubbles originating from the reduction of Ni^2+^ ions and the formation of hydrogen gas were directly visible. After the addition of the NaBH_4_ solution was completed, the suspension was stirred for another two h to ensure complete reaction of the reactants. The black powder was isolated by centrifugation, washed twice with deionized water and once with ethanol, and dried overnight at 60 °C in a convection oven to obtain the black ‘as-made’ (labeled AM) sample. The sample was split in equal fractions and annealed in a nitrogen atmosphere for 1 h at 150, 200, 250, 300, and 350 °C in a tube furnace (2 °C min^–1^ heating rate, 30 mL min^–1^ N_2_) to obtain the Ni–B–P_XXX samples, with XXX being the annealing temperature.

### 2.4 Synthesis of Ni–B Samples

The Ni–B sample was prepared under identical conditions but without the use of NaH_2_PO_2._ The final samples are labeled following the previous samples with, e.g., Ni–B_AM (as-made) and Ni–B_300 (for the sample annealed at 300 °C).

### 2.5 Synthesis of Ni–B–P-2 Samples

The Ni–B–P-2 sample was prepared under identical conditions but by doubling the amount of NaH_2_PO_2_·0.8 H_2_O (8.722 g, 86.5 mmol) in the initial solution. Again, the samples were labeled in the Ni–B–P-2_XXX format according to their respective annealing.

### 2.6 Material Characterization

Thermogravimetric analysis coupled with differential thermal analysis (TGA/DTA) was performed with a Netzsch STA 449 F3 Jupiter instrument (Selb, Germany) using alumina crucibles. To determine the amount of water in the NaH_2_PO_2_·*x*H_2_O precursor. The water removal was quantified in N_2_ atmosphere for a temperature range between room temperature and 200 °C, a heating rate of 10 °C min^–1^, followed by an isothermal step at 200 °C for 20 min to ensure complete water removal. The thermal behavior of Ni–B_AM, Ni–B–P_AM, and Ni–B–P-2_AM was screened in a nitrogen atmosphere (20 mL min^–1^) in a temperature range from 35 to 600 °C with a heating rate of 10 °C min^–1^.

An Anton Paar QuantaTech Inc. iQ3 instrument (Boynton Beach, FL, USA) was used to record N_2_ physisorption measurements at −196 °C. Prior to the measurements, the samples were dried at 80 °C overnight under vacuum. For data evaluation, we used ASiQWin 5.2 software, which was provided by the manufacturer. The Brunauer–Emmett–Teller specific surface area (BET SSA) was obtained by applying the BET equation to the measurement points in the relative pressure range 0.10–0.25 *P*/*P*_0_. The nonlocal density functional theory (NLDFT) was applied to determine the NLDFT SSA, NLDFT pore size, and NLDFT pore volume. Due to the cavitation-induced nitrogen desorption at 0.45 *P*/*P*_0_ for the Ni–B and Ni–B–P samples, we used the metastable adsorption branch for the NLDFT analysis to avoid artifacts in the pore size distribution (PSD).^23^ For the Ni–B–P-2 samples, the PSD was evaluated using both the adsorption and desorption branches.

Powder X-ray Diffraction (PXRD) measurements were conducted on a PANalytical EMPYREAN diffractometer in Bragg–Brentano HD reflection geometry with a PIXcel^3D^ detector (Malvern PANalytical, United Kingdom). The Cu K_α1+2_ X-rays were generated at a potential of 45 kV and a tube current of 40 mA. The diffractograms were recorded in continuous mode in the range 10–90° 2θ, using a step size of 0.013° and a time per step of 200 s. The phase fitting and Rietveld refinement were performed using the HighScore Plus software (version 4.7, Malvern PANalytical, United Kingdom).

X-ray fluorescence spectroscopy (XRF) was performed on an Epsilon 1 XRF analyzer (Malvern PANalytical, United Kingdom) using the Omnian measurement program; 200 mg of the respective sample was weighed into Teflon holders with inserted PP film (6 μm, Chemplex Industries, FL, USA). The quantification was performed using Epsilon 3 Software (Malvern PANalytical, United Kingdom).

Inductively coupled plasma–optical emission spectrometry (ICP-OES) was used to analyze the chemical composition of the Ni–B, Ni–B–P, and Ni–B–P-2 samples and the electrolyte during the chronopotentiometry test. The ICP-OES 5110 (Agilent, CA, USA) was operated at an RF power of 1.2 kW and an axial viewing mode. The nebulizer flow was set at 0.65 L min^–1^, and the plasma flow was set at 12 L min^–1^. For the calibration, the multielement standard solution 4 (for ICP, TraceCERT, Supelco, VWR Chemicals, Germany) was used, and the dilutions were performed with a 2 wt % HNO_3_ solution, which was prepared by diluting concentrated HNO_3_ (3-fold subboiled, provided in analytically pure quality, Merck, Germany) with ultrapure water (18.2 MΩ·cm). Five mg of the samples were digested in 1.33 mL of freshly prepared aqua regia (3:1 HCl: HNO_3_) overnight. An aliquot of the solution obtained was diluted with 2 wt % HNO_3_ (TraceSELECT, ≥ 69%, Honeywell Fluka) to a dilution of 1:200.

The Raman spectra were recorded on a WITec alpha 300A equipped with a 50x objective, a grating of 1200 cm^–1^, and a 531 nm laser with a beam power of 1.5 mW. Twenty measurements of 10 s acquisition time each were accumulated in one final spectrum for each sample.

A Zeiss Supra 55 VP scanning electron microscope (SEM) was employed to visualize the porosity and homogeneity of the sample. Elemental mapping was performed using the built-in energy dispersive X-ray spectroscopy (EDX) detector (Oxford Instruments, United Kingdom).

Transmission electron microscopy (TEM) was conducted on a FEI TECNAI F20 X-FEG instrument for bright-field TEM and on a scanning TEM (STEM, Gatan DigiSTEM II HAADF) for EDX mapping (EDAX-AMETEK Apollo XLTW SDD) at 200 kV electron acceleration voltage. The contrast and brightness of the STEM-EDX mapping images were increased *post hoc* to improve visibility.

X-ray photoelectron spectroscopy (XPS) measurements were conducted with a Versaprobe III (Physical Electronics, Germany) for all Ni–B–P samples, Ni–B_300, and Ni–B–P-2_300 using monochromatic Al Kα (1486.7 eV) radiation. Additionally, a Ni foil (NF) coated with Ni–B–P 300 catalyst ink was measured pre- and post-OER measurement. For all samples, a survey spectrum and high resolution (HR) B 1s, O 1s, P 2p, Ni 2p, and Ni L_3_M_45_M_45_ spectra were recorded. Additionally, an argon gas cluster ion beam was used to clean the surface of the Ni–B–P 300@NF samples. The peak deconvolution was performed using the Thermo Advantage 5.9922 software (Thermo Fisher Scientific, MA, USA).

The OER benchmark in 1 M KOH was performed for all electrocatalysts by using a PGSTAT302N Autolab (Metrohm, Switzerland) potentiostat. A Metrohm glassy carbon rotating disc electrode (RDE) with a geometric surface area of 0.196 cm^2^ was employed as the working electrode. A graphite rod was used as the counterelectrode and a HydroFlex reversible hydrogen electrode (RHE, Gaskatel, Germany) as the reference electrode. The temperature of the 200 mL three-electrode quartz glass cell was maintained at 25 °C using a thermostat that was connected to the cell’s water jacket. The 1 M KOH electrolyte was prepared by dissolving the pellets (85%, VWR Chemicals, Germany) in ultrapure water (18.2 MΩ·cm). The electrolyte was tested for Fe impurities and dissolved catalyst using ICP-OES before and during the OER measurements (dilution 1:200), and the Fe, B, P, and Ni levels were below the detection limit of the instrument. ICP-MS of the plain 1 M KOH electrolyte revealed Fe concentrations of 69.4 ppb. Prior to the measurements, the electrolyte was purged with N_2_ for 30 min to remove dissolved gas species. Gentle N_2_ bubbling was maintained throughout the measurements. The RDE was polished to a mirror finish using a wet polishing cloth with Al_2_O_3_ suspension (1 and 0.05 μm, Buehler Ltd.) and cleaned using ultrasonication in ultrapure water and a final rinse with 2-propanol. 4.8 mg of the catalyst was suspended for 30 min in 250 μL 2-propanol, 750 μL ultrapure water, and 50 μL Nafion-117 solution using an ultrasonic bath to yield a homogeneous black catalyst ink. 5.25 μL of freshly prepared ink was dropped on the polished RDE using an Eppendorf pipet to yield a catalyst loading of 0.12 mg cm^–2^. The ink was dried under light irradiation.

The measurements were conducted in triplicate to ensure reproducibility. The RDE was rotated at 2000 rounds per minute (RPM) during the measurements. First, the *iR*-drop was determined by a single high-frequency (100 kHz) electrochemical impedance spectroscopy (EIS) measurement at open circuit potential. For the subsequent cyclic voltammetry (CV) and linear sweep voltammetry (LSV) measurements, 90% of the *iR*-drop was directly compensated for using Nova 5.1.7 software. The remaining 10% was balanced out by a manual correction to achieve full *iR*-compensation without risking signal oscillations. The electrocatalysts were activated by 100 CV scans in the potential range from 0.7 to 1.6 V vs RHE and a sweep rate of 50 mV s^–1^. Subsequently, three LSVs were recorded in the anodic direction from 0.7 to 1.7 V vs RHE and a sweep rate of 10 mV s^–1^. The turnover frequency (TOF) was calculated by assuming all Ni atoms (and Ru atoms for the RuO_2_ reference) at the electrode to act as catalytically active centers.

The theoretical total number of O_2_ at the turnover frequency was calculated by adapting the formula used by Habib et al. (eq 1),^19^ with the Faraday constant *F* = 96,485 C mol^–1^, *C* being the elementary charge (Coulomb), and the Avogadro constant *N*_A_ = 6.022 × 10^23^ mol^–1^.

$$i\left(\frac{\mathrm{mA}}{{\mathrm{cm}}^{2}}\right)\cdot \left(\frac{1\frac{C}{s}}{1000\;\mathrm{mA}}\right)\cdot \left(\frac{1\frac{\mathrm{mol}}{e}}{96,485C}\right)\cdot \left(\frac{1\;\mathrm{mol}\;O_{2}}{4\frac{\mathrm{mol}}{e}}\right)\cdot \left(\frac{N_{A}\mathrm{mol}\;O_{2}}{1\;\mathrm{mol}\;O_{2}}\right)=1.56\times 10^{15}\left(\frac{\frac{O_{2}}{s}}{{\mathrm{cm}}^{-2}}\right)\cdot i\left(\frac{\mathrm{mA}}{{\mathrm{cm}}^{2}}\right)$$ 1

The number of active sites was approximated with the total amount of Ni deposited on the electrode, as displayed in eq 2. The amount of deposited catalyst on the RDE (0.19635 cm^2^) was 24 μg, and the wt % of Ni was determined by ICP-OES, and 59.69 g mol^–1^ is the molecular mass of Ni.

$$\frac{\mathrm{active}\;\mathrm{Ni}\;\mathrm{sites}}{{\mathrm{cm}}^{2}}=\frac{\mathrm{wt}\;\%\;\mathrm{Ni}}{100}\cdot \frac{0.000024\;g}{0.19635\;{\mathrm{cm}}^{2}}\cdot \left(\frac{N_{A}}{59.69\frac{g}{\mathrm{mol}}}\right)$$ 2

Subsequently, the OER TOF was calculated by dividing the theoretical number of formed O_2_ at the measured current density by the number of active sites per square centimeter (eq 3).

$$\mathrm{TOF}({\mathrm{site}}^{-1}s^{-1})=\frac{1.56\times 10^{15}\left(\frac{\frac{O_{2}}{s}}{{\mathrm{cm}}^{-2}}\right)\cdot i\left(\frac{\mathrm{mA}}{{\mathrm{cm}}^{2}}\right)}{\frac{\mathrm{active}\;\mathrm{Ni}\;\mathrm{sites}}{{\mathrm{cm}}^{2}}}$$ 3

Operando EIS measurements were performed with ascending potentials at 1.10, 1.20, 1.30, 1.35, 1.40, 1.45, 1.50, 1.55, and 1.60 V vs RHE with an amplitude of 5 mV within a frequency range between 100 kHz to 0.1 Hz. Ten frequency points were acquired per decade. The obtained EIS spectra were plotted as Nyquist and Bode plots. The spectra recorded in the OER regime (1.40–1.60 V vs RHE in our case) were fitted using a modified Randle’s equivalent circuit (R­(QR)­(QR), Figure S2). For the RuO_2_ reference sample, the standard Randle’s equivalent circuit (R­(QR), Figure S2) was employed. The electrochemically active surface area (ECSA) was estimated using CV measurements with increasing sweep rates (20, 60, 100, 140, 180 mV s^–1^) in the nonfaradaic region 0.8–1.0 V vs RHE. The last of the 5 consecutive CV scans was used for data evaluation. The difference between anodic and cathodic capacitive current, extracted at 0.9 V vs RHE, was plotted against the applied scan rate. Half the slope of that linear correlation could be used to approximate the double layer capacitance (*C*_dl_, μF). The ECSA was calculated ( $\mathrm{ECSA}=\frac{C_{\mathrm{dl}}}{C_{s}}$ ), using the literature value of 0.04 mF cm^–2^ as specific capacitance (*C*_s_) for oxidic materials for OER electrocatalysts in 1 M KOH.^24^ Chronopotentiometry was performed for an extended duration >12h to test the stability of the prepared materials at a constant current of 10 mA cm^–2^.

## 3 Results and Discussion

### 3.1 Material Synthesis

The Ni–B solution precipitation reaction is known as a scalable, fast, and cheap synthesis procedure using NaBH_4_ as a reducing and exfoliation agent and boron source.^4,10,11,25^ Chen and co-workers previously prepared ternary Ni–B–P using NaH_2_PO_2_ as the secondary reducing agent and P precursor and demonstrated promising activities for hydrogenation reactions.^26–28^ While their Ni–B–P samples initially showed moderate specific surface areas (SSAs) of around 20–40 m^2^ g^–1^, they eventually managed to improve the SSA up to 82 m^2^ g^–1^, but at the same time significantly reduced the amount of B and P in the nanoalloys, which deteriorated *p*-chloronitrobenzene hydrogenation performance.^28^ We, therefore, propose an improved chemical reduction synthesis approach, aiming for increased metalloid concentrations in Ni–B–P alloys while maintaining high SSA, to enhance the OER activity. The experimental conditions of the Ni–B–P synthesis, which is based on an aqueous chemical reduction procedure with optimized concentrations of Ni­(NO_3_)_2_, NaBH_4_, and NaH_2_PO_2_, are described in detail in the Section 2. As displayed in Scheme 1, P-free Ni–B, Ni–B–P, and P-rich Ni–B–P-2 samples were prepared by using no, regular, or twice the amount of NaH_2_PO_2_, respectively. A pronounced effect linked to annealing temperatures was found by comparing materials in the as-made (AM, no annealing) state and samples that were annealed at 150, 200, 250, 300, and 350 °C (labeled, e.g., Ni–B_AM, Ni–B–P_300, or Ni–B–P-2_150) in N_2_ atmosphere. The prepared materials were thoroughly characterized regarding their bulk and surface composition, texture, and porosity, and were benchmarked for the electrocatalytic OER.

**Scheme 1:**
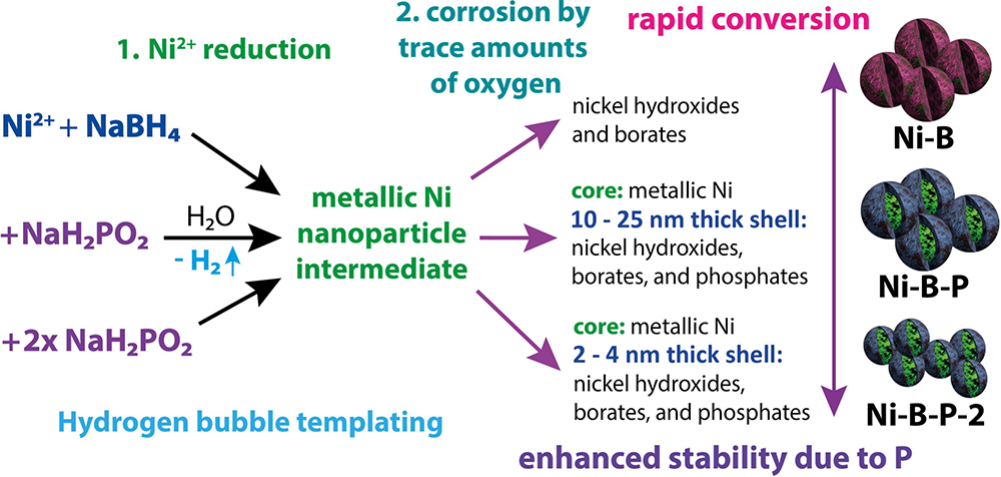
Formation Pathways of the Ni–B, Ni–B–P, and Ni–B–P-2 Electrocatalysts via the Solution Precipitation Reaction

Thermogravimetric analysis (TGA) of the as-made samples in N_2_ atmosphere (Figure S1b, Table S1) revealed significant weight loss for the Ni–B sample, primarily linked to the removal of adsorbed water and the dehydration of H_3_BO_3_ side product into B_2_O_3_, which is expected to occur below 200 °C.^29^ A continuous weight loss up to 500 °C is linked to the conversion of Ni­(OH)_2_ into NiO.^30^ Based on these results, it can be assumed that a significant residual amount of Ni­(OH)_2_ will be present in the prepared Ni–B samples due to the low applied annealing temperatures ranging from 150 to 350 °C. The inclusion of phosphorus appears to progressively decrease the formation of Ni­(OH)_2_ as the weight loss for Ni–B–P and Ni–B–P-2 is reduced at all temperatures, but especially above 200.°C

Wide-angle powder X-ray diffraction (PXRD) analysis (Figure 1) corroborated these observations and revealed the amorphous nature of the as-made samples for Ni–B, Ni–B–P, and Ni–B–P-2, with significant α-Ni­(OH)_2_ formation in the Ni–B sample. α-Ni­(OH)_2_ was only a minor fraction in Ni–B–P and is not visible in the Ni–B–P-2 diffractograms. Ni–B–P and Ni–B–P-2 have broad amorphous signals at around 45° 2θ, which can be related to the (111) diffraction plane of Ni. Based on previous reports, the chemical reduction of Ni^2+^ with NaBH_4_ yields a mixture of Ni_*x*_B and Ni^0^ nanoparticles.^11,31–33^ Due to the facile oxidation of these compounds in water, especially in the presence of dissolved oxygen, nickel borates, NiO, and Ni­(OH)_2_ are rapidly formed during the washing procedure.^31,34^ Phosphorus, known for its corrosion protection properties,^35^ was employed to control the surface oxidation and obtain core–shell nanoparticles. Upon annealing under N_2_ at 150–350 °C for 1h, all samples undergo significant changes. Most prominently, the big, broad reflections for metallic Ni become very sharp, indicating the conversion of amorphous Ni_*x*_B into larger Ni^0^ crystallites and B_2_O_3_, as suggested elsewhere.^32^ The Ni­(OH)_2_ reflections present in the Ni–B and Ni–B–P samples steadily decrease with increasing annealing temperature. Interestingly, the α-Ni­(OH)_2_ (001) reflection is shifting from 11.8° toward higher 2θ angles, which can be explained by the removal of interlayer water and shrinkage of the crystal cell along the *c*-axis.^36^ In the Ni–B sample, annealing at 350 °C produced amorphous reflections at around 37.3 and 43.3 θ that could fit to NiO (PDF = 98-009-2127), the dehydration product of Ni­(OH)_2_. At 300 °C, the formation of trace amounts of Ni_3_B becomes visible in the diffractogram of Ni–B–P.^11,37^ Due to the very limited miscibility of B and P in Ni metal, one can assume that a heterogeneous amorphous phase mixture of Ni and Ni_3_B is separated into discrete, larger crystallites, which become detectable by PXRD upon annealing at 300 °C. In agreement with previous studies, the Ni_3_B phase disappears at 350 °C.^12,13^ The reaction of Ni_3_B with traces of oxygen or water vapors originating from concurring dehydration of Ni­(OH)_2_ and B­(OH)_3_ to nickel borates at 350 °C is a plausible explanation as Ni_3_B is easily oxidized, even at room temperature.^32,34,38^ The phosphorus-rich Ni–B–P-2 samples do not exhibit reflections related to α-Ni­(OH)_2_, but otherwise are quite comparable to Ni–B–P up to annealing temperatures of 250 °C, with increasingly crystalline Ni^0^ as the main feature. At 350 °C, the Ni–B–P-2 samples display the formation of Ni_3_P. Ni–B–P-2_300 and Ni–B–P-2_350 additionally show reflections of B­(OH)_3_, which is surprising since B­(OH)_3_ would dehydrate or evaporate at that temperature.^29,39^ It may be rationalized that, as a result of the elevated annealing temperatures, the formation of larger B_2_O_3_ domains is facilitated due to phase separation. This B_2_O_3_ is rather quickly rehydrated when it is exposed to room humidity to yield boric acid. Figure S3 displays a more detailed visualization of Ni–B_300, Ni–B–P_300, and Ni–B–P-2_350, with the reference patterns used for Ni, α-Ni­(OH)_2_, β-Ni­(OH)_2_, Ni_3_B, Ni_3_P, and B­(OH)_3_. The more crystalline samples, Ni–B–P_300, Ni–B–P_350, Ni–B–P-2_300, and Ni–B–P-2_350, have been further analyzed by Rietveld refinement (Figures S4–S7).

**Figure 1:**
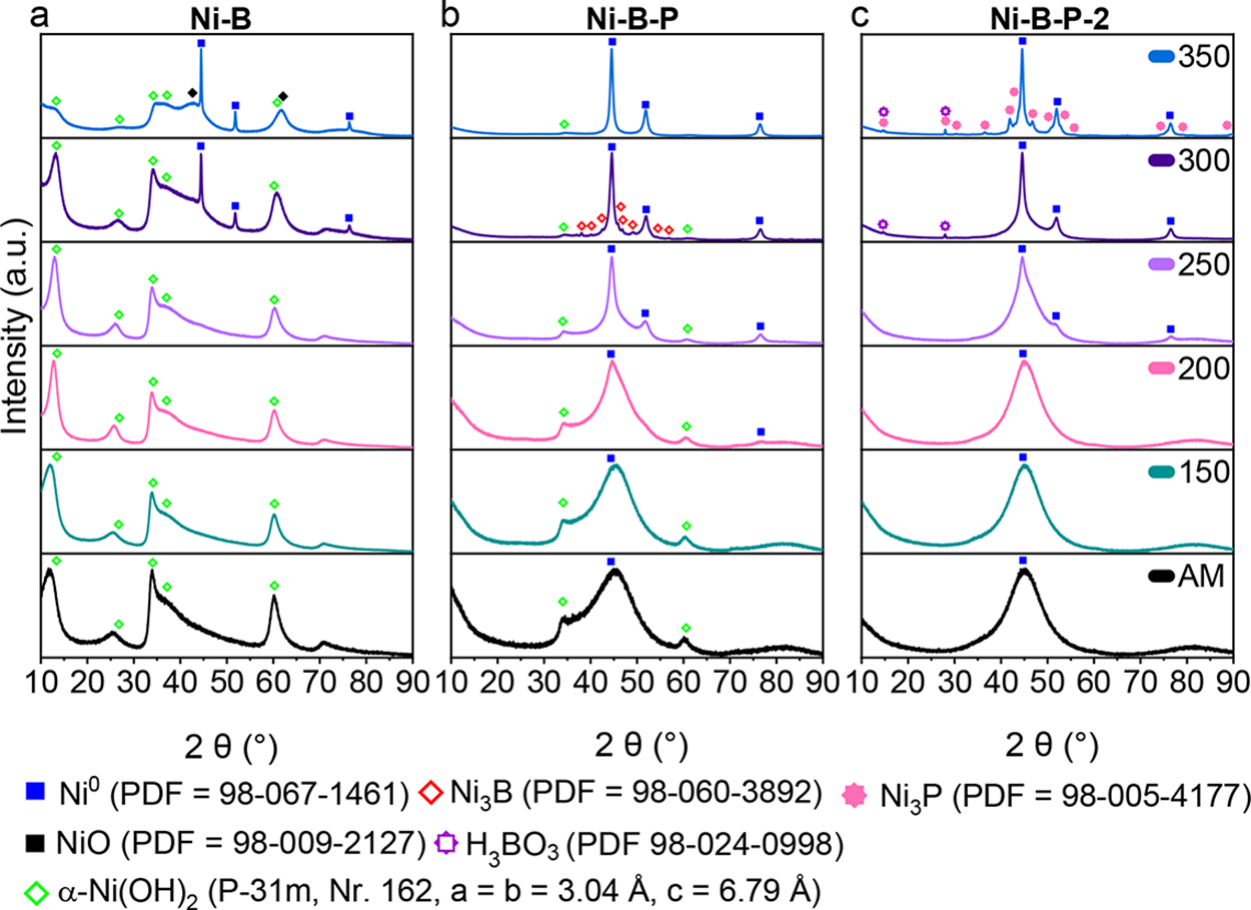
Powder XRD patterns of (a) Ni–B, (b) Ni–B–P, and (c) Ni–B–P-2 as-made and annealed at 150, 200, 250, 300, and 350 °C in the nitrogen atmosphere.

Raman spectroscopy (Figure S8a) underscored the significant changes in Ni–B upon annealing. The Ni–B_AM vibrational modes at 325, 443, 529, 626, 888, 1006, and 1046 cm^–1^ agree well with the literature data on α-Ni­(OH)_2_.^36,40–42^ The dehydration into NiO after annealing at 300 °C is evident from the 363, 516, 680, 911, and 1052 cm^–1^ vibrational modes, typical for NiO.^43,44^ NiO appears to be present in the Ni–B–P samples and Ni–B–P-2_AM, as they exhibit a spectrum very similar to that visible in Figure S8d. The Ni–B–P samples, as shown in Figure S8b, display two contributions visible as shoulders of a broad vibrational band located at about 493 and 530 cm^–1^. Upon increasing the annealing temperature, the signal for species at 493 cm^–1^ is reduced, while the band centered at 530 cm^–1^ becomes more pronounced. Previous in situ electrocatalysis Raman studies reported similar signals linked to the conversion of Ni^2+^–O to Ni^3+^–O species, e.g., NiOOH.^42,45,46^ Significant changes are visible in Figure S8c for the Ni–B–P-2_AM and Ni–B–P-2_300 samples. Upon annealing at 300 °C, the broad Raman signals are replaced with weak signals. This could indicate the replacement of nickel oxides with amorphous phosphates and borates, which we will further discuss in the XPS section below.

X-ray fluorescence spectroscopy (XRF), scanning electron microscopy-based energy dispersive X-ray spectroscopy (SEM–EDX) elemental mapping, and inductively coupled plasma optical emission spectroscopy (ICP-OES) were performed for bulk chemical analysis. The results are summarized in Tables S2–S4 and indicate the significant influence of NaH_2_PO_2_ addition on the final composition, namely, a decrease in boron and oxygen and an increase in phosphorus and nickel, while the thermal treatment barely alters the bulk chemical composition. The SEM–EDX elemental maps (Figures S9 and S10) show a homogeneous elemental distribution across the three different materials. Traces of Na can be linked to the NaBH_4_ precursor. Furthermore, the SEM images in Figure S11 display the highly porous nature of the Ni–B–P and Ni–B–P-2 AM samples.

For further detailed morphological characterization, Ni–B_300, Ni–B–P_300, and Ni–B–P-2_300 were characterized by TEM. The results are displayed in Figures 2, 3, S12, and S13. Bright-field TEM images (Figure 2) of Ni–B_300 revealed clusters of layered nanosheets with an interlayer distance of 6.6 Å, corresponding to the α-Ni­(OH)_2_ (001) PXRD reflection at 13.18° 2θ. Ni–B–P_300 and Ni–B–P-2_300 both show agglomerated core–shell nanoparticles with a major difference in their shell thickness and appearance. The crystallite *d*-spacing analysis of the TEM image shown in Figure 2 confirmed the presence of a metallic Ni core in the Ni–B–P_300 and Ni–B–P-2_300 samples, with *d*-spacings corresponding to the Ni^0^ (111) direction. The Ni–B–P_300 sample exhibits shells with significantly lower density or lighter elements as compared to the cores, and the *d*-spacings could match the (101) plane of α-Ni­(OH)_2_ and the (121) and (211) planes of Ni_3_B. As displayed in Figure 3, Ni–B–P_300 displayed inhomogeneous, roughly 8–25 nm thick shells comprised of voluminous 3D nanosheet-like clustered layers, which are comparable to the architecture of the Ni–B-300 sample, with the exception that they do not appear to be ordered. The TEM of the P-rich Ni–B–P-2 displays metal cores, which are coated by a very uniform and compact, only roughly 2–4 nm thick shell (Figure S13). Energy dispersive X-ray spectroscopy (EDX) mapping in scanning TEM (STEM) visualized the homogeneous distribution of Ni and O across the Ni–B_300 particles (Figure S14). Figure S15 indicates that in Ni–B–P_300, oxygen is preferably found on the particle surface, while Ni is more abundant in the core. For Ni–B–P-2_300, an increased P content could be confirmed with EDX, and Figure S15 displays a higher abundance of P in the shell/surface of the particles.

**Figure 2:**
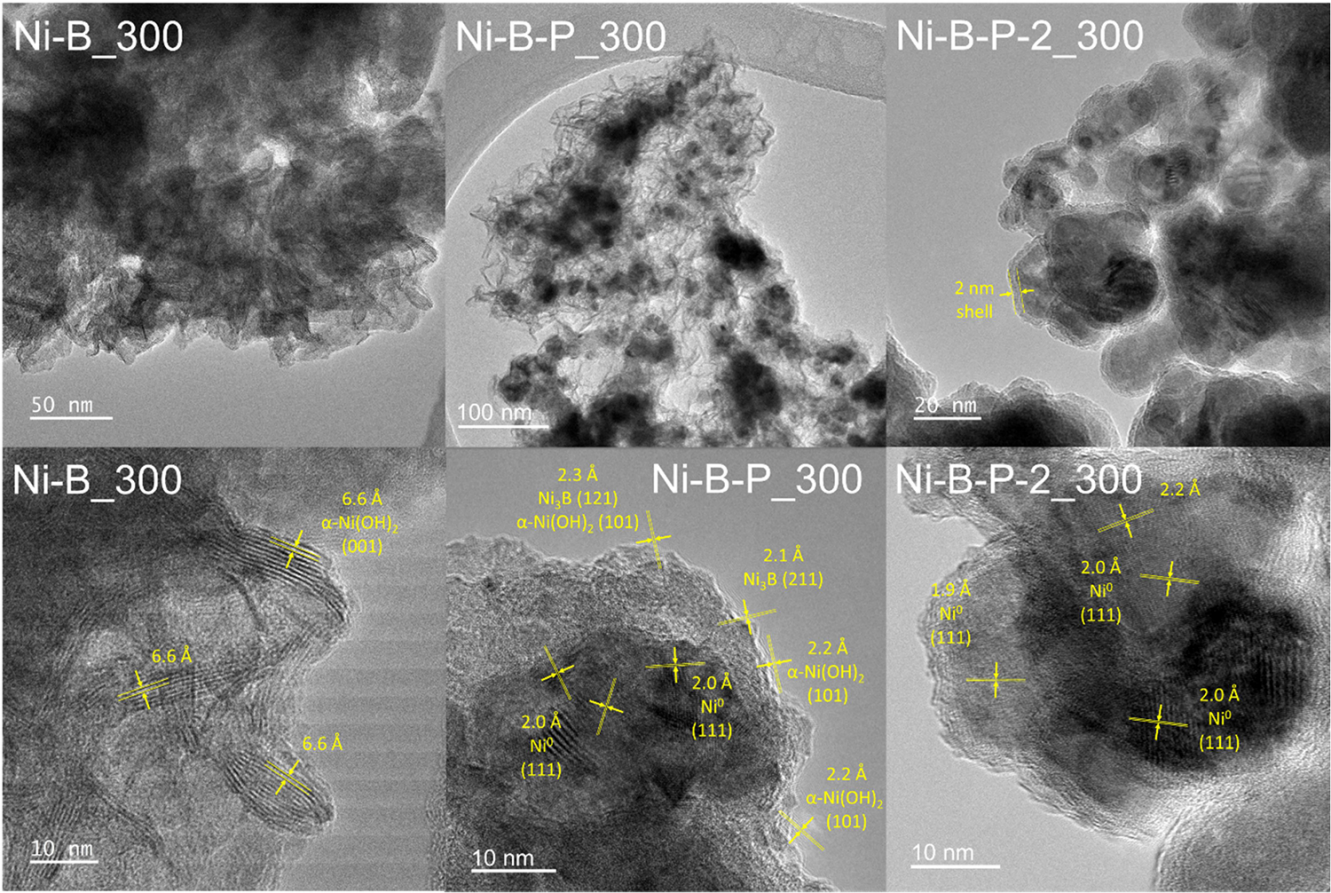
High-resolution TEM images of Ni–B_300, Ni–B–P_300, and Ni–B–P-2_300.

**Figure 3:**
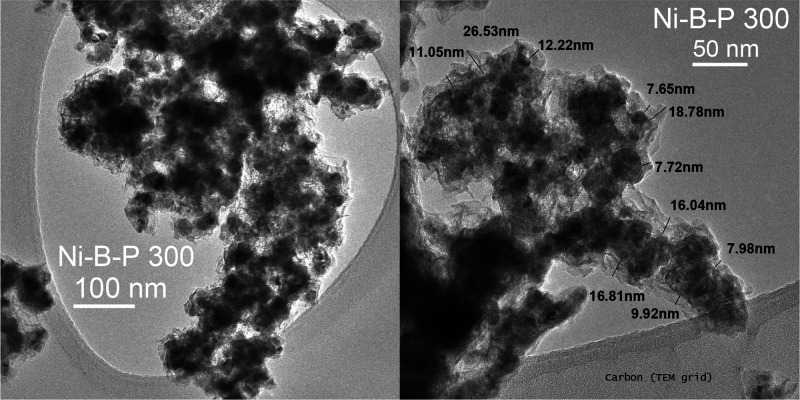
High-resolution TEM images displaying the varying shell thickness observed in Ni–B–P_300 core–shell nanoparticles.

### 3.2 Surface Properties of the Electrocatalysts

N_2_ physisorption revealed well-developed mesoporosity for the Ni–B and Ni–B–P samples, as visible from the type IV isotherms and their H2 and H3 hysteresis loops, respectively. In contrast, the Ni–B–P-2 samples exhibit comparably low N_2_ adsorption and lack a distinct mesopore feature (Figure S16). This is reflected in the high Brunauer–Emmett–Teller (BET) SSA for Ni–B and Ni–B–P samples of 93–168 and 48–96 m^2^ g^–1^, respectively, whereas the P-rich Ni–B–P-2 samples showed comparably low 23–35 m^2^ g^–1^ BET SSAs. The nonlocal density functional theory (NLDFT) pore size distributions (PSD, Figure S17; Tables S5 and S6) reveal dominant pore sizes of 6 nm for Ni–B, which are unaltered by the annealing procedure. The Ni–B–P samples exhibit broader PSDs centered at around 6.1–10.5 nm and depict a trend toward decreasing pore size upon annealing. The Ni–B–P samples had the largest NLDFT pore volumes (0.38–0.41 cm^3^ g^–1^), followed by Ni–B–P-2 (0.28–0.25 cm^3^ g^–1^), and Ni–B (0.13–0.22 cm^3^ g^–1^).

X-ray photoelectron spectroscopy (XPS) was performed to analyze the surface electronic states of Ni–B_300, all Ni–B–P samples, and Ni–B–P-2_300. All XPS spectra and tables (Figures S18 and S19, Tables S7–S16) and a detailed discussion of the XPS results can be found in the SI, while the main findings and the most significant XPS spectra are summarized in the following paragraph and Figure 4. The XPS survey scans (Figure S18, Tables S7 and S8) revealed significant deviations between surface and bulk elemental compositions (Tables S2–S4), with higher B, lower Ni, and comparable levels of P on the surface, suggesting different core and shell compositions. The P and O surface concentrations slightly decreased, while B concentrations rose with higher Ni–B–P annealing temperatures. The increased amount of NaH_2_PO_2_ in the synthesis of Ni–B–P-2_300 resulted in more P and B, and less O and Ni on the surface as compared to the other samples. High-resolution spectra revealed shifts in B 1s, O 1s, and P 2s peaks toward higher binding energies upon increasing the P amount and annealing temperature, indicating the formation of B_2_O_3_ and P_2_O_5_.^8,38,47,48^ The O 1s peaks assigned to NiO and Ni­(OH)_2_ were the most abundant in Ni–B_300, which is also reflected by the stronger Ni^2+^ signals in the Ni 2p_3/2_ spectra. The Ni^0^ peak also shifted toward higher binding energies upon annealing and increased P content, and reflects the formation of Ni–P and Ni–B species and modifications of the electronic structure of metallic Ni.^34^ Furthermore, the shifts in Ni L_3_M_45_M_45_ auger peak shapes indicate Ni­(OH)_2_ dehydration upon annealing and the formation of highly metallic species in Ni–B–P-2_300.^49^

**Figure 4:**
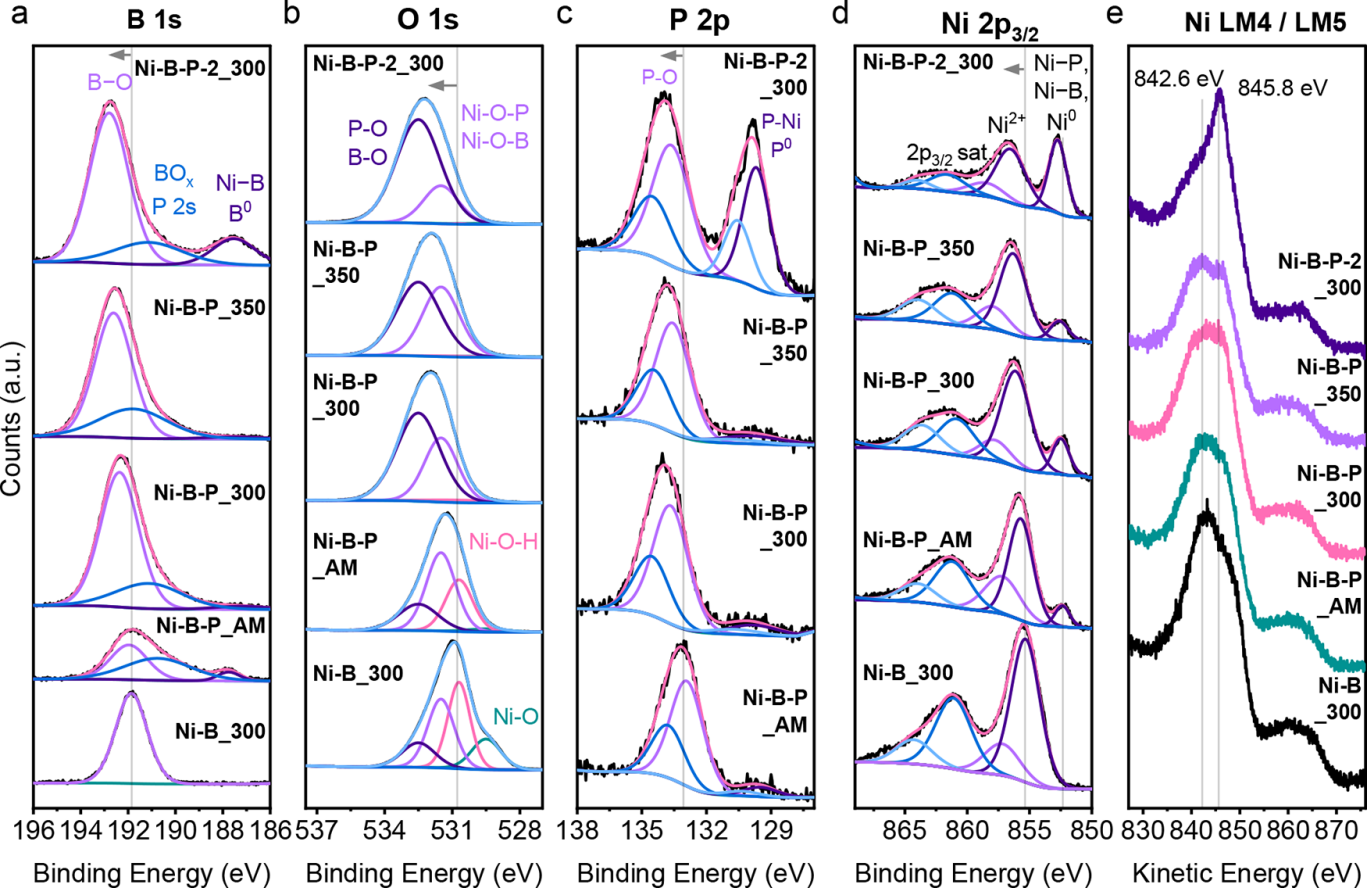
(a) B 1s and (b) O 1s (c) P 2s, (d) Ni 2p_3/2_, and (e) Ni L_3_M_45_M_45_ Auger HR-XPS spectra of Ni–B_300, Ni–B–P as-made, 300, and 350, and Ni–B–P-2_300. Due to the plethora of possible oxygen-containing compounds, the deconvolution was performed by assigning one peak at 531.5 eV to Ni–O–B/–P and the O–B/–P peak at 532.5 eV, resembling metal-free borates and phosphates.^8,47^ The graphs are displayed in a stacked form to improve visibility.

Low energy ion scattering spectroscopy (LEIS) performed with He ions accelerated at 3 keV allowed us to reveal the chemical composition of the outermost surface layer for all Ni–B–P samples, Ni–B_300, and Ni–B–P-2_300. The LEIS spectra are displayed in Figure S20, and the quantification result is summarized in Table S17. Surprisingly, the Ni–B_300 sample revealed an intense Na peak, linked to the use of NaBH_4_, which corresponds to roughly 12 atom % of Na, while the other samples did not display that feature. In SEM-EDX mapping (Figure S9), trace amounts of Na (0.5 atom %) could be observed for Ni–B_AM, so it can be concluded that Na was highly accumulated at the outmost surface of the Ni–B samples while it was not very abundant in the ‘bulk’ of the powder. One can speculate that Na content was below the detection limit for the P-containing samples because of the core–shell structure, which, with its likely decreased diffusion paths through the thinner shell (Figures 2 and S13), facilitated Na removal in the washing steps. The intense signals accounting for Ni and O at roughly 2270 and 1100 eV are accompanied by weak B and P peaks at around 776 and 1770 eV. The annealing temperature influenced the LEIS P surface concentrations, which became smaller with increasing annealing temperature. Furthermore, the P-rich Ni–B–P-2_300 displayed a slight decrease in Ni and O surface concentrations and clearly has more exposed P atoms, such as Ni–P or P–O, judging from XPS, at the very top surface. Boron quantification is difficult due to the very weak signal intensities recorded in the measurements. However, the LEIS spectra suggest that the native Ni–B–P_AM had the highest amount of B on its surface (11 atom %) and that Ni–B–P-2_300 exhibited an increased concentration of B atoms at the surface, which agrees with the XPS results. The decrease in B with an increasing annealing temperature does not contradict the B 1s trends observed in XPS measurements when considering that the temperature treatment led to an oxidized surface. We learned from LEIS that the quite abundant borate species in the nanoparticle shell are mostly Ni- and O-terminated at the surface.

### 3.3 Electrochemical Characterization and Testing for OER

Cyclic voltammetry (CV) was used as an activation step in electrocatalyst testing. The CV profiles of Ni–B, Ni–B–P, and Ni–B–P-2 (Figures 5 and S21–S29) show similar redox behavior. A reversible Ni^2+/3+^ redox peak (A_1_/A_2_ for oxidation and C_1_/C_2_/C_3_ for reduction) is observed before electrocatalytic water oxidation (A_3_). The precatalytic redox reaction can be described in eq 4:

$$\mathrm{Ni}(\mathrm{OH}{)}_{2}+{\mathrm{OH}}^{-}⇄\mathrm{NiOOH}+H_{2}O+e^{-}$$ 4

**Figure 5:**
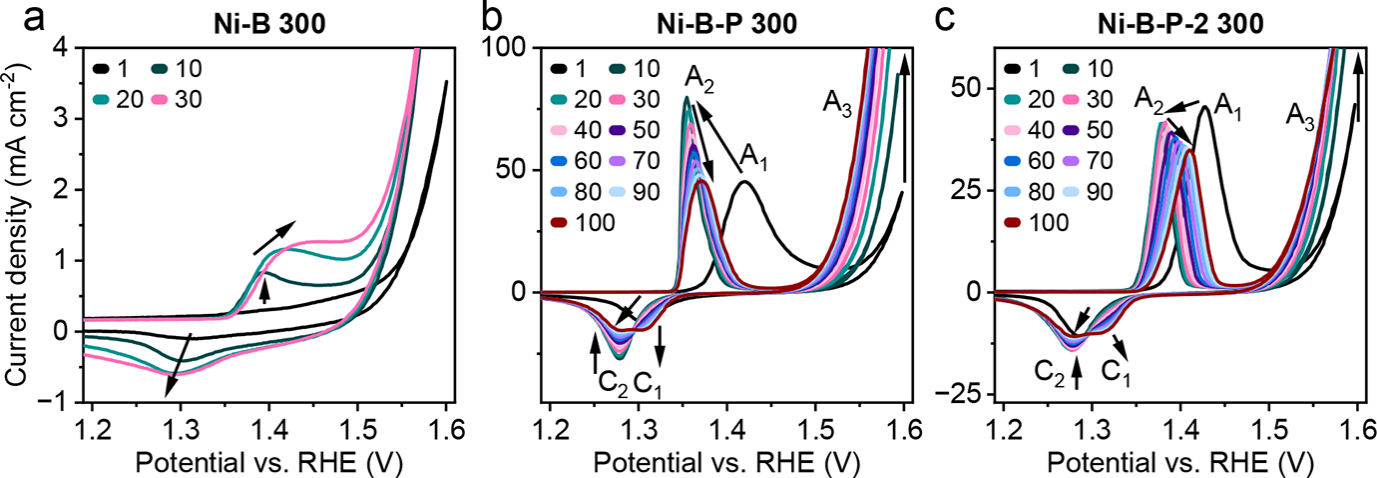
CV scans obtained for (a) Ni–B_300, (b) Ni–B–P_300, and (c) Ni–B–P-2_300 during the initial activation protocol. The cycle numbers of the depicted scans are displayed in the top left corner of the graphs, respectively.

It is very crucial for the subsequent electrocatalytic water oxidation (labeled A_3_) as the formed NiOOH (Ni^3+^) is considered to be the active species in Ni-based OER electrocatalysts. The first cycle displayed a precatalytic oxidation (A_1_) that shifts to lower potentials in the following cycle (labeled A_2_), indicating a transformation of the catalyst surface. In cycles 2–10, the Ni–B–P and Ni–P–B-2 samples (Figures S25 and S28) display a continuous increase in the A_2_ peak area, indicating further growth of the NiOOH layer, which is accompanied by a steady decrease in the OER onset potential and increasing current densities at the anodic switching potentials–evidently an activation toward the OER. Roughly after the first 10 CV cycles (Figures S26 and S29), the shift of the A_2_ peak was reversed and started moving toward the anodic direction for the rest of the CV activation procedure. At the same time, the formation of an additional reduction peak at more anodic potentials (C_3_) was observed for the Ni–B–P and Ni–B–P-2 samples, which can be linked to the formation of β-NiOOH.^42,50–52^ The formation of this species is described in the widely accepted Bode Scheme (Figure S30) that illustrates the Ni­(OH)_2_/NiOOH redox switching behavior. Following this scheme, the initially present hydrated α-Ni­(OH)_2_ film (as determined by PXRD and TEM) is electrooxidized to γ-NiOOH during CV. Upon exposure to strongly alkaline solutions, however, α-Ni­(OH)_2_ may be converted into the dehydrated β-Ni­(OH)_2_.^53,54^ Subsequently, the β-Ni­(OH)_2_ is oxidized into β-NiOOH in the anodic sweep, and improves the OER performance since it is a more active OER catalyst than γ-NiOOH. This can be attributed to its improved electrical conductivity, which results from the smaller *d*-spacing between the NiOOH layers.^55^ However, the Ni–B samples did not display such behavior, and it is likely that, due to the absence of the C_3_ reduction peak, no β-Ni­(OH)_2_/β-NiOOH redox-couple was formed, which could explain the limited OER performance. Due to their poor stability, cycling was stopped after 10 and 40 cycles for Ni–B_AM and Ni–B_300, respectively, to conserve them in their active form for the subsequent measurements.

Prior works have demonstrated that nickel phosphate facilitates the formation of highly active β-Ni­(OH)_2_ during cycling and that this process is accompanied by complete P dissolution and a gradual shift of redox peaks toward higher potentials.^56,57^ The redox reactions (eqs 5 and 6) of Ni^2+^ and Ni^3+^ phosphate were proposed to lead to a continuous leaching of phosphate anions upon cycling, while the NiOOH formed in eq 5 forms its own Ni­(OH)_2_/NiOOH redox couple, which accumulates at the electrode surface (eq 7), as follows^57^:

$${\mathrm{Ni}}_{3}({\mathrm{PO}}_{4}{)}_{2}+3{\mathrm{OH}}^{-}\to 2{\mathrm{NiPO}}_{4}+\mathrm{NiOOH}+H_{2}O+3e^{-}$$ 5

$$3{\mathrm{NiPO}}_{4}+3e^{-}\to {\mathrm{Ni}}_{3}({\mathrm{PO}}_{4}{)}_{2}+{\mathrm{PO}}_{4}^{3-}$$ 6

$$\mathrm{NiOOH}+H_{2}O+e^{-}⇄\mathrm{Ni}(\mathrm{OH}{)}_{2}+{\mathrm{OH}}^{-}$$ 7

The continuous shift in the CV measurements and the retention of small amounts of P (as will be discussed in the post-OER XPS section below) indicate that the P dissolution is still ongoing, and alteration of the electronic configuration of the electrocatalyst surface is not complete after 100 cycles. Thus, precise control of P in the catalyst is necessary to achieve the best activities. Similar reactions have been proposed for the boron leaching in transition metal borides as displayed in eq 8 ^37,58^ :

$${\mathrm{NiB}}_{x}+(6x+2){\mathrm{OH}}^{-}\to \mathrm{Ni}(\mathrm{OH}{)}_{2}+x{\mathrm{BO}}_{3}^{3-}+3xH_{2}O$$ 8

In the case of the Ni–B–P and Ni–B–P-2 electrocatalysts, the discrepancy between A_2_ and C_2/3_ peak areas (Figure S31; Tables S18 and S19) suggests that every CV cycle leads to more oxidation than reduction, which underscores the leaching of B and P as described above. The leaching of electroactive species further explains the decreasing A_2_ peak areas during the activation procedure (Figures S26 and S29), leaving behind more active Ni^3+^ centers for efficient OER.

Linear sweep voltammetry (LSV) scans were recorded at low scan speeds (10 mV s^–1^) to evaluate the performance of the electrocatalysts for the OER and are summarized in Figure 6a–c and Table S20. Ni–B–P_300 was the most active electrocatalyst, with an OER overpotential of only 281 mV at 10 mA cm^–2^, a high current density of 500 mA cm^–2^ at 1.65 V, and a low Tafel slope of 44 mV dec^–1^ (Figure S32). For the Ni–B–P samples, a clear correlation of OER metrics can be seen by continuous improvement with the applied annealing temperature, however, a drastic decrease has been observed for the Ni–B–P_350 sample. Compared to that, the P-rich Ni–B–P-2 samples showed lower deviations upon annealing. Especially the overpotential at 10 mA cm^–2^, ranging between only 289–292 mV, was hardly influenced. The fastest OER kinetics were recorded for the Ni–B–P-2_150 sample, yielding the same Tafel slope as that of Ni–B–P_300 and a high current density of 300 mA cm^–2^. The advantage of combining Ni with both B and P is apparent in the clearly decreased OER performance of the P-free Ni–B samples. The doubling of the Tafel slope hints at the formation of less active sites, which have a different rate-determining step. Another explanation could be the clearly lower electrical conductivity of the Ni­(OH)_2_-rich Ni–B samples as compared to the samples with metallic Ni cores.^56^

**Figure 6:**
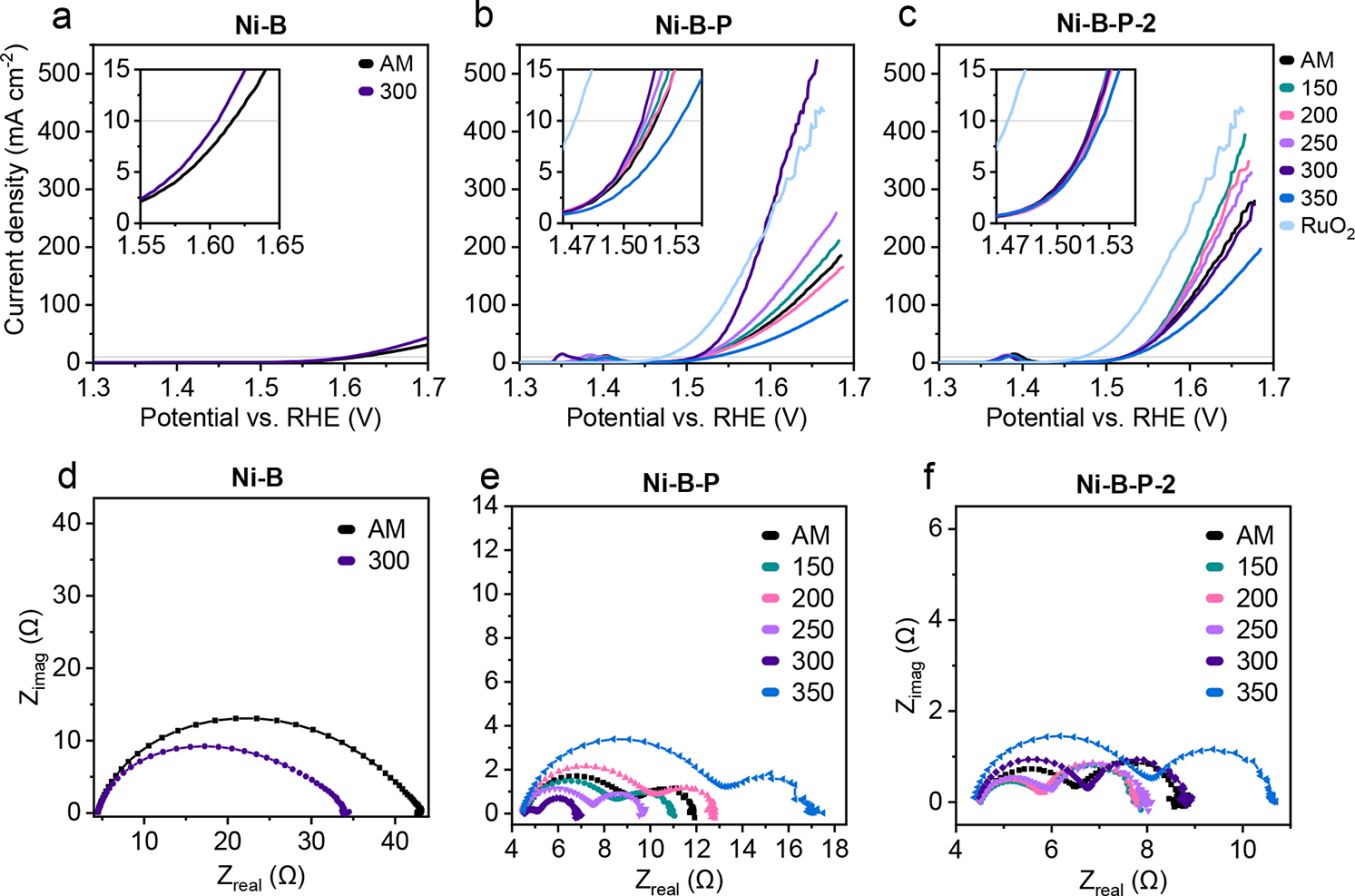
OER LSV curves of (a) Ni–B, (b) Ni–B–P, and (c) Ni–B–P-2 samples with insets to display the OER onset potential region, and Nyquist plots of (d) Ni–B, (e) Ni–B–P, and (f) Ni–B–P-2 samples recorded at 1.6 V vs RHE.

CV scans conducted with ascending scan speeds (20–180 mV s^–1^) were performed to estimate the electrochemical double-layer capacitance (*C*_dl_, Figures S33–S36). The electrochemical active surface area (ECSA), which was appraised from the *C*_dl_ as described in the SI, shows very similar values in the range between 0.7–0.9 cm^2^ indicating that the observed activity trends are rather linked to different intrinsic activities than differences in the exposed catalyst surface area at the electrode (Table S21). The quite large standard deviation of the ECSA values can be explained by the nature of the sample with metal nanoparticles and voluminous shells that have quite different surface areas, and the error related to using CV scans for *C*_dl_ determination.

The OER turnover frequency (TOF) at 1.65 V vs RHE was calculated as an intrinsic activity descriptor and compiled in Table S20. Compared to the other Ni–B–P samples, which exhibit OER TOFs of around 0.2 s^–1^, Ni–B–P annealed at 250 °C (0.3 s^–1^) and especially 300 °C (0.9 s^–1^) indicate a massive improvement in intrinsic activity, while annealing at 350 °C had a detrimental effect and yielded only 0.1 s^–1^. The Ni–B–P-2 samples show the best OER TOF when annealed at 150 °C (0.5 s^–1^), but range between a narrow interval of 0.3–0.5 s^–1^. Since we calculated the TOF based on the assumption that all Ni sites are exposed as catalytically active centers, the TOFs are generally underestimated. In Figure S37, the OER LSV current density is normalized to the BET SSA and the ECSA. The BET SSA normalized plots display superior performance of the Ni–B–P-2 samples and, together with the good TOF values, signify the improved intrinsic activity of these samples.

Commercial RuO_2_ with a BET SSA of 65 m^2^ g^–1^ was employed as reference material for the OER. Being an active commercial noble metal catalyst, it displayed a low overpotential of only 242 mV to achieve 10 mA cm^–2^ and outmatched the prepared catalysts in that regard (Figure 6; Tables S37, S38, S20, and S21). However, some of the prepared catalysts, and especially Ni–B–P_300, displayed lower Tafel slopes than the RuO_2_ reference 50 mV dec^–1^). Hence, Ni–B–P_300 outperformed RuO_2_ at current densities exceeding 250 mA cm^–2^. The high activity of RuO_2_ is further reflected by a TOF of 1.2 s^–1^ and is almost matched by that of Ni–B–P_300 (0.8 s^–1^). As expected for tests in alkaline media, the RuO_2_ sample is not very stable, as displayed in the CP measurement, and it is easily outperformed by the tested Ni–B–P and Ni–B–P-2 samples (Figure S39).

Operando EIS was conducted for the activated Ni–B–P_300 and Ni–B–P-2_300 samples to understand the reaction processes occurring in the precatalytic region up to the onset and active OER (1.1–1.6 V vs RHE). In the Nyquist plots depicted in Figure S40, large impedances are visible for measurements in the nonfaradaic region 1.1–1.3 V. At 1.35 V, the emergence of a small semicircle for Ni–B–P_300 (*R*_1_ = ∼2.1 Ω), and a larger one for Ni–B–P-2_300 (*R*_1_ = ∼34.0 Ω) signals a decrease in the surface oxide layer resistance due to the Ni^2+^ electrooxidation. The sudden decrease in impedance visible in the 1.35 V measurement of the Ni–B–P-2_300 sample is linked to the ongoing oxidation of the surface at this potential. As suggested by the lower R_1_ values, the Ni–B–P_300 sample benefits from a lower oxide film resistance (Table S22). The oxidation of Ni­(OH)_2_ species is completed at 1.40 V and leads to a further decrease of the oxide layer resistance *R*_1_. The measurements conducted at 1.40 V or higher displayed a second semicircle at lower frequencies, which can be linked to the charge transfer resistance (*R*_ct_) of the OER reaction, and rapidly decreased with increasing applied potential. These plots were fitted using the modified Randle’s equivalent circuit (R­(QR)­(QR)) (Figure S2), and the results are displayed in Table S22. Additionally, the Bode plots (Figure S41, Table S23) visualize the decreasing oxide layer impedance for both samples. The lower the phase angles of the high frequency (Θ_1_) and low frequency (Θ_2_) peaks become, the lower the oxide layer resistance becomes and the faster the OER reaction occurs, respectively.

Additional EIS measurements were performed at 1.6 V vs RHE to compare the activated samples regarding the electron transfer steps. The Nyquist plots of the Ni–B samples exhibited one depressed semicircle with a broad shoulder, and the Ni–B–P and Ni–B–P-2 samples exhibited two well-defined semicircles (Figure 6d–f, Tables S24 and S25). The Bode plots in Figure S42 with decreased values of *R*_1_ and Θ_1_ for Ni–B–P_300 hint at a lower oxide film resistance, which facilitates the OER as evidenced by the decreased *R*_ct_ and Θ_2_ (Table S26). The EIS data of Ni–B–P-2 samples suggest that their oxide film impedance and OER *R*_ct_ are less influenced by the annealing step than their Ni–B–P counterparts, proving once again their potency as OER catalysts, outperformed only by Ni–B–P_300. The Ni–B–P and Ni–B–P-2 samples annealed at 350 °C depict significantly increased *R*_1_ oxide film resistance and an increased level of the OER *R*_ct_. This and the relatively low A_2_ precatalytic oxidation peak charge discovered for these samples (Figure S31, Tables S18 and S19) could explain their comparably poor OER performance.

It is commonly accepted that Fe impurities found in the electrolyte are deposited in unpurified KOH electrolytes. The 1 M KOH electrolyte used in this study was found to contain 69.4 ppb of iron (ICP-MS), which could be enough to interfere with the electrocatalytic performance of nickel. The post-OER STEM-EDX mapping (Figure S43 vs Figures S9 and S10, comparison of EDX spectra in Figure S44) and pre- vs post-OER XPS survey spectra (Figures S45b and S46e (Fe 2p overlaps with the Ni LM4/LM5 Auger peaks)) do not indicate detectable amounts of deposited Fe; however, a future study will be performed to assess the influence of Fe on Ni–B–P by performing electrocatalytic tests in purified electrolytes.

### 3.4 Electrocatalytic Stability and Conversion

Catalyst stability during prolonged operation was tested with overnight chronopotentiometry measurements with an applied current density of 10 mA cm^–2^. The results are summarized in Figure S39, where it becomes evident that all tested Ni–B–P_300 catalysts become further activated within the first hours of CP testing, and good stability was revealed. The Ni–B–P-2_300 sample displayed a comparable short ‘activation’ period, followed by signs of degradation as indicated by a slightly increasing overpotential. An explanation for this decrease in activity could lie in the increased thickness of the shell, as observed with post-OER (100 CVs, LSV, ECSA, EIS in 1 M KOH, 25 °C) TEM measurements of Ni–B–P_300 and Ni–B–P-2_300 samples, leading to a decrease in the electrical conductivity. As displayed in Figure S47, Ni–B–P_300 and Ni–B–P-2_300 still exhibit distinct crystalline nanoparticle cores with *d*-spacings matching metallic Ni in the case of Ni–B–P_300, and Ni and Ni_3_P for the Ni–B–P-2_300 post-OER sample. The shell thickness of the Ni–B–P_300 was relatively unaffected, while the Ni–B–P-2_300 metal nanoparticles have visibly expanded shells from initially roughly 2 to 4–6 nm. Both shells revealed *d*-spacings matching α- or β-Ni­(OH)_2_. STEM-EDX mapping suggests significant P dissolution during the OER measurements for both samples (Figures S43 and S44). These results are supported by the comparison of XPS measurements recorded for Ni–B–P_300 supported on Ni-foil before and after 15h of CP (10 mA cm^–2^) (Figures S45 and S46, Tables S27–S35). P and B contents decreased from 2.4 and 20.1 atom % to only 1.1 and 6.7 atom %, respectively, while Ni and O were enriched at the surface by 6.0 and 8.7 atom %. Further, the O 1s HR-XPS spectra display a significant increase in Ni–OH, Ni–O, and Ni–O–B/P at the cost of phosphate and borate species (Table S31).

## 4 Conclusions

A room-temperature aqueous solution precipitation synthesis of Ni–B–P was developed to yield core–shell nanoparticles with a Ni metal core and phosphates and borates at the shell surface. Pronounced electrochemical activation of the Ni–B–P samples was confirmed using CV and operando EIS, leading to an excellent OER performance. It was discovered that P and B were mostly leached but favored the in situ generation of highly active β-NiOOH sites. Our work highlights the critical role of active site engineering in achieving high-performance OER electrocatalysts and sets the stage for further advancements in noble metal-free catalysts through a rapid, facile, and scalable synthesis approach. As the next step, we aim to investigate the synthesis of Ni–B–P core–shell nanoparticles with more precisely defined elemental distributions, such as the incorporation of a thin P-rich film by adjusting the synthesis procedure. Additionally, we plan to explore the precise composition of the metallic Ni core, investigate the effects of adding Fe or other metals, and utilize in situ techniques to study catalyst activation while also exploring scalability beyond the gram scale.

## Supplementary Material



## Notes

Extended discussion of XPS results, TGA results, XRD peak assignment and Rietveld refinement, Raman spectra, SEM-EDX elemental mapping images, SEM images, bright-field TEM images, STEM-EDX elemental mapping images, N_2_ physisorption isotherms and results, NLDFT pore size distribution graphs, XPS survey scans, HR-XPS deconvoluted spectra of B 1s, O 1s, P 2p, Ni 2p and Ni LMM, LEIS spectra, CV measurements, Bode scheme depicting the Ni­(OH)_2_ chemistry, CV electrical charge consumed by oxidation and reduction as a function of cycle number, tabulated results (overpotential, current density at 1.65 V vs RHE, Tafel slope and TOF at 1.65 V vs RHE) of LSV measurements, Tafel plot analysis, electrochemical capacitance (*C*_dl_) determination using CV measurements, LSV measurements normalized to BET SSA and ECSA, electrocatalytic OER benchmark of RuO_2_ reference, Nyquist plots, used equivalent circuits, Bode plots, chronopotentiometry measurements, bright-field TEM and STEM-EDX elemental maps after OER testing, STEM-EDX spectra, XPS survey and HR-XPS spectra of samples before and after OER testing, and additional references 59,60 (PDF)
