# Supplementary material for: In-Situ Formation of High-Performance β‑NiOOH OER Electrocatalysts Using Boron and Phosphorus-Enriched Ni Core–Shell Nanoparticles
Source: ACS Appl Mater Interfaces. 2025 May 16;17(21):30773–84. doi: 10.1021/acsami.4c22116 (PMC12123570; doi:10.1021/acsami.4c22116)
Supplement: Supplementary file 1 [file am4c22116_si_001.pdf]

Supporting Information

In-Situ Formation of High-Performance β-NiOOH OER Electrocatalysts Using Boron and Phosphorous-Enriched Ni Core-Shell Nanoparticles

Patrick Guggenberger [a,b], Prathamesh Patil [c,d], Bernhard Fickl [e], Christian M. Pichler [c,d], Bernhard C. Bayer [e], Martin Stockhausen [f], Thilo Hofmann [f], Guenter Fafilek [g], Freddy Kleitz [a*]

[a] Department of Functional Materials and Catalysis, Faculty of Chemistry, University of Vienna, Währinger Straße 42, 1090 Vienna, Austria.

[b] Vienna Doctoral School in Chemistry (DoSChem), University of Vienna, Währinger Straße 42, 1090 Vienna, Austria.

[c] CEST Centre of Electrochemical and Surface Technology, Viktor Kaplan-Straße 2, 2700 Wiener Neustadt, Austria.

[d] Institute of Applied Physics, Technische Universität Wien, Wiedner Hauptstraße 8-10, 1040 Vienna, Austria.

[e] Institute of Materials Chemistry, Technische Universität Wien, Getreidemarkt 9, 1060 Vienna, Austria.

[f] Department for Environmental Geosciences, Centre for Microbiology and Environmental Systems Science, University of Vienna, Josef-Holaubek-Platz 2, 1090 Vienna, Austria.

[g] Institute of Chemical Technologies and Analytics, Technische Universität Wien, Getreidemarkt 9, 1040 Vienna, Austria.

* Corresponding author: E-mail: freddy.kleitz@univie.ac.at.

Table of Content

1. Extended discussion of materials characterization

1.1 X-ray photoelectron spectroscopy

2. Figures S1 to S47

3. Tables S1 to S35

1. Extended discussion of materials characterization results

1.1 X-ray photoelectron spectroscopy

The surface electronic states of Ni-B_300, all Ni-B-P samples, and Ni-B-P-2_300 were studied with X-ray photoelectron spectroscopy (XPS). The survey scans (Fig. S11, Table S7) and the derived surface elemental compositions largely deviate from the bulk elemental compositions. All samples reveal significantly enhanced B and reduced Ni concentrations. Phosphorous had similar surface concentrations as compared to the bulk results, but XPS revealed that, similarly to Ni, it is continually diminished on the surface with increasing annealing temperatures in Ni-B-P, while boron surface concentration was augmented via annealing. XPS also enabled quantification of surface oxygen, which ranged from 56.5 − 47.8 at% and slightly decreased with increasing annealing temperature, which might be linked to the removal of adsorbed water and dehydration of hydroxide species. Compared to its Ni-B-P_300 analog, Ni-B 300_has 12.1 at% Ni and 4.5 at% O increase at the surface, while B is decreased by 14.2 at%. Ni-B-P-2_300 exhibited an increased amount of P (+ 2.4 at%) and B (+7.0 at%), and consequently decreased O (− 4.2 at%) and Ni (− 5.3 at%) at the surface, which rationalizes into an accumulation of phosphates and borates at the surface. The high-resolution (HR-) XPS B 1s core level spectrum in Fig. 3a and Fig. S12a displays elemental B or B−Ni at binding energies around 187.5 − 188.0 eV. A broad P 2s core level contribution is observed at about 191 eV for all Ni-B-P samples and the Ni-B-P-2_300 sample. A further peak at binding energies of 191.8 − 193.2 eV was assigned to boron oxide and is shifting to higher binding energies with increasing annealing temperature and phosphorous content (Tab. S8). Coupled with the observation of decreasing Ni content and increase of O−B species in the O 1s spectrum, it can be explained that in the core level B 1s spectrum, the B−O shift results partly from the decreased Ni^{2+} charge-compensating effect and electronic density and partly form the conversion of nickel borates to B_2O_3.[1,2] Whilst surface boron becomes more abundant with increased annealing temperature and phosphorous content, we observed a decrease in B^0/B−Ni

to B−O peak area ratio (Tab. S9) reflecting a significant reduction of B^0/B-Ni from 13 (Ni-B-P AM) to only 2.4 at% (Ni-B-P_350). The complex O 1s core level spectra (Fig. 3c, Fig. S12c, Tab. S10-S11) include contributions from oxides (Ni−O, 529.5 eV), hydroxides (Ni−OH, 530.7 eV), phosphates, and borates. Only the Ni-B_300 sample seemed to have a significant amount of surface NiO and the highest Ni−OH contribution of all samples (16.4 and 35.3 at% of Ni species, respectively). The O 1s spectra reveal how the addition of NaH_2PO_2 reduced the amount of initially formed $Ni(OH)_2$ and that it was gradually converted with increasing annealing temperature. Surprisingly, no increase in NiO could be seen, as the product of Ni dehydration. Due to the abundance of B and P on the surface, the two peaks assigned to Ni−O−P and Ni−O−B at 531.5 eV and O−B and O−P at 532.5 eV become the dominant species, and we could observe how the latter became more pronounced at temperatures above 250 °C. Furthermore, it was the highest for Ni-B-P-2_300 and could signify the segregation of B_2O_3 and P_2O_5 at the surface.[3,4] As depicted in Fig. 3c (additionally in Fig. S12c and Tab. S12), the P 2p spectrum was deconvoluted into a weak metallic (Ni−P, 130.5 eV) and an intense oxidic phosphorous doublet peak (P−O, 133.0 eV) with peak splitting of 0.87 eV. Annealing of the Ni-B-P samples appears to have slightly increased the relative phosphide amount from 8.5 to 11.4 P at% (Tab. S13). Interestingly, the Ni-B-P-2_300 sample had a very distinct nickel phosphide/phosphate formation resulting in 58.2 P at.%. Similar to the observed trends for B 1s and O 1s, the P 2p P−O peak shifted with increasing annealing temperature and phosphorous content from 133.0 eV to 134.0 eV (Fig. 3c, Fig. S12c, Tab. S12), supporting increasing contents of P_2O_5.[5] The Ni $2p_{3/2}$ HR−XPS spectrum also followed this general trend (Fig. 3d, Fig. S12d). The Ni^{2+} peak at 855.3 eV for Ni-B-300 gradually shifted towards 857.0 eV for Ni-B-P-2_300, hinting at the formation of Ni^{2+} species with less electron withdrawing (more boron and phosphorus-containing) environment. The peak at 852.3 eV was assigned to metallic Ni, which is supported by the PXRD results. Also, this peak is shifted to higher binding energies upon thermal annealing and higher P content (853.1 eV for Ni-B-P-2_300, Tab. S14), which supports the formation of Ni−P and Ni-B species and indicates active modification of the electronic

structure of the metallic Ni.[6] Furthermore, Ni $L_3M_{45}M_{45}$ Auger peaks were recorded and displayed two peaks at kinetic energies of 842.6 and 845.8 eV (Fig. 3e and Fig. S12e). The dominant peak for Ni-B_300 is located at 842.6 eV and is gradually superimposed by the 845.8 eV contribution. Biesinger et al. have shown that metallic Ni exhibits a sharp $L_3M_{45}M_{45}$ peak at 846.2 eV, whilst NiO and $Ni(OH)_2$ were exhibiting a rather broad peak and an Auger peak maximum at 843.9 and 842.6 eV, respectively.[7] Compared to $Ni(OH)_2$, NiO was described to exhibit a more pronounced peak shoulder at higher kinetic energies (around 845.8 eV), which fits very well with the trend we could observe with our measurements. Whilst for Ni-B_300, $Ni(OH)_2$ was quite abundant, the shoulder indicating transformation into NiO species became more distinct in Ni-B-P_AM and steadily increased with annealing temperature. For Ni-B-P-2_300, the Auger peak shape is an indication of the presence of significant amounts of metallic nickel.[7]

2. Figures S1 to S47:

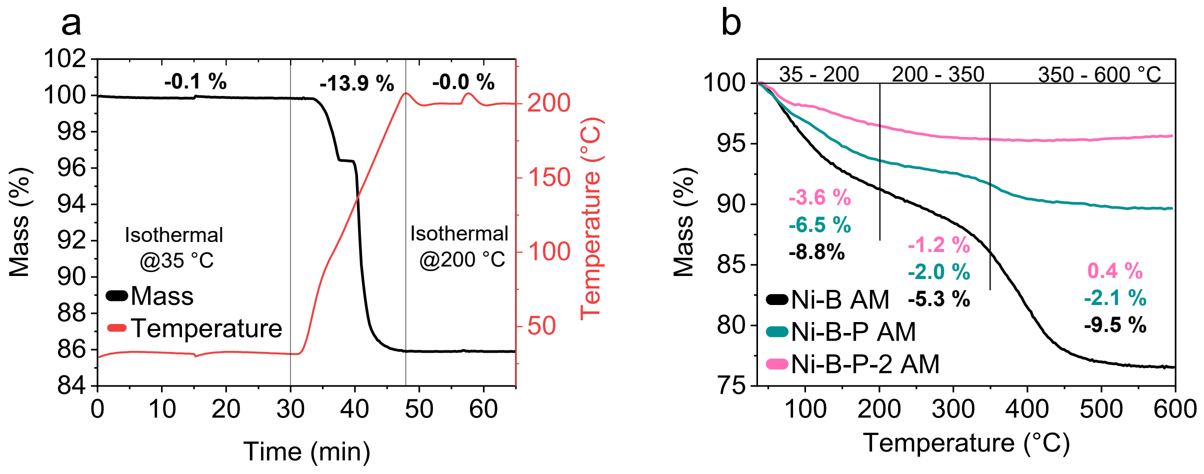

Figure S1. Thermogravimetric analysis of a) $NaH_2PO_2 \cdot x\ H_2O$ precursor and b) as-made Ni-B, Ni-B-P, and Ni-B-P-2 samples performed in nitrogen atmosphere.

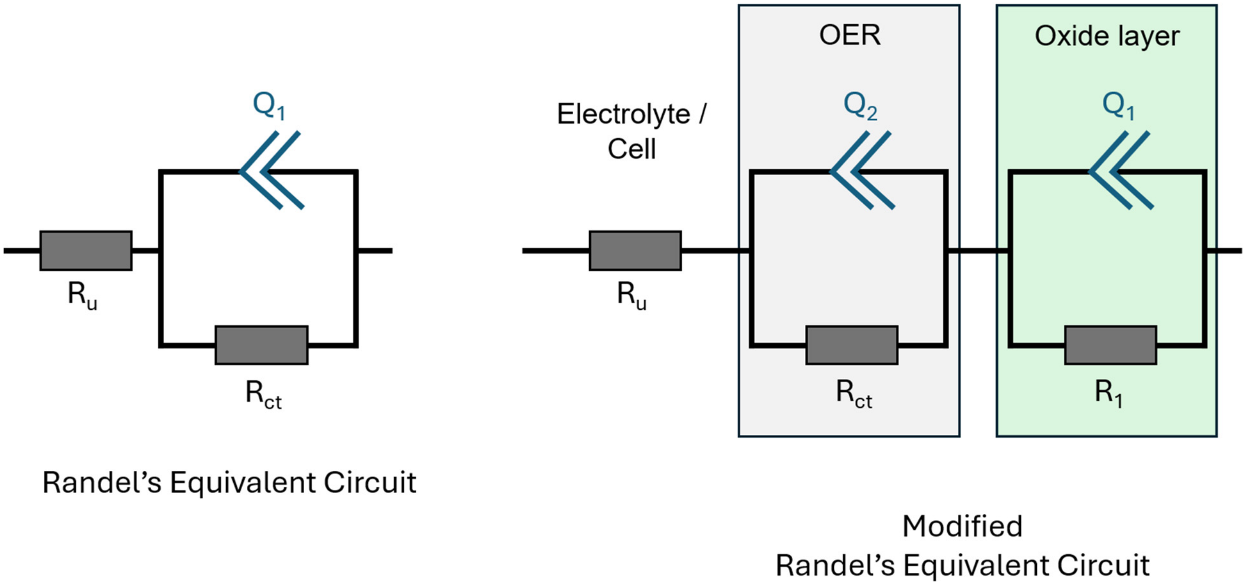

Figure S2. Equivalent circuits used for EIS evaluation, including the uncompensated resistance R_u of the electrolyte, charge transfer resistance (R_{ct}) of the OER reaction and oxide layer resistance (R_1).

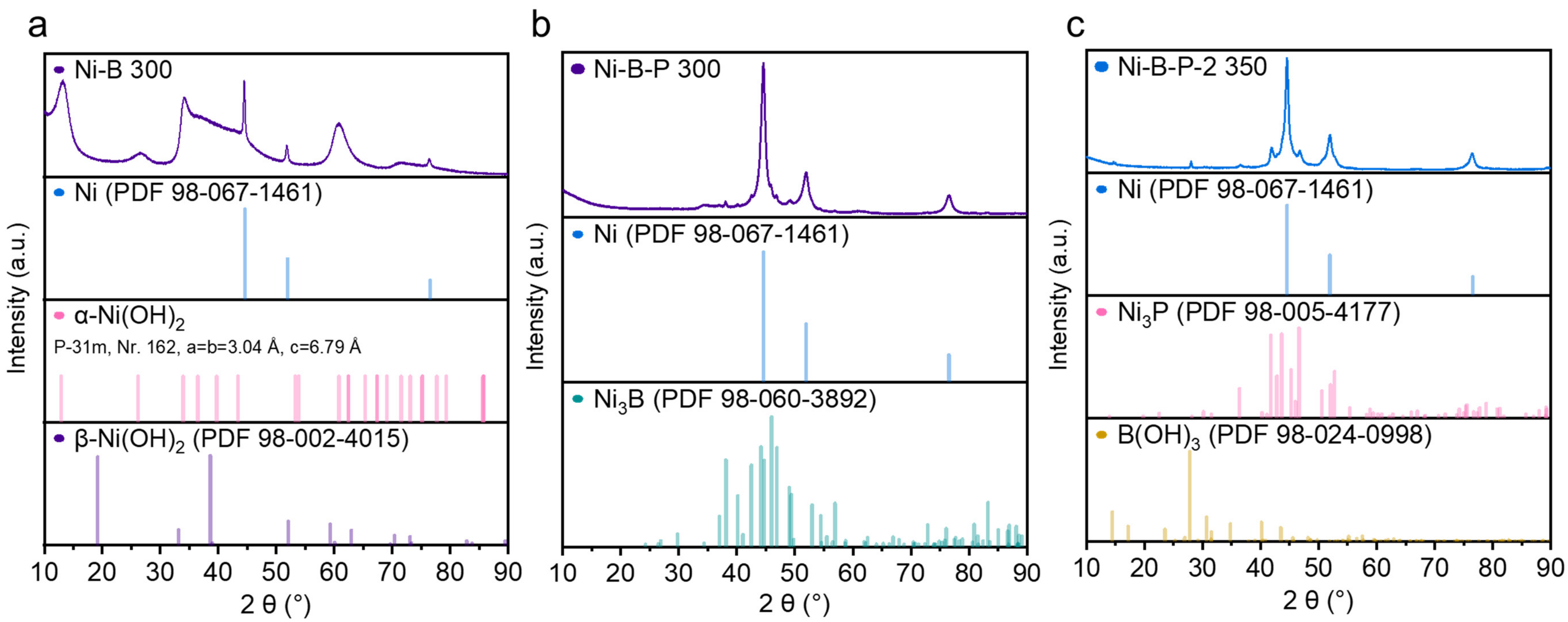

Figure S3. PXRD reference patterns for a) Ni-B_300, b) Ni-B-P_300, and c) Ni-B-P-2_350.

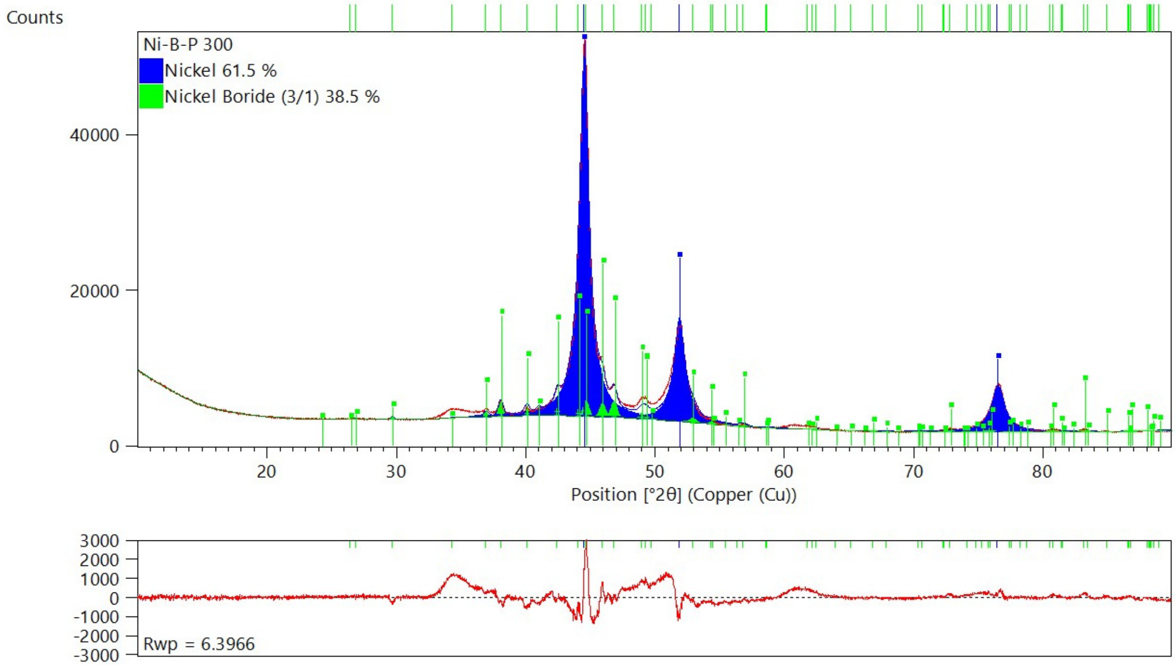

Figure S4. Rietveld refinement performed on the Ni-B-P_300 sample confirmed metallic Ni (PDF = 98-067-1461) and Ni_3B (PDF = 98-060-3892).

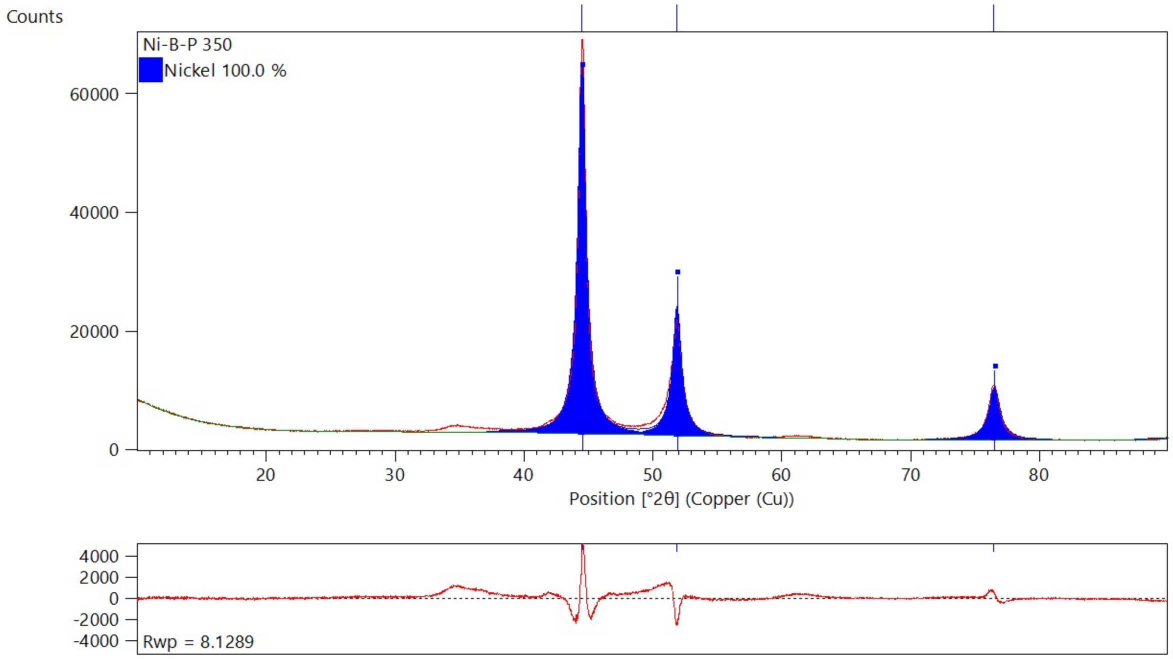

Figure S5. Rietveld refinement performed on the Ni-B-P 350 sample confirmed metallic Ni (PDF = 98-067-1461).

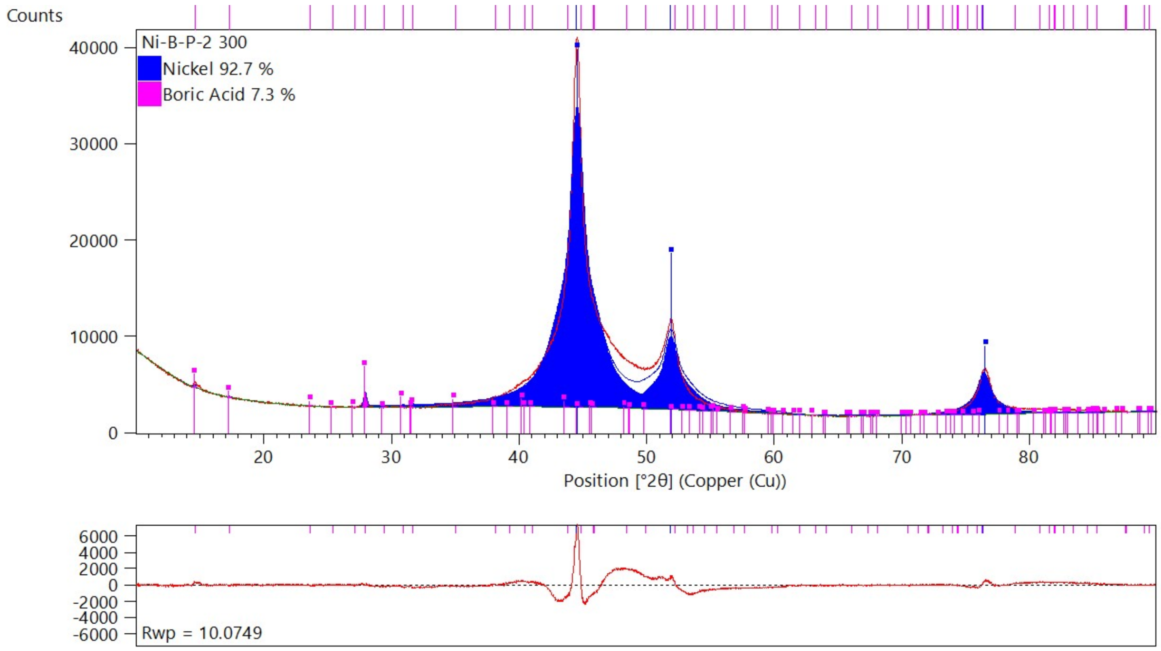

Figure S6. Rietveld refinement performed on the Ni-B-P-2_300 sample confirmed metallic Ni (PDF = 98-067-1461) and boric acid (PDF = 98-024-0998).

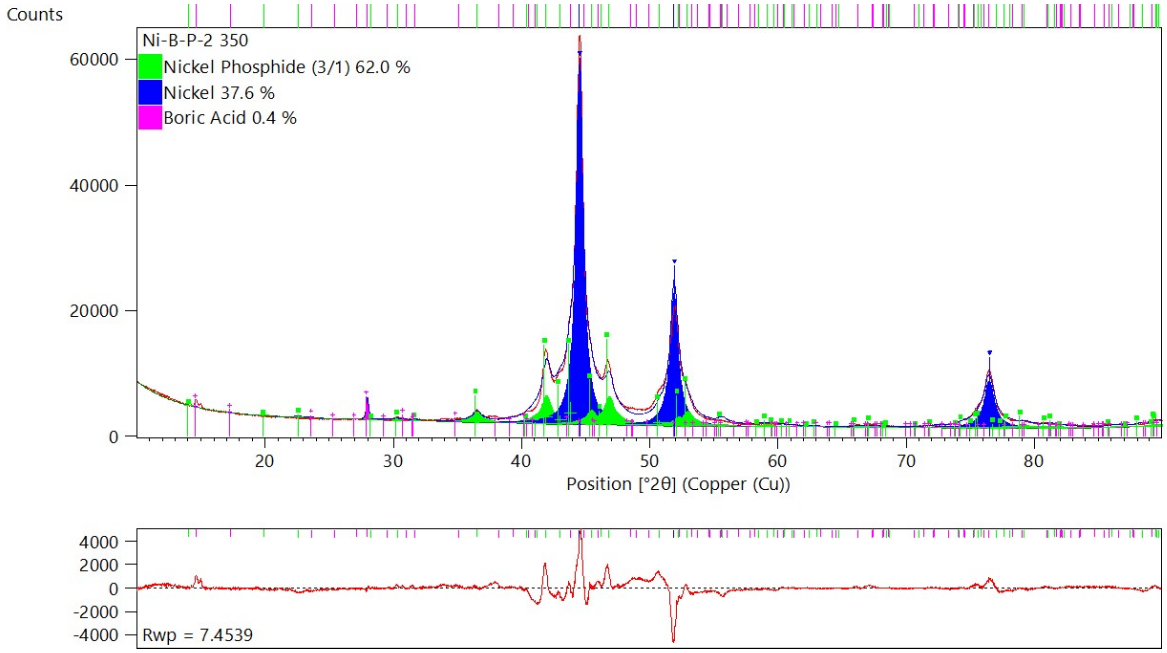

Figure S7. Rietveld refinement performed on the Ni-B-P-2_350 sample confirmed metallic Ni (PDF = 98-067-1461), Ni_3P (PDF = 98-005-4177), and boric acid (PDF = 98-024-0998).

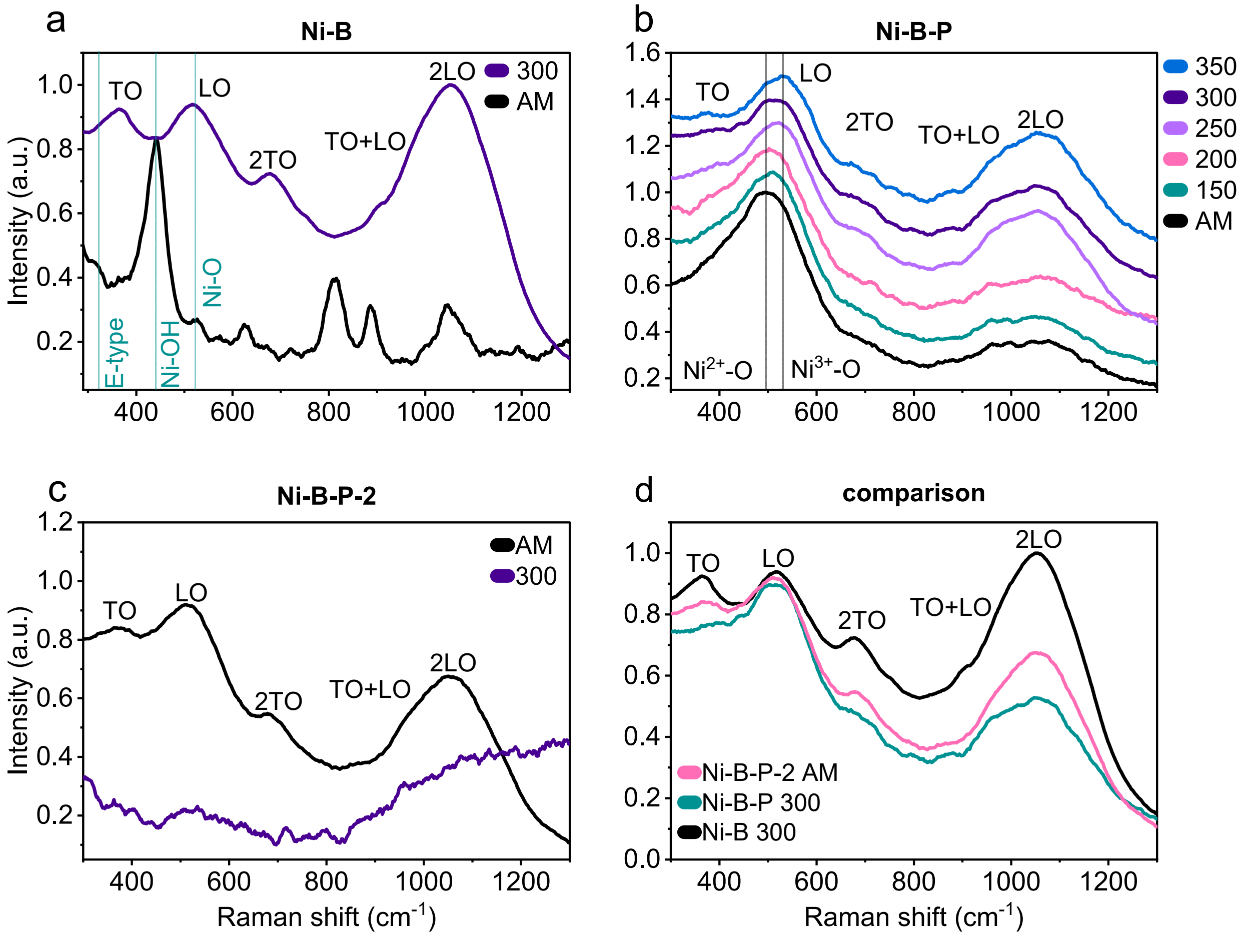

Figure S8. Raman spectra of a) Ni-B as-made and 300, b) Ni-B-P_AM, 150, 200, 250, 300, and 350, c) Ni-B-P-2_AM and 300, and d) comparison of Ni-B_300, Ni-B-P_300, and Ni-B-P-2_AM with the 5 typical Ni-O vibration Raman modes assigned as LO and TO (longitudinal and transversal modes), and E-type Ni-OH lattice, Ni-O, and Ni-OH deformation vibrations for $Ni(OH)_2$. The spectra are displayed in stacked format to improve visibility.

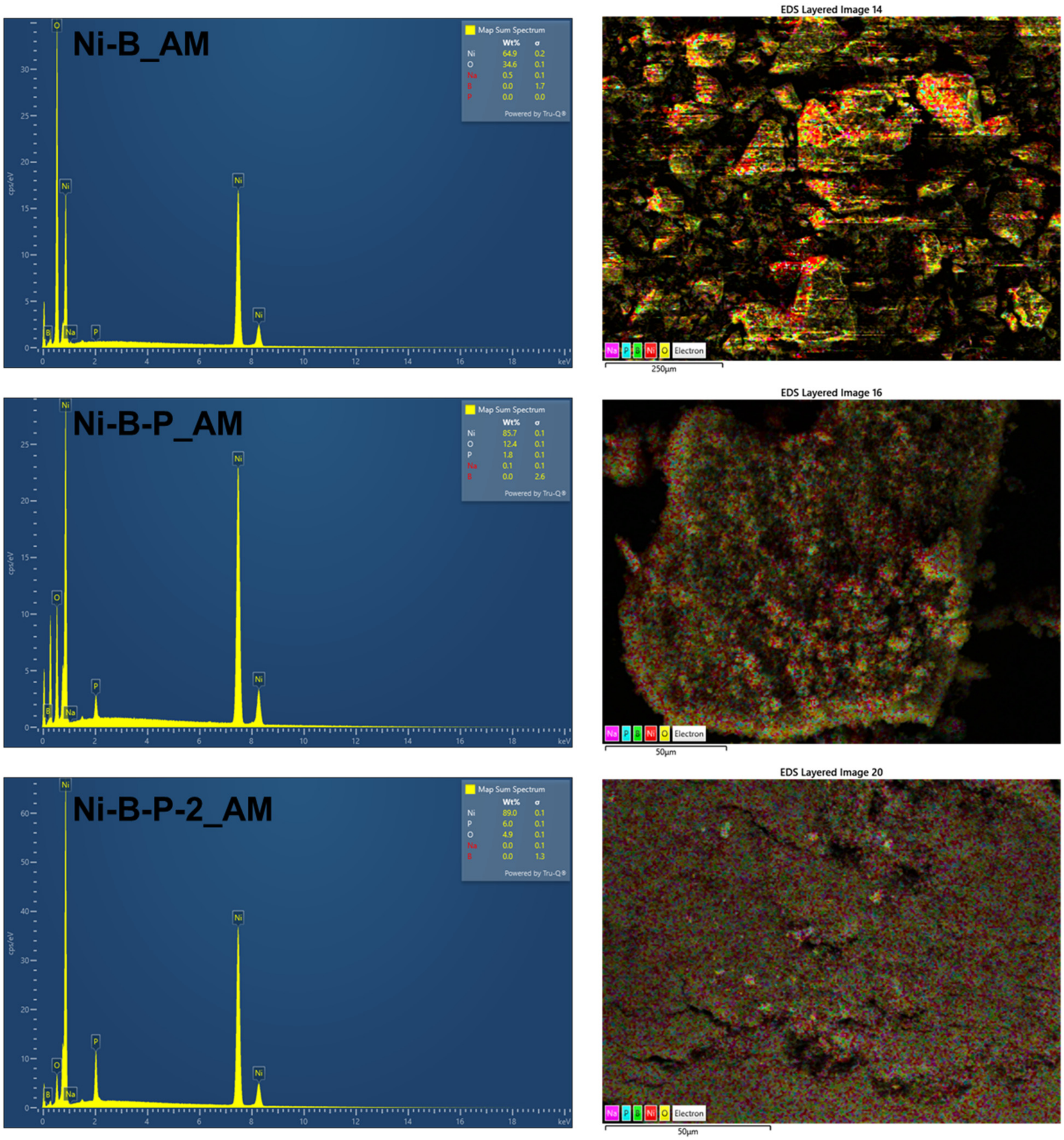

Figure S9. SEM-EDX elemental mapping spectra and overlay of elemental contributions of Ni-B_AM, Ni-B-P_AM, and Ni-B-P-2_AM.

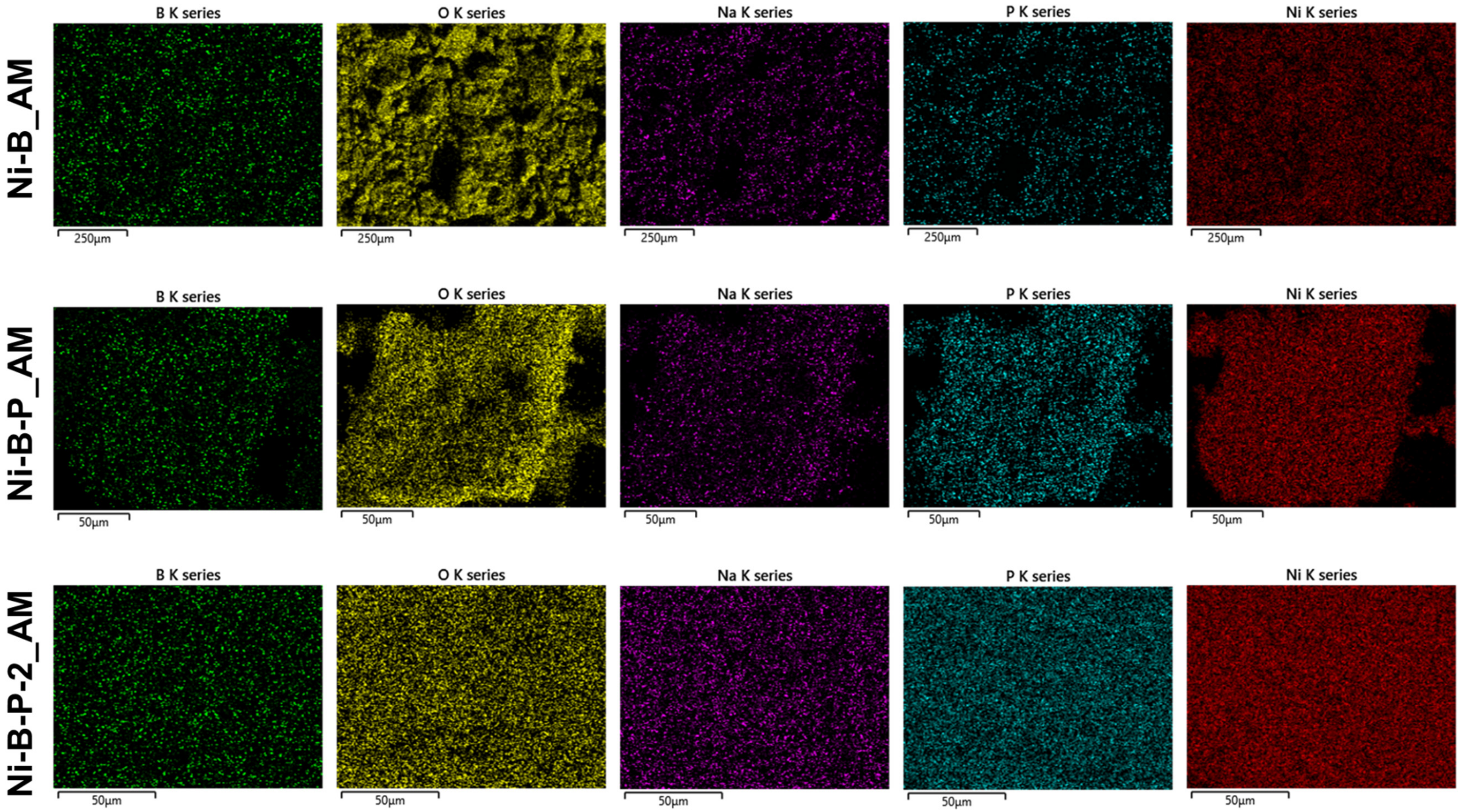

Figure S10. SEM-EDX B, O, Na, P, and Ni elemental maps recorded for Ni-B_AM, Ni-B-P_AM, and Ni-B-P-2_AM.

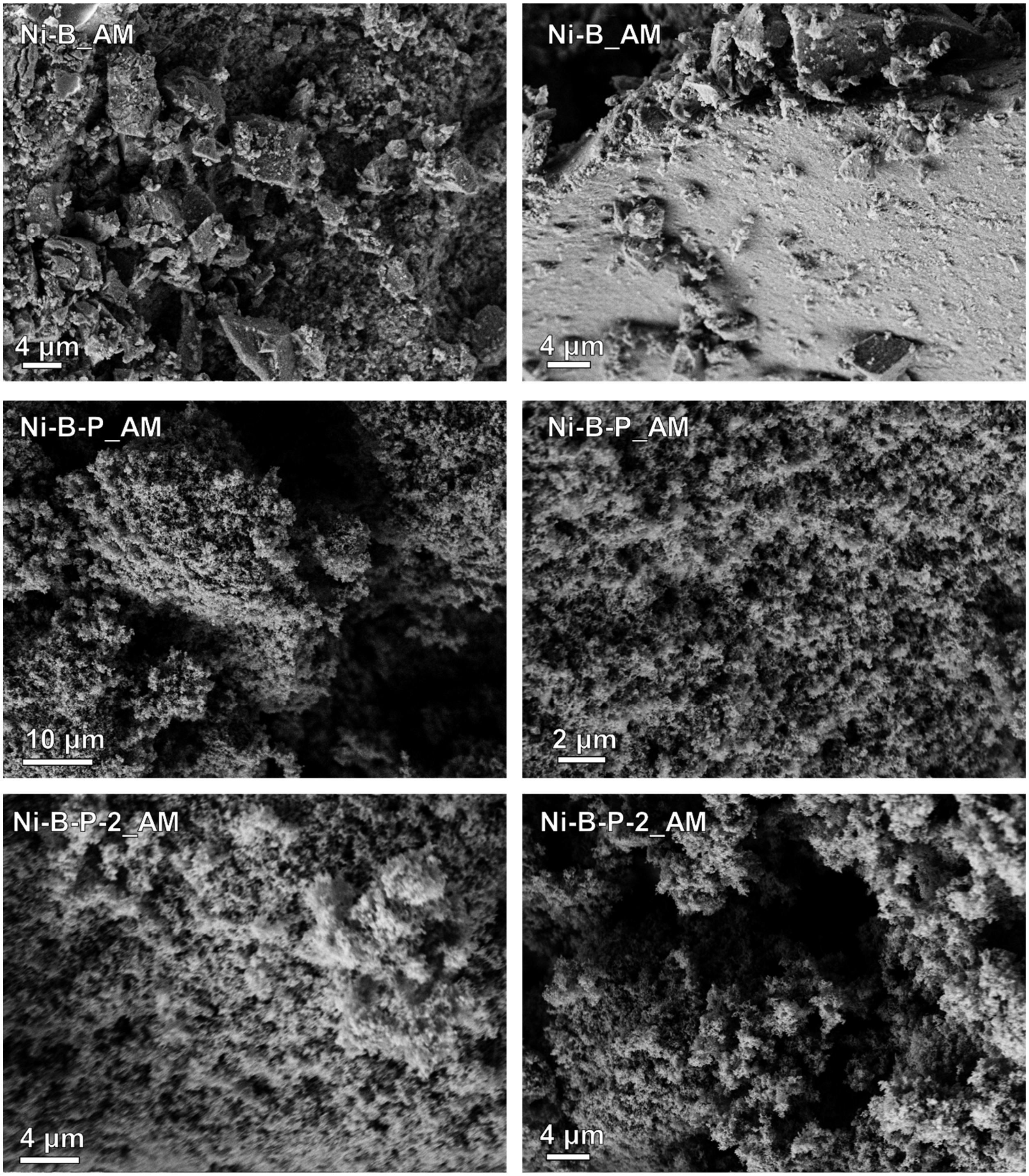

Figure S11. SEM images of Ni-B_AM, Ni-B-P_AM, Ni-B-P-2_AM.

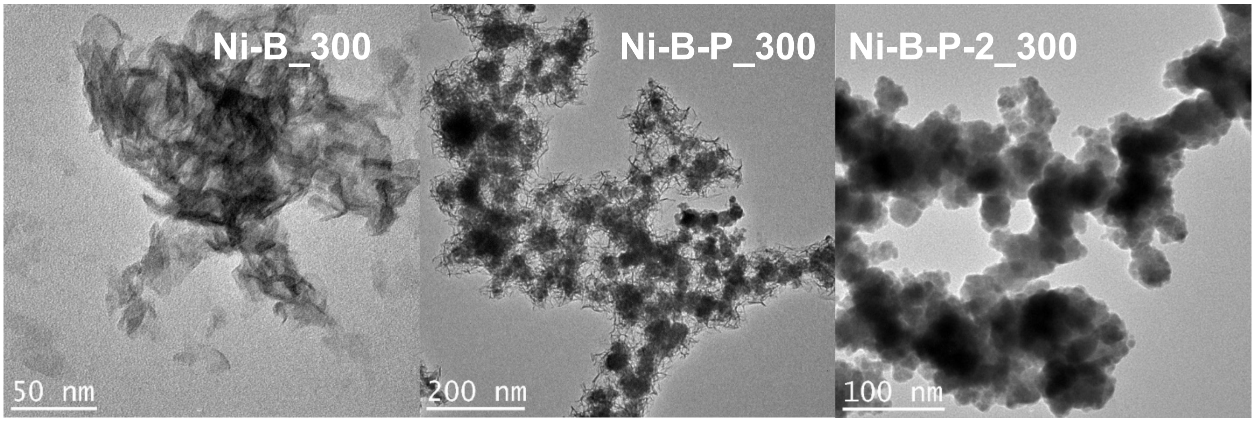

Figure S12. Bright-field TEM images of Ni-B_300, Ni-B-P_300, and Ni-B-P-2_300

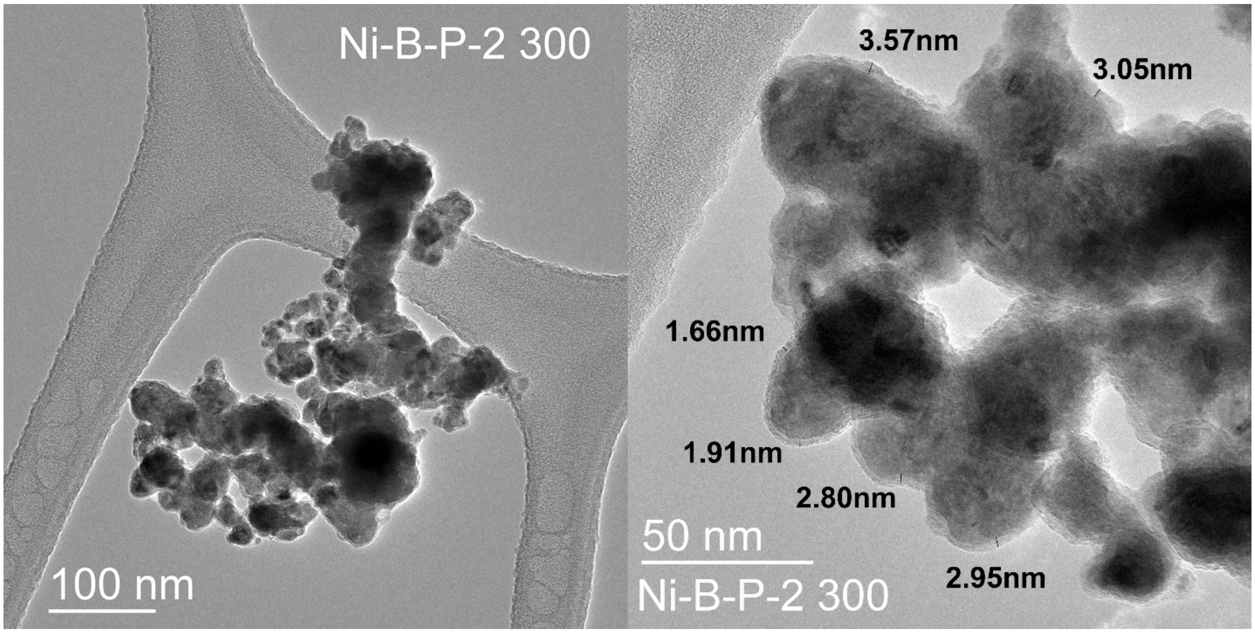

Figure S13. Bright-field TEM images displaying the homogenous shells observed for the Ni-B-P-2_300 core-shell nanoparticles.

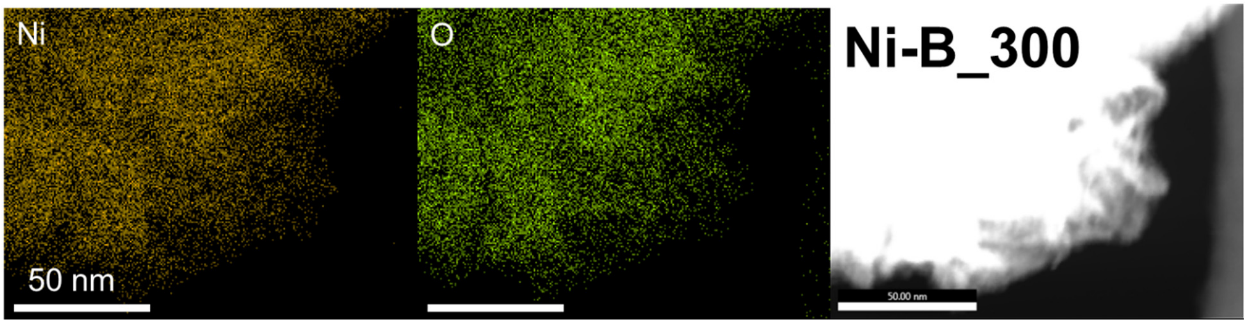

Figure S14. STEM-EDX elemental mapping of Ni-B_300.

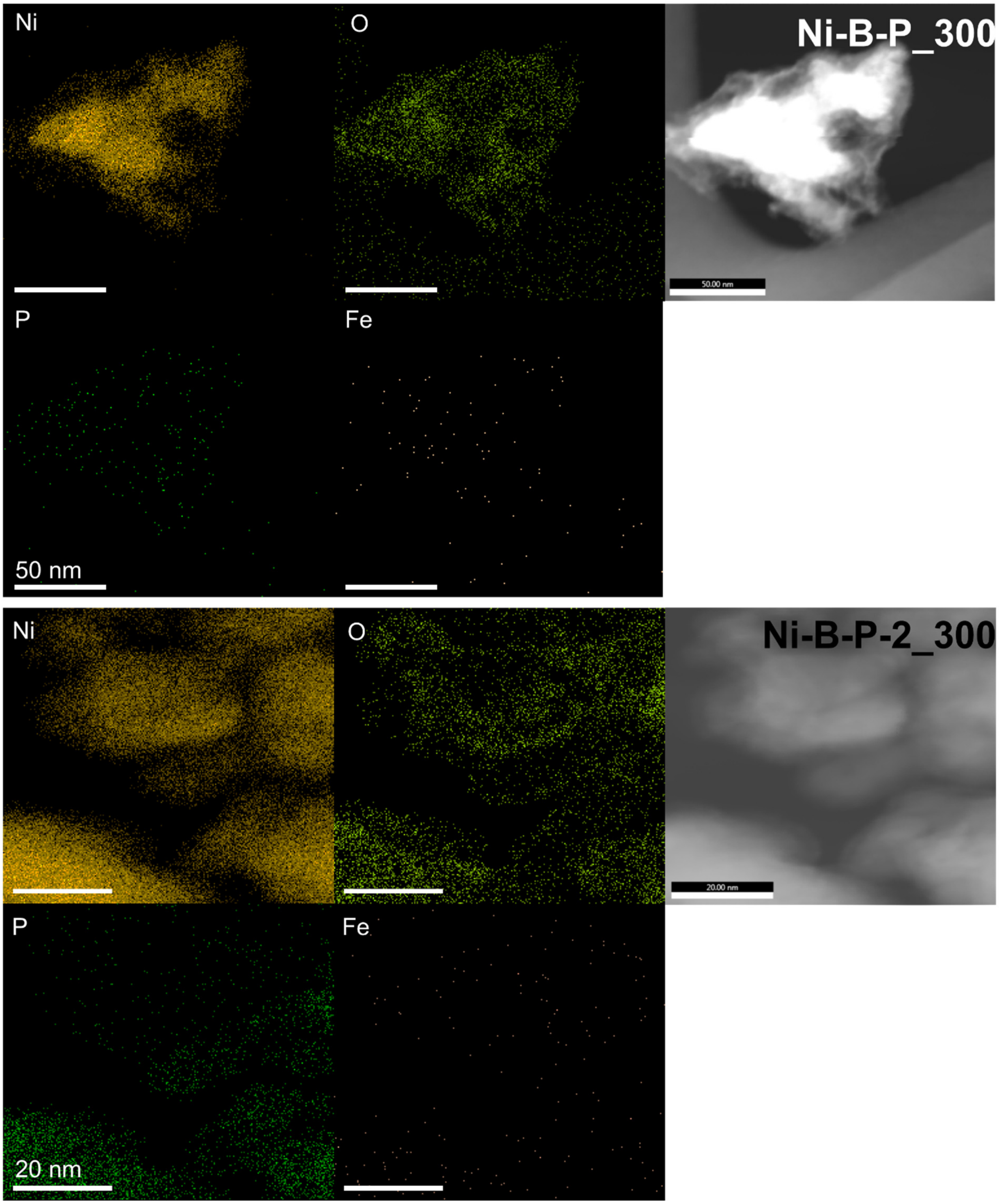

Figure S15. STEM-EDX elemental mapping of Ni-B-P_300 and Ni-B-P-2_300.

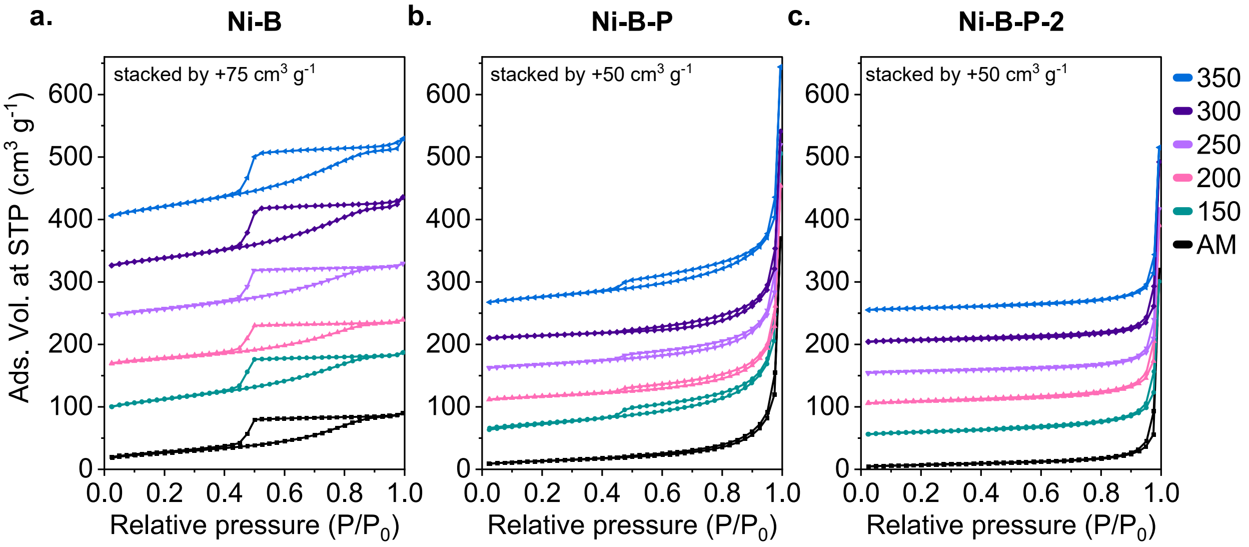

Figure S16. N_2 physisorption isotherms depicting a) Ni-B, b) Ni-B-P, and c) Ni-B-P-2 samples annealed at the indicated temperatures. The measurements were conducted at –196 °C.

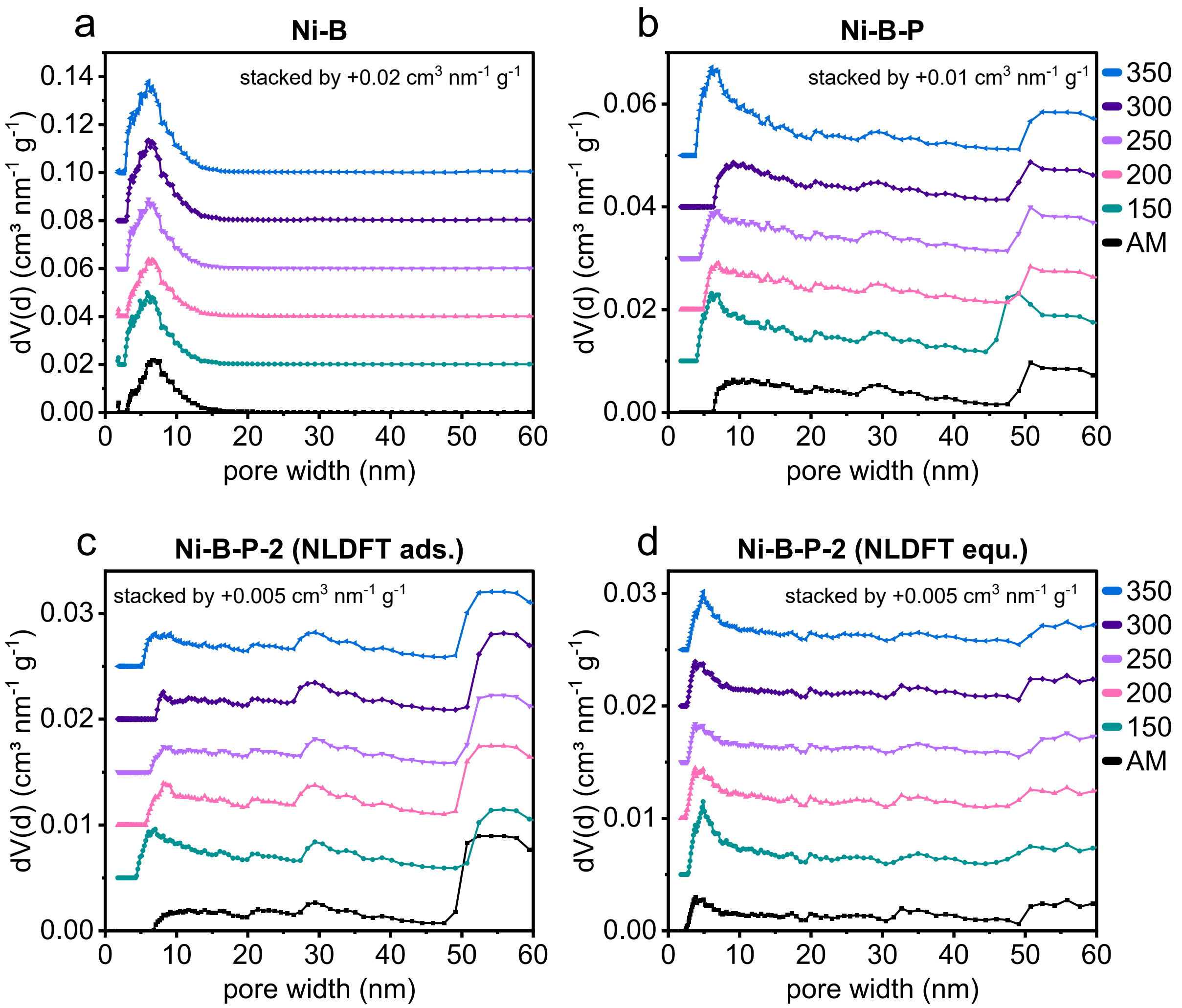

Figure S17. NLDFT PSD calculated using the adsorption branch of the isotherm of a) Ni-B, b) Ni-B-P, and c) Ni-B-P-2 samples annealed at the indicated temperatures. Further, d) depicts the PSD for Ni-B-P-2 samples evaluated with the NLDFT kernel for the desorption branch of the isotherm.

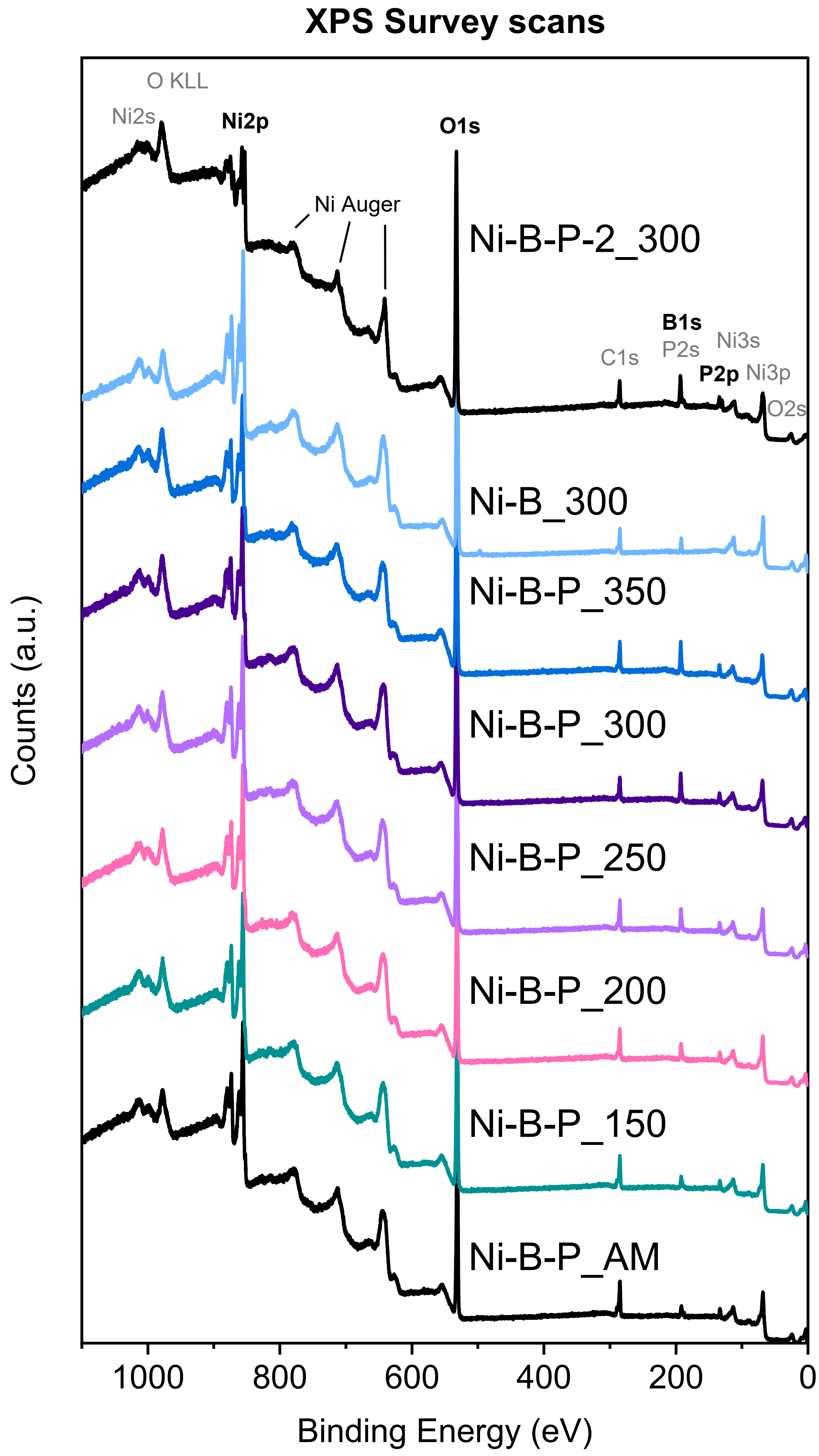

Figure S18. XPS Survey scans of Ni-B-P as-made, and annealed at 150, 200, 250, 300, and 350 °C, and Ni-B and Ni-B-P-2 annealed at 300 °C. The graphs are displayed in stacked form to improve visibility.

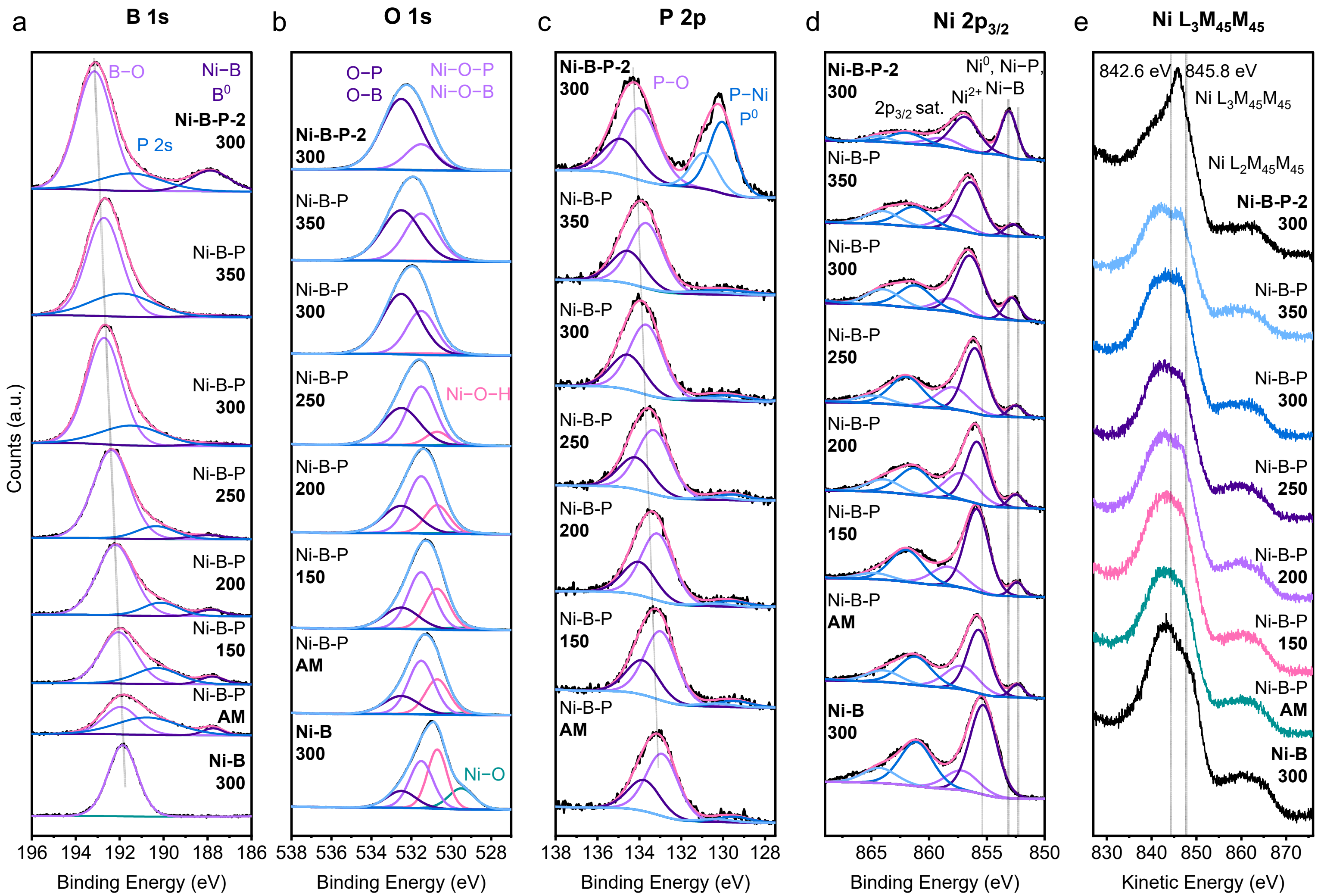

Figure S19. a) B 1s and b) O 1s c) P 2s, d) Ni $2p_{3/2}$, and e) Ni $L_3M_{45}M_{45}$ Auger HR-XPS spectra of Ni-B_300, Ni-B-P as-made, 150, 200, 250, 300, and 350, and Ni-B-P-2_300. The graphs are displayed in stacked form to improve visibility.

.

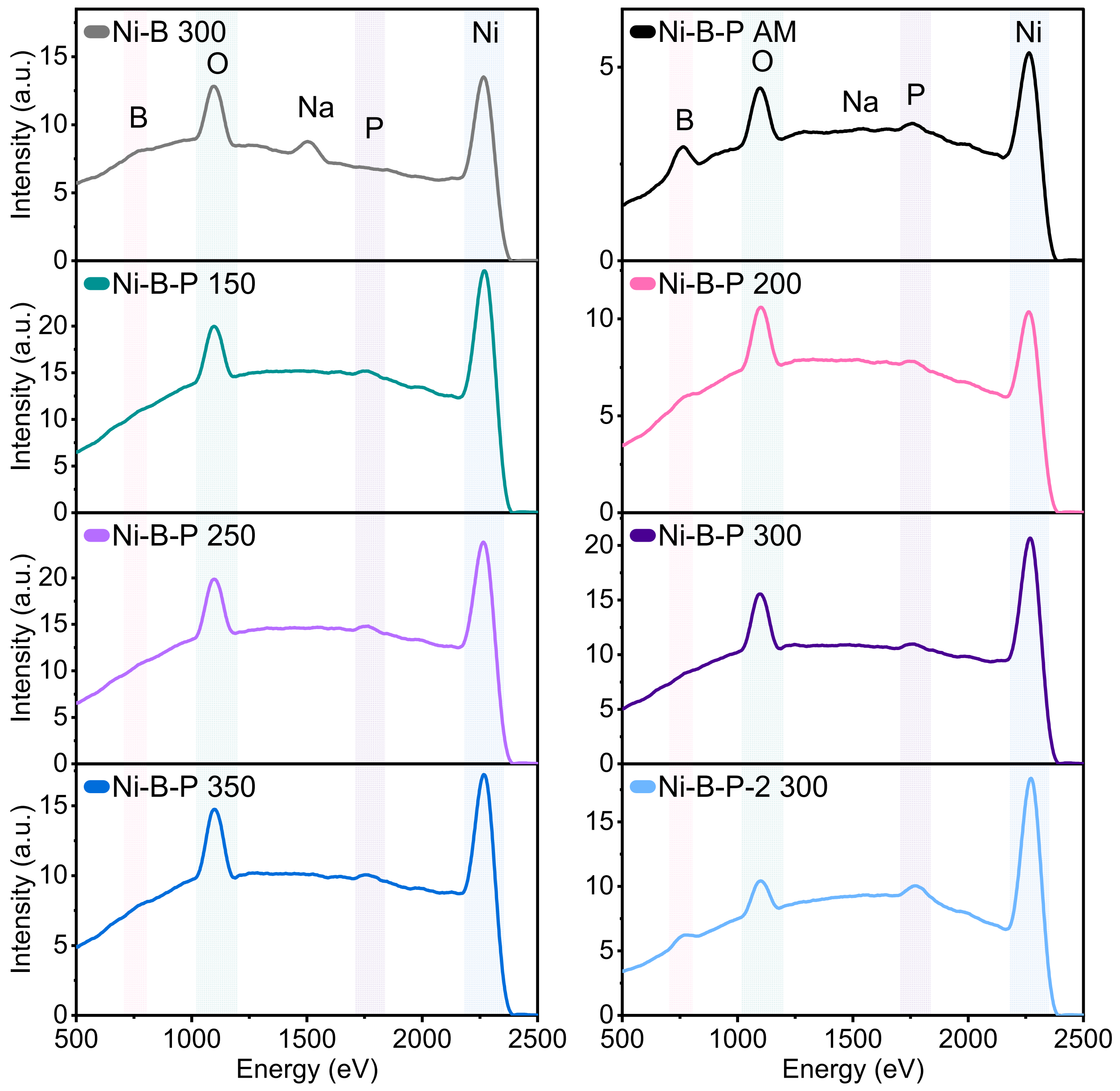

Figure S20. Low energy ion scattering (LEIS) spectroscopy surface characterization of Ni-B_300, Ni-B-P_AM, 150, 200, 250, 300, 350, and Ni-B-P-2_300 recorded with He ions accelerated to 3keV.

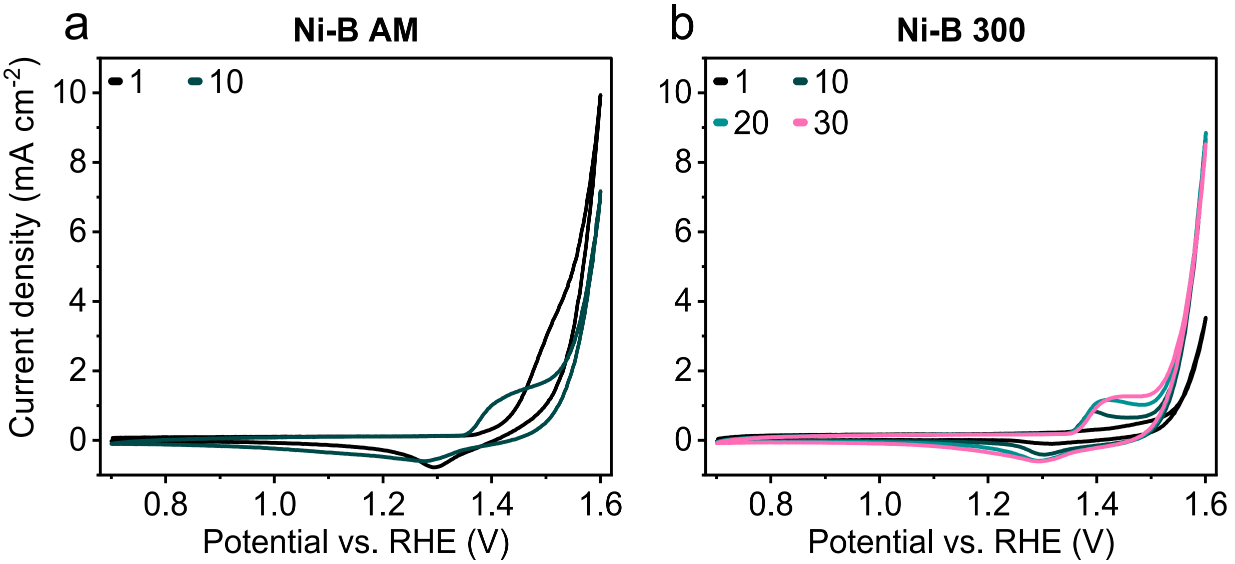

Figure S21. CV scans obtained for a) Ni-B_AM and b) Ni-B_300 during the initial activation protocol. The cycle numbers of the depicted scans are displayed in the top left corner of the graphs respectively.

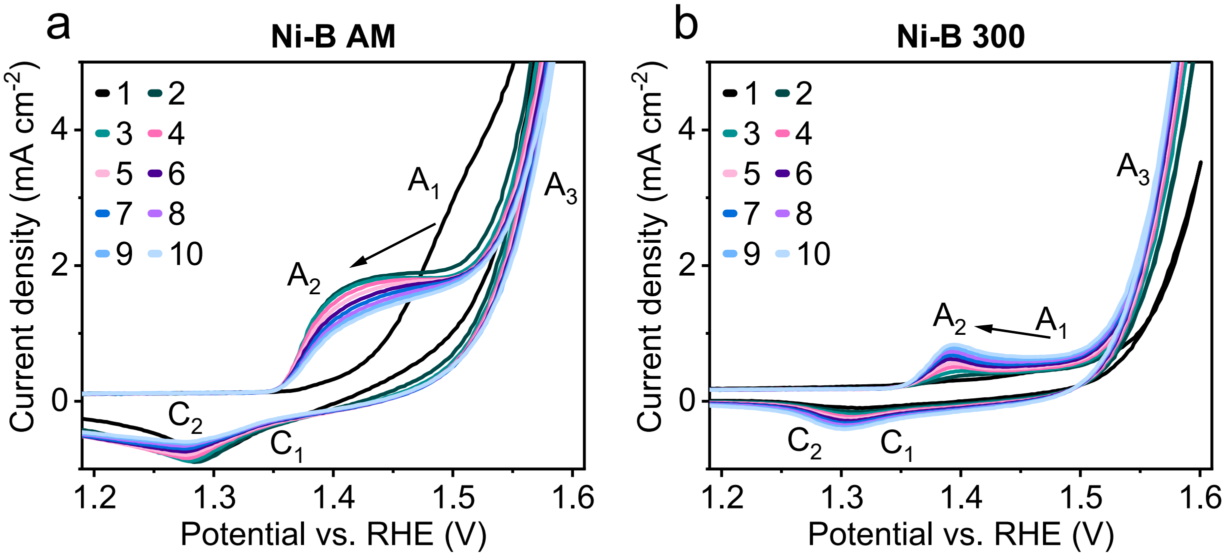

Figure S22. Zoomed-in view of the first 10 CV curves of a) Ni-B_AM and b) Ni-B_300. The arrows highlight redox changes in the CV curves during the activation protocol.

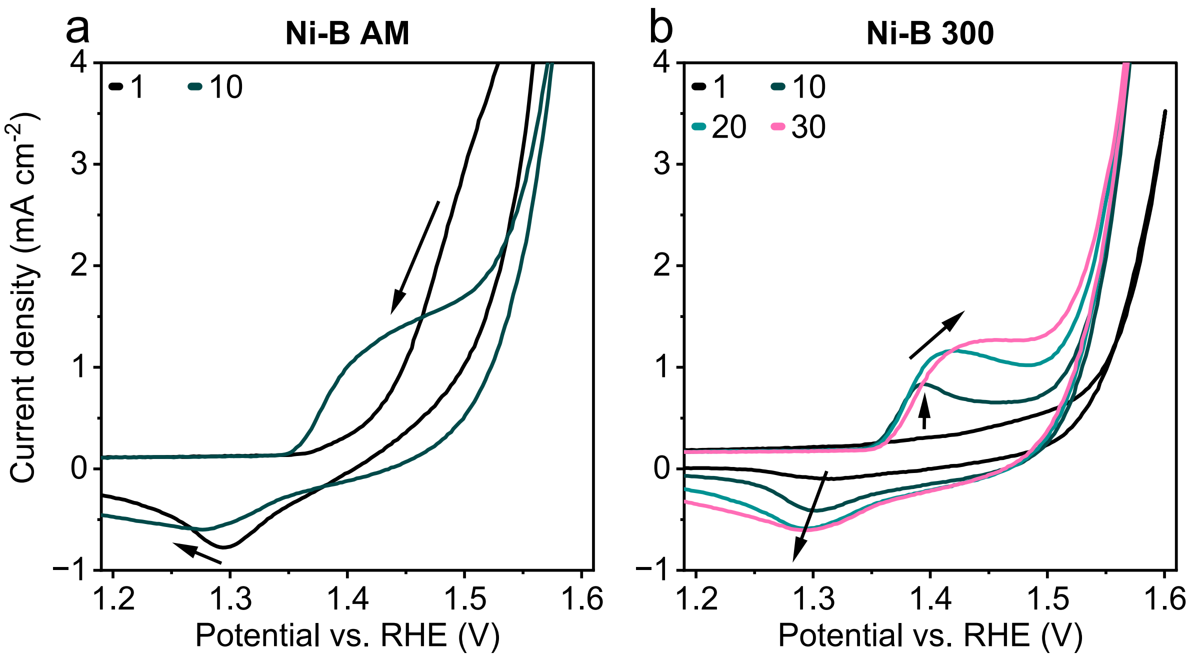

Figure S23. Zoomed-in view of the CV curves of a) Ni-B_AM and b) Ni-B_300 recorded in 1 M KOH with a sweep rate of 50 mV s^{-1}. The arrows highlight changes in the CV curves during the activation protocol.

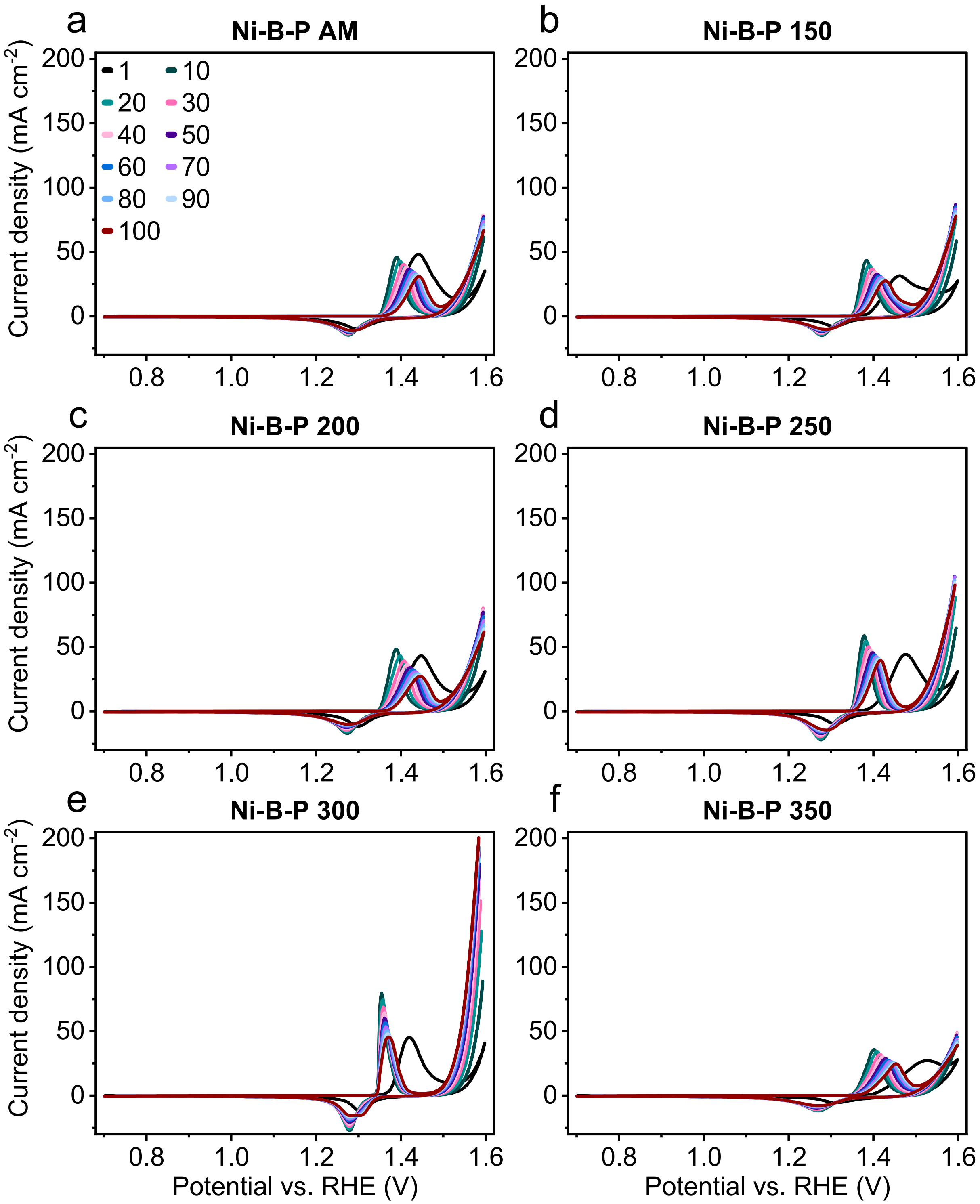

Figure S24. CV curves of a) Ni-B-P_AM, b) 150, c) 200, d) 250, e) 300, and f) 350 recorded in 1 M KOH with a sweep rate of 50 mV s^{-1}. The cycle numbers of the depicted scans are displayed in the top left corner of the a) Ni-B-P_AM graph.

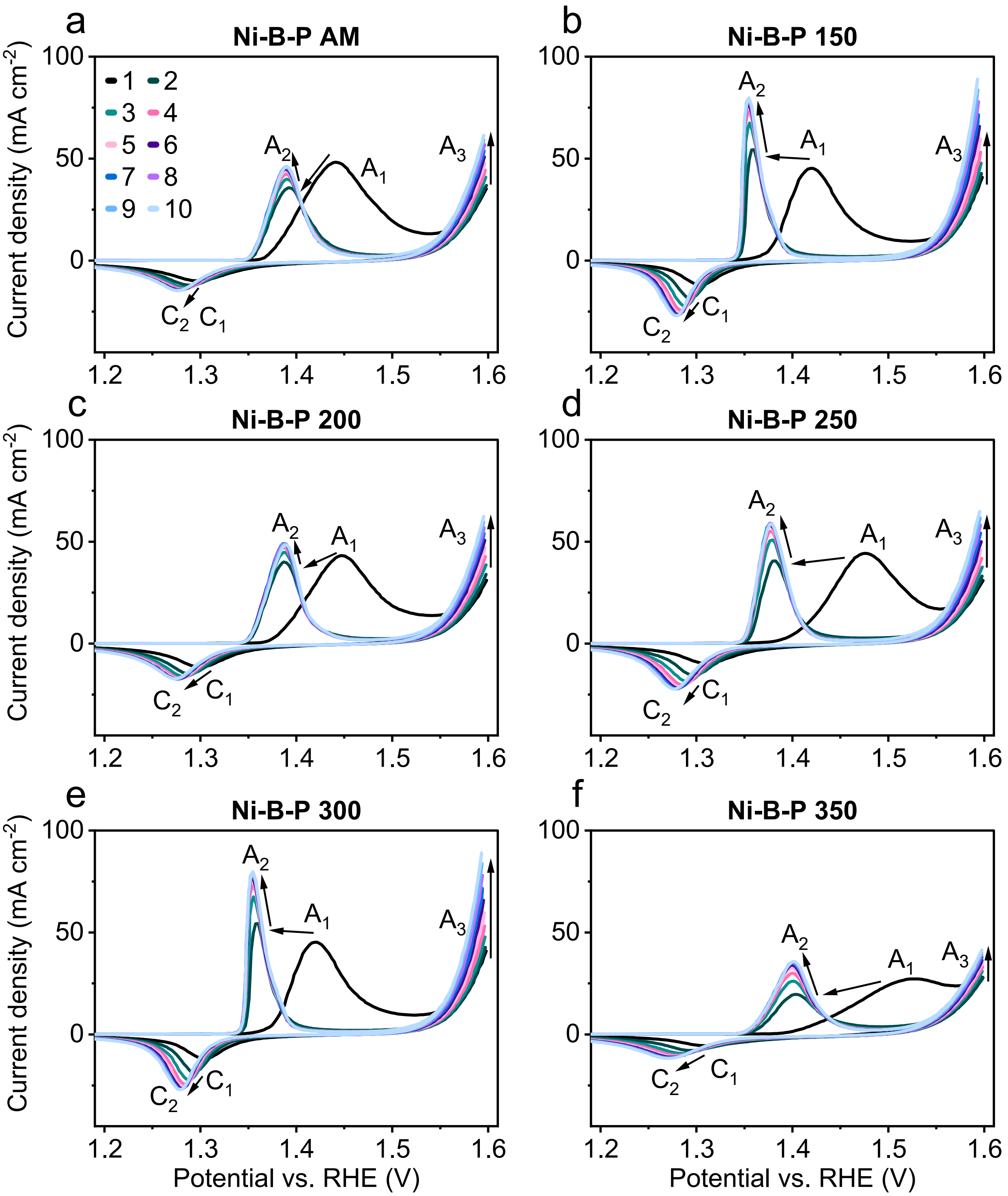

Figure S25. Zoomed-in view of the first ten CV curves of a) Ni-B-P_AM, b) 150, c) 200, d) 250, e) 300, and f) 350 recorded in 1 M KOH with a sweep rate of 50 mV s^{-1}. The arrows highlight changes in the CV curves during the activation protocol.

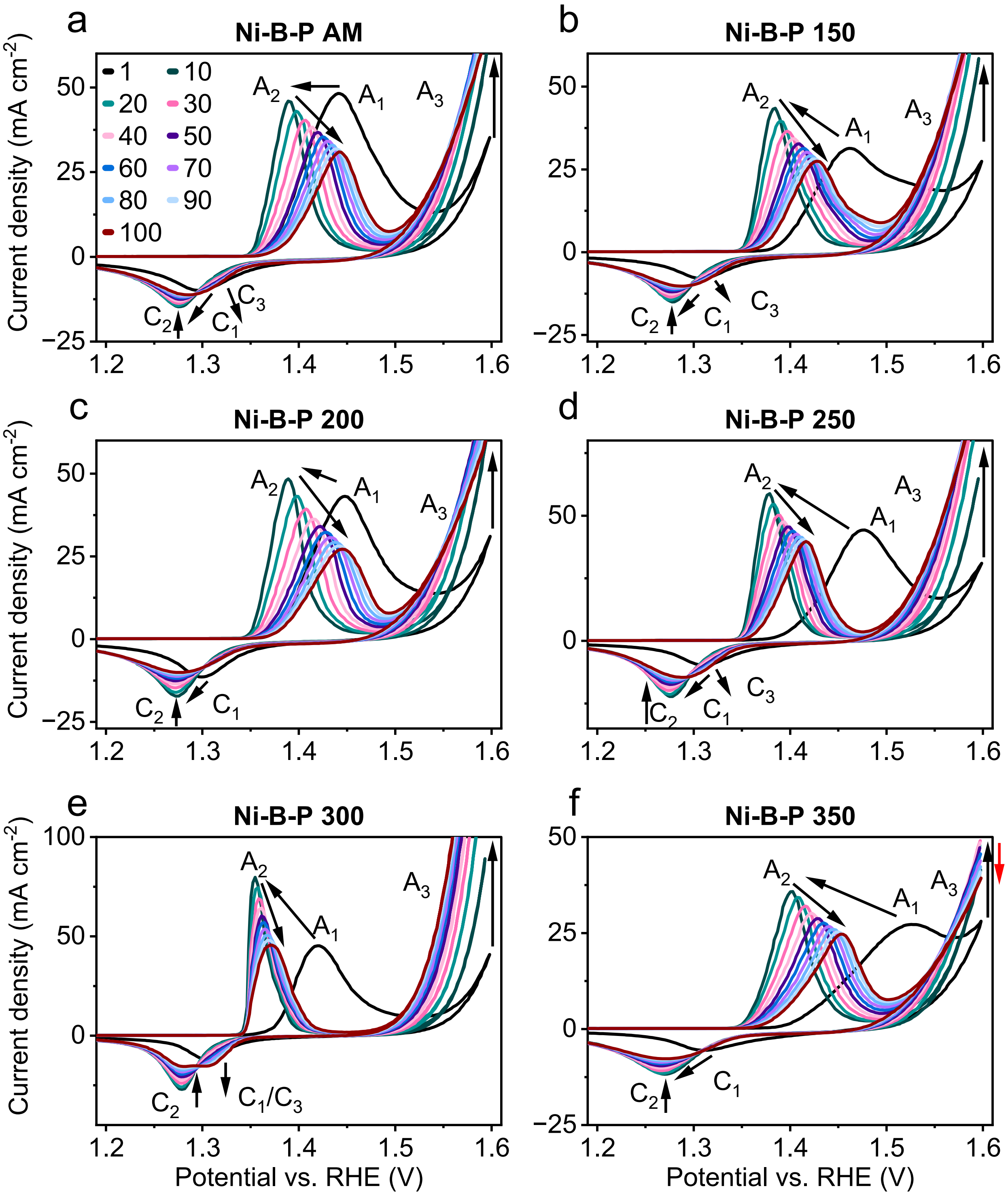

Figure S26. Zoomed-in view of the CV curves of a) Ni-B-P_AM, b) 150, c) 200, d) 250, e) 300, and f) 350 recorded in 1 M KOH with a sweep rate of 50 mV s^{-1}. The arrows highlight changes in the CV curves during the activation protocol.

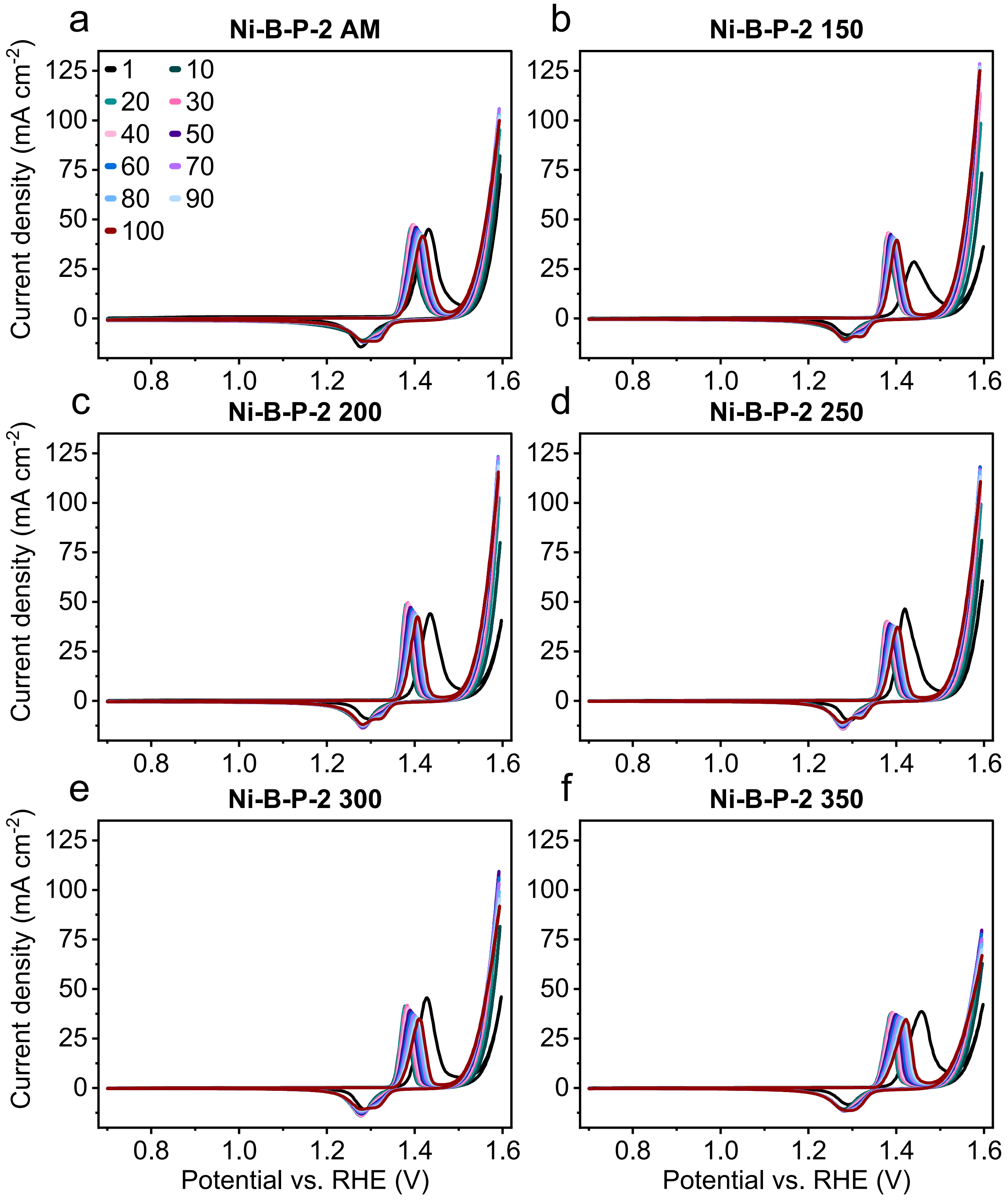

Figure S27. CV curves of a) Ni-B-P-2_AM, b) 150, c) 200, d) 250, e) 300, and f) 350 recorded in 1 M KOH with a sweep rate of 50 mV s^{-1}. The cycle numbers of the depicted scans are displayed in the top left corner of the a) Ni-B-P-2_AM graph.

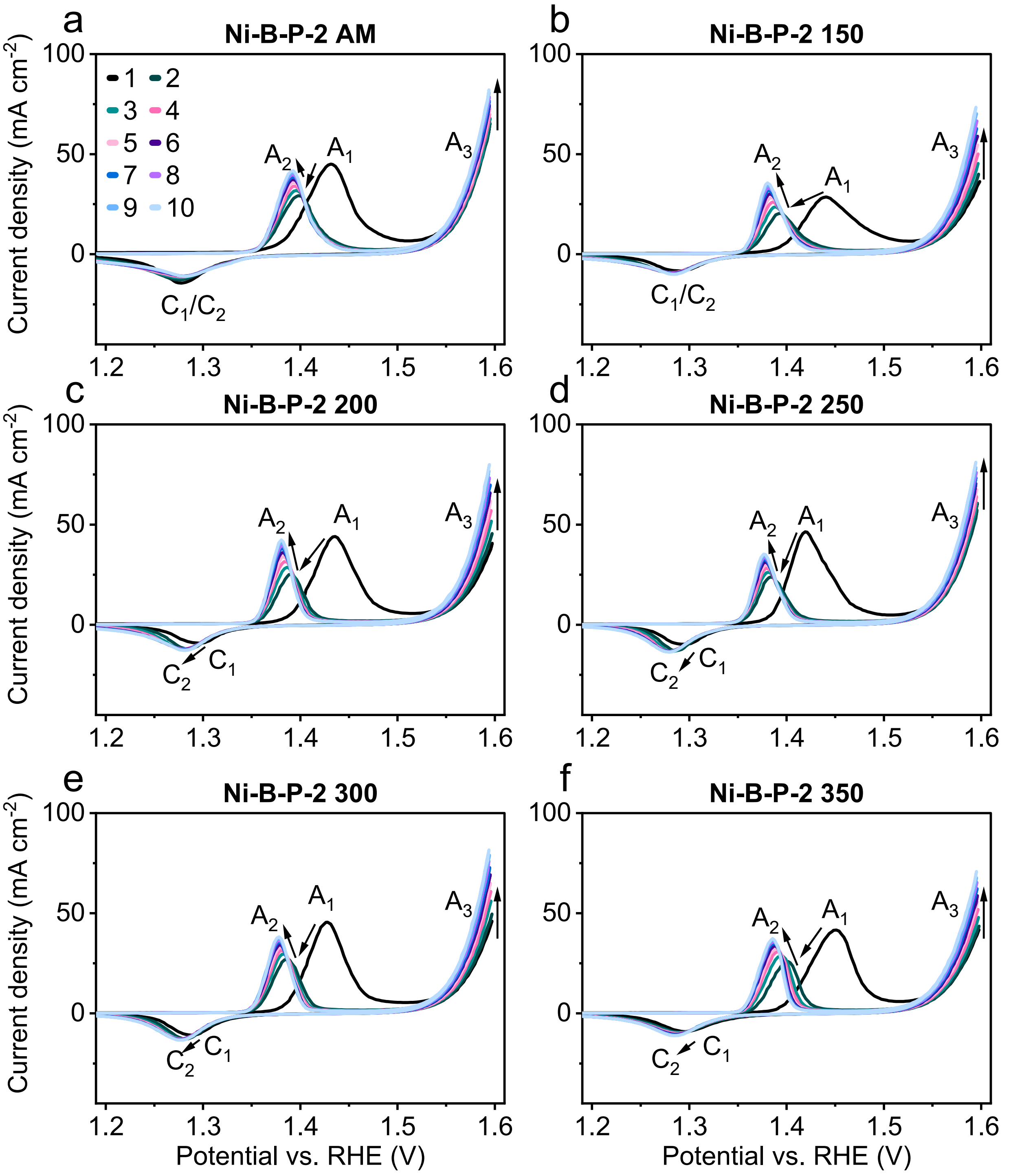

Figure S28. Zoomed-in view of the first ten CV curves of a) Ni-B-P_AM, b) 150, c) 200, d) 250, e) 300, and f) 350 recorded in 1 M KOH with a sweep rate of 50 mV s^{-1}. The arrows highlight changes in the CV curves during the activation protocol.

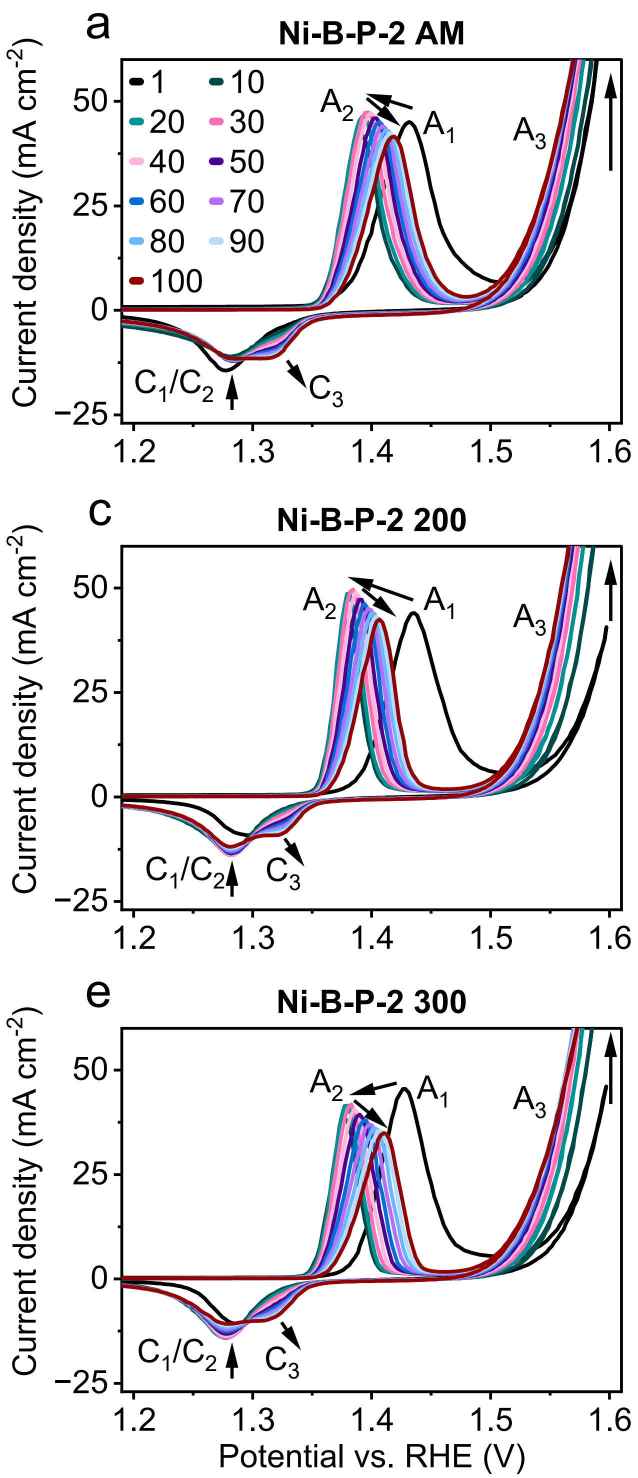

Figure S29. Zoomed-in view of the CV curves of a) Ni-B-P-2_AM, b) 150, c) 200, d) 250, e) 300, and f) 350 recorded in 1 M KOH with a sweep rate of 50 mV s^{-1}. The arrows highlight changes in the CV curves during the activation protocol.

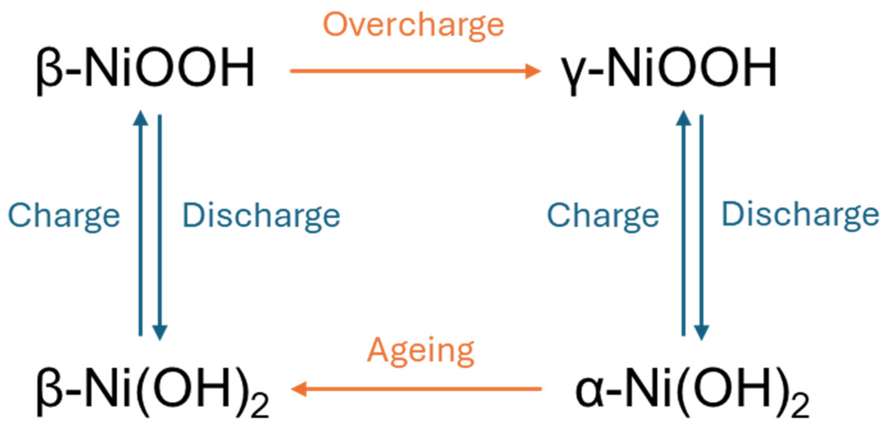

Figure S30. Bode Scheme displaying the typical reactions nickel hydroxide redox switching reactions upon electrochemical cycling in alkaline environments. Adapted from Bode et al.[8]

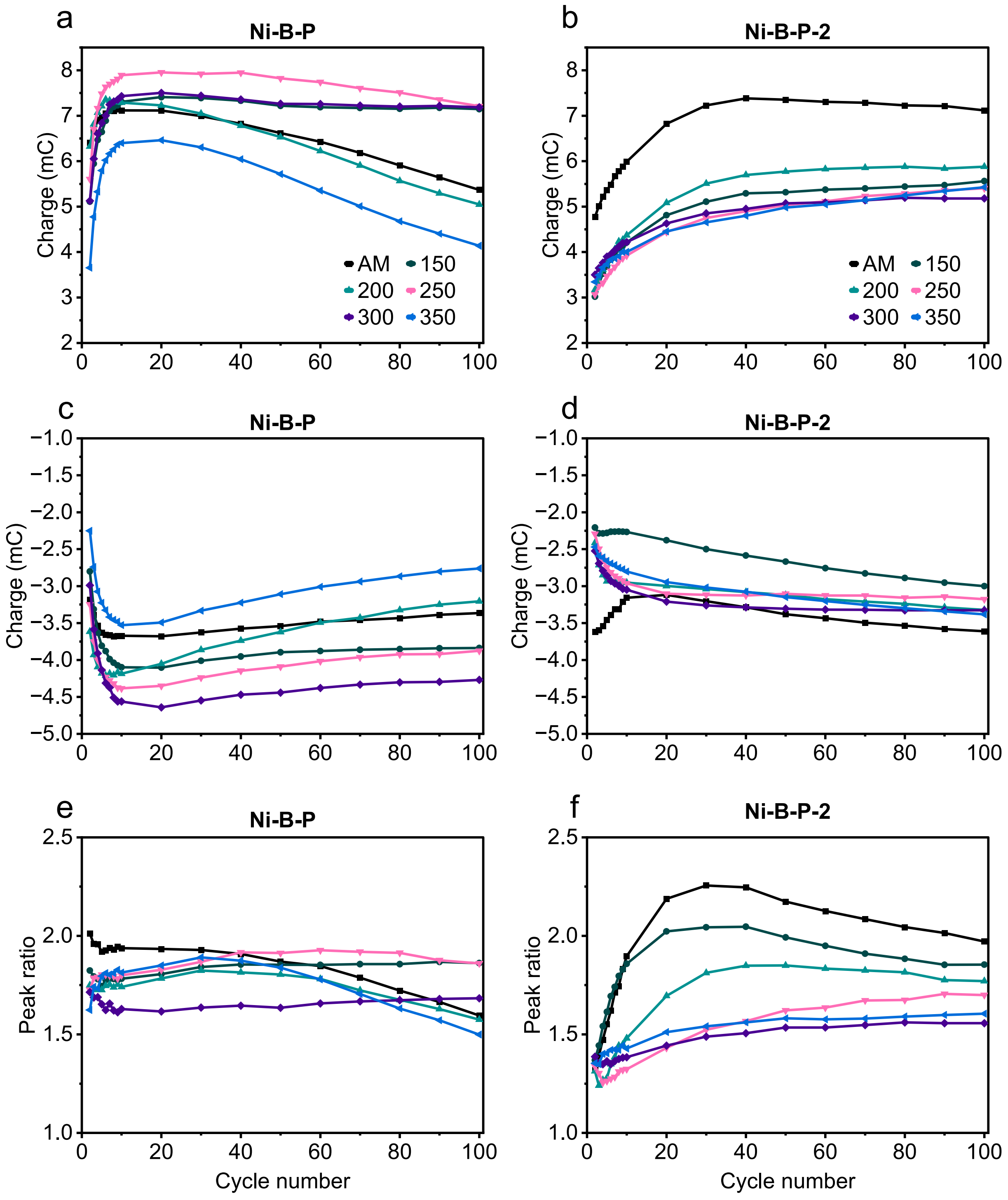

Figure S31. a) and b) display the electrical charge consumed by the precatalytic oxidation (A_2) in the forward CV scan and c) and d) the reduction (C_2+C_3) in the reverse scan as a function of the CV cycle number. The A_2:(C_2+C_3) charge capacity ratios are displayed in e) and f) for the Ni-B-P and Ni-B-P-2 samples respectively.

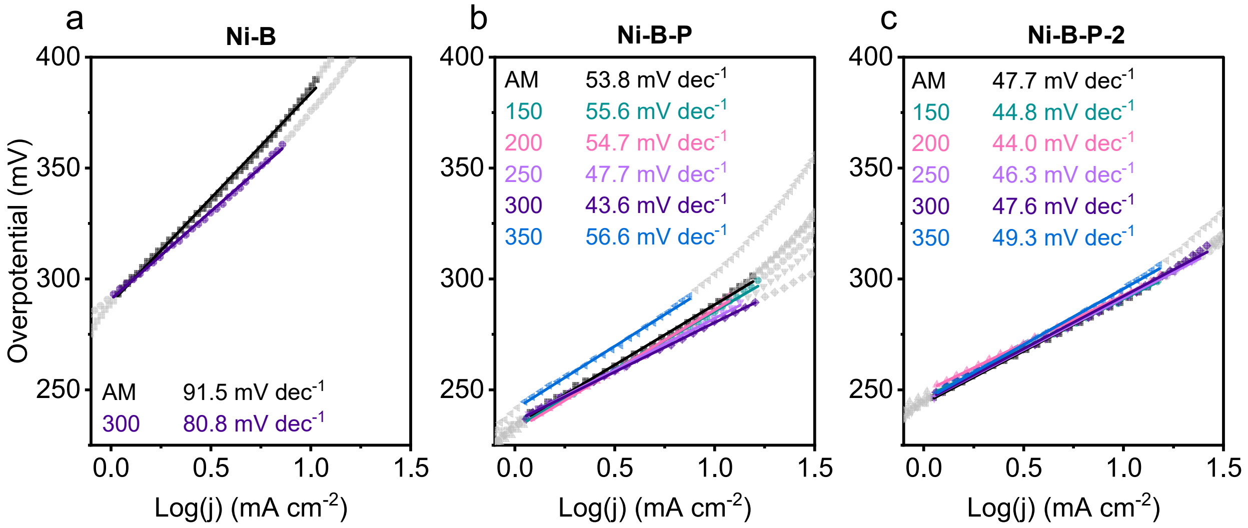

Figure S32. Tafel plots comparing a) Ni-B, b) Ni-B-P, and c) Ni-B-P-2 annealed at the indicated temperatures.

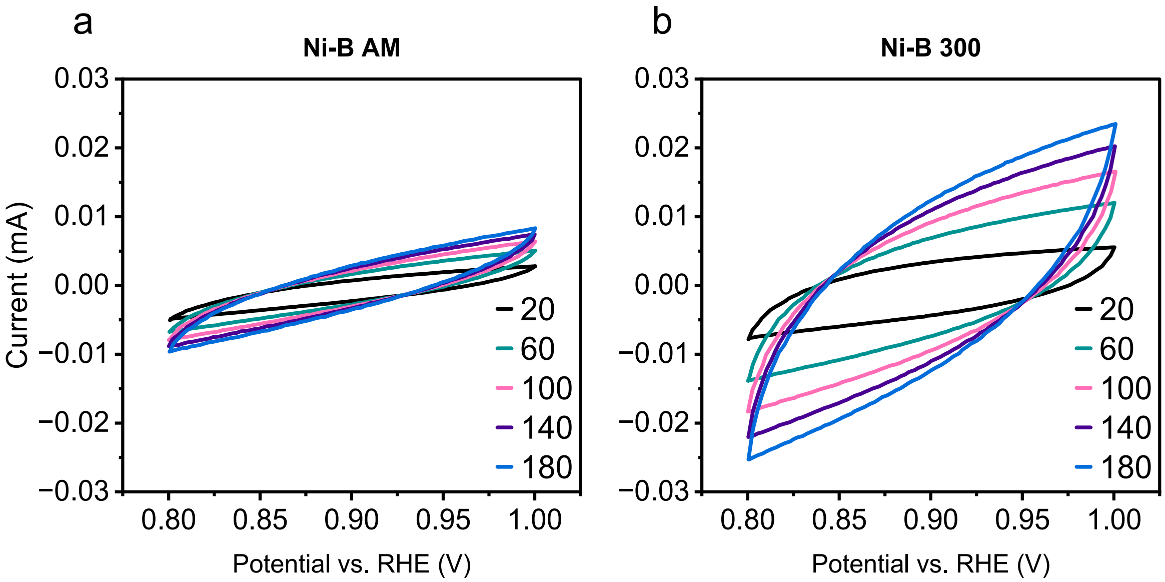

Figure S33. CV measurements of a) Ni-B AM and b) NiB 300 performed at sweep rates between 20 – 180 mV s^{-1} for electrochemical capacitance (C_{dl}) determination.

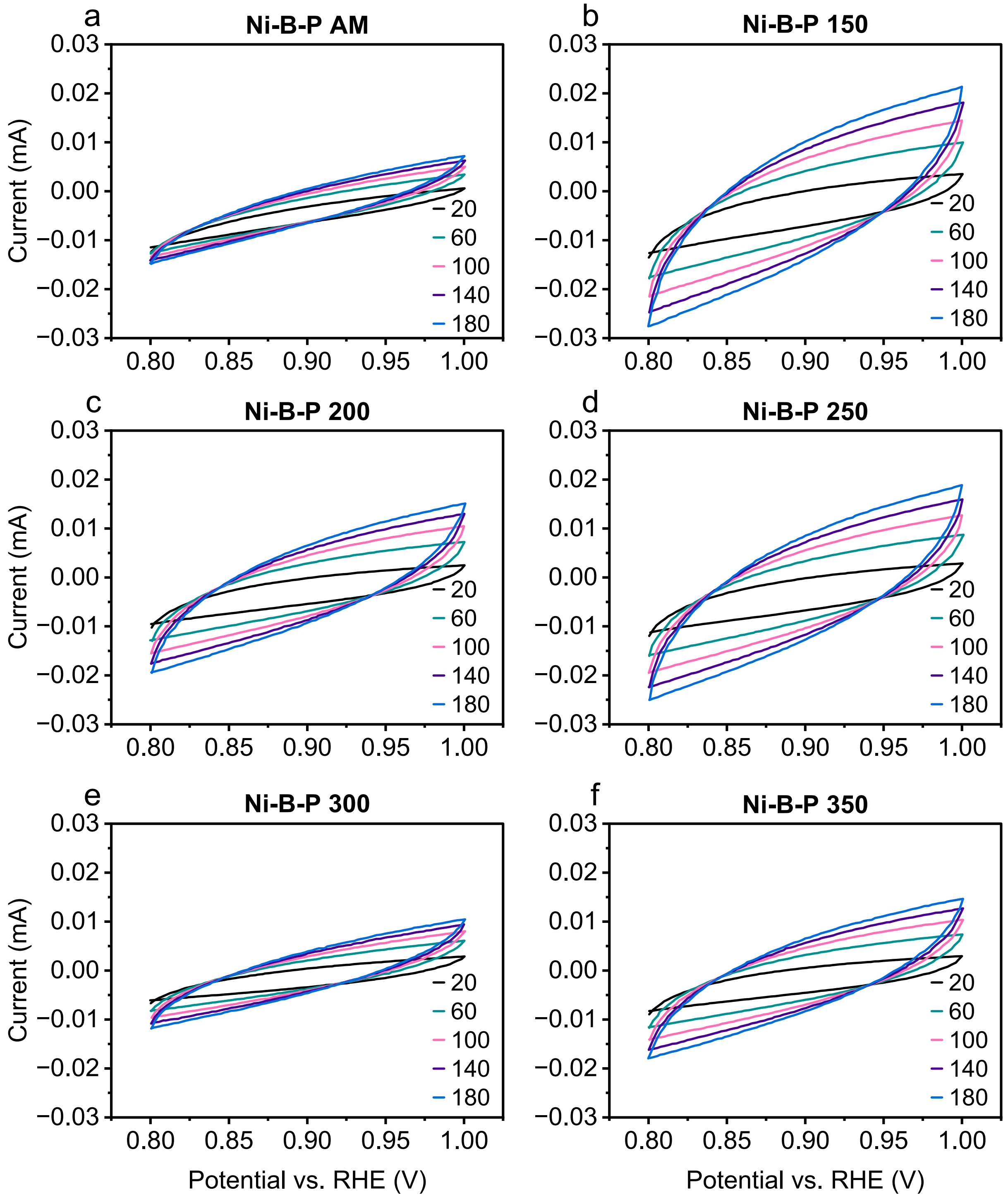

Figure S34. CV measurements of a) Ni-B-P AM, b) Ni-B-P 150, c) Ni-B-P 200, d) Ni-B-P 250, e) Ni-B-P 300, and f) Ni-B-P 350 performed at sweep rates between 20 – 180 mV s^{-1} for electrochemical capacitance (C_{dl}) determination.

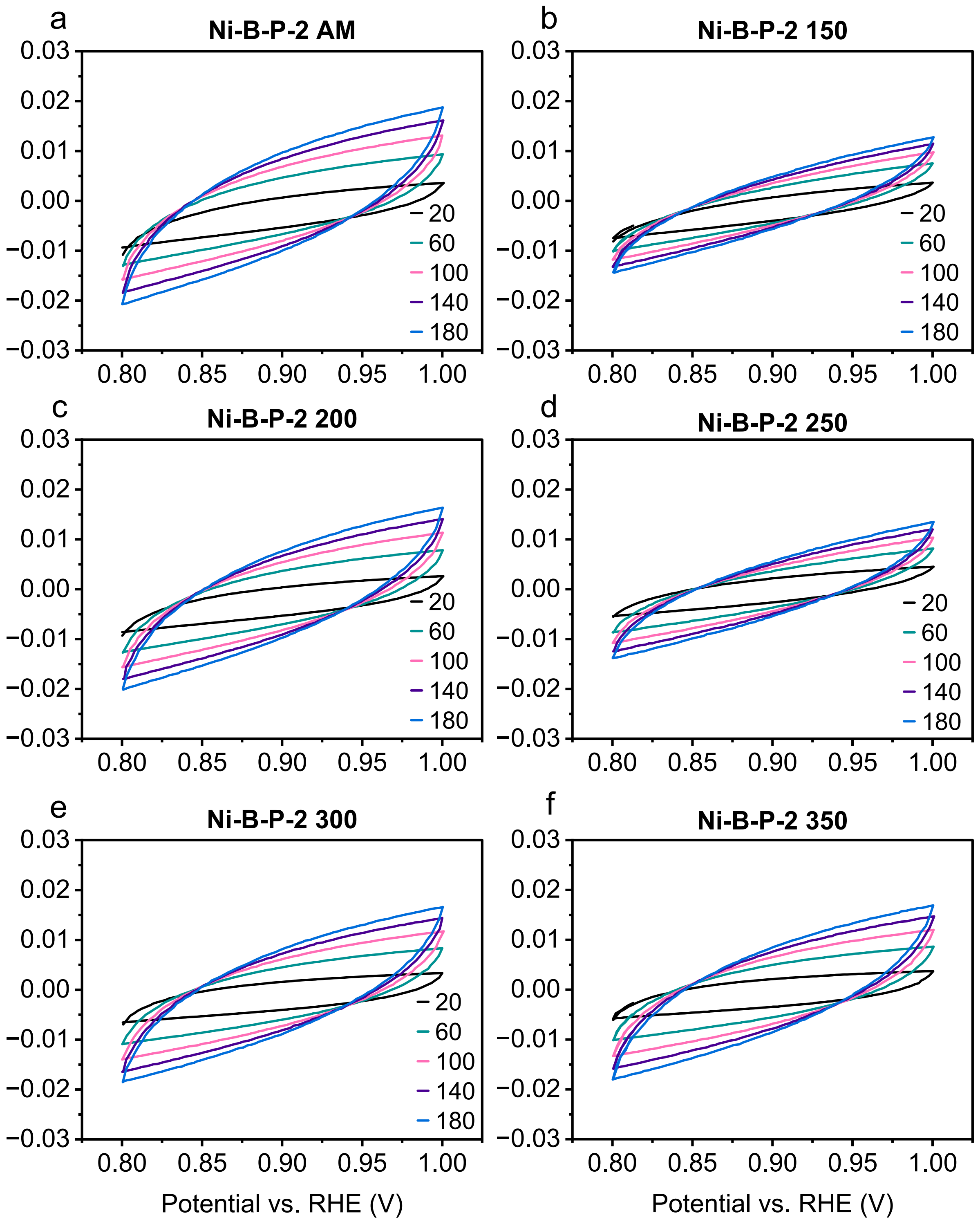

Figure S35. CV measurements of a) Ni-B-P-2 AM, b) Ni-B-P-2 150, c) Ni-B-P-2 200, d) Ni-B-P-2 250, e) Ni-B-P-2 300, and f) Ni-B-P-2 350 performed at sweep rates 20 – 180 mV s^{-1} for electrochemical capacitance (C_{dl}) determination.

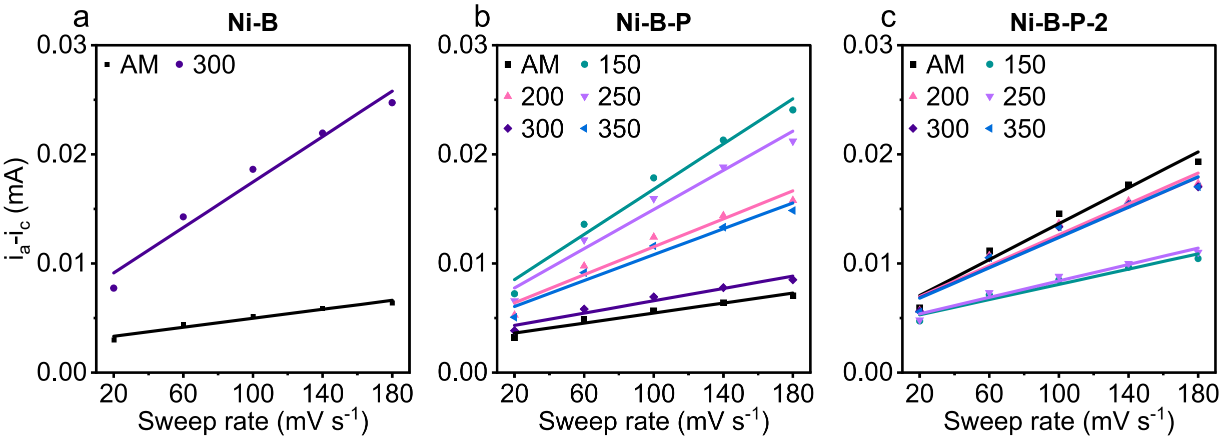

Figure S36. Capacitive current difference (anodic – cathodic) at 0.9 V vs. RHE against applied scan rate for a) Ni-B, b) Ni-B-P, and c) Ni-B-P-2 samples.

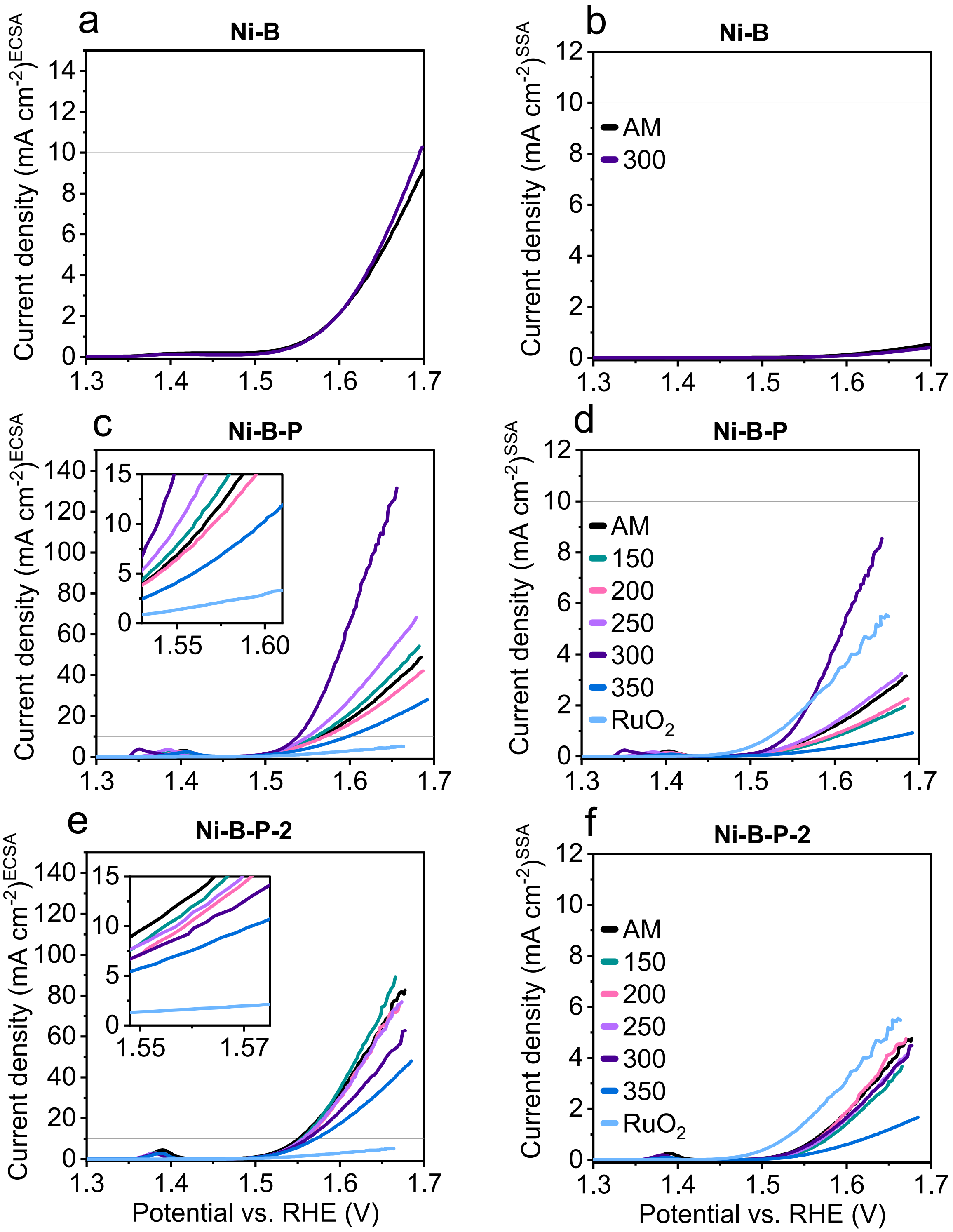

Figure S37. OER LSV curves normalized by ECSA a) Ni-B, c) Ni-B-P, and e) Ni-B-P-2, and BET SSA b) Ni-B, d) Ni-B-P, and f) Ni-B-P-2.

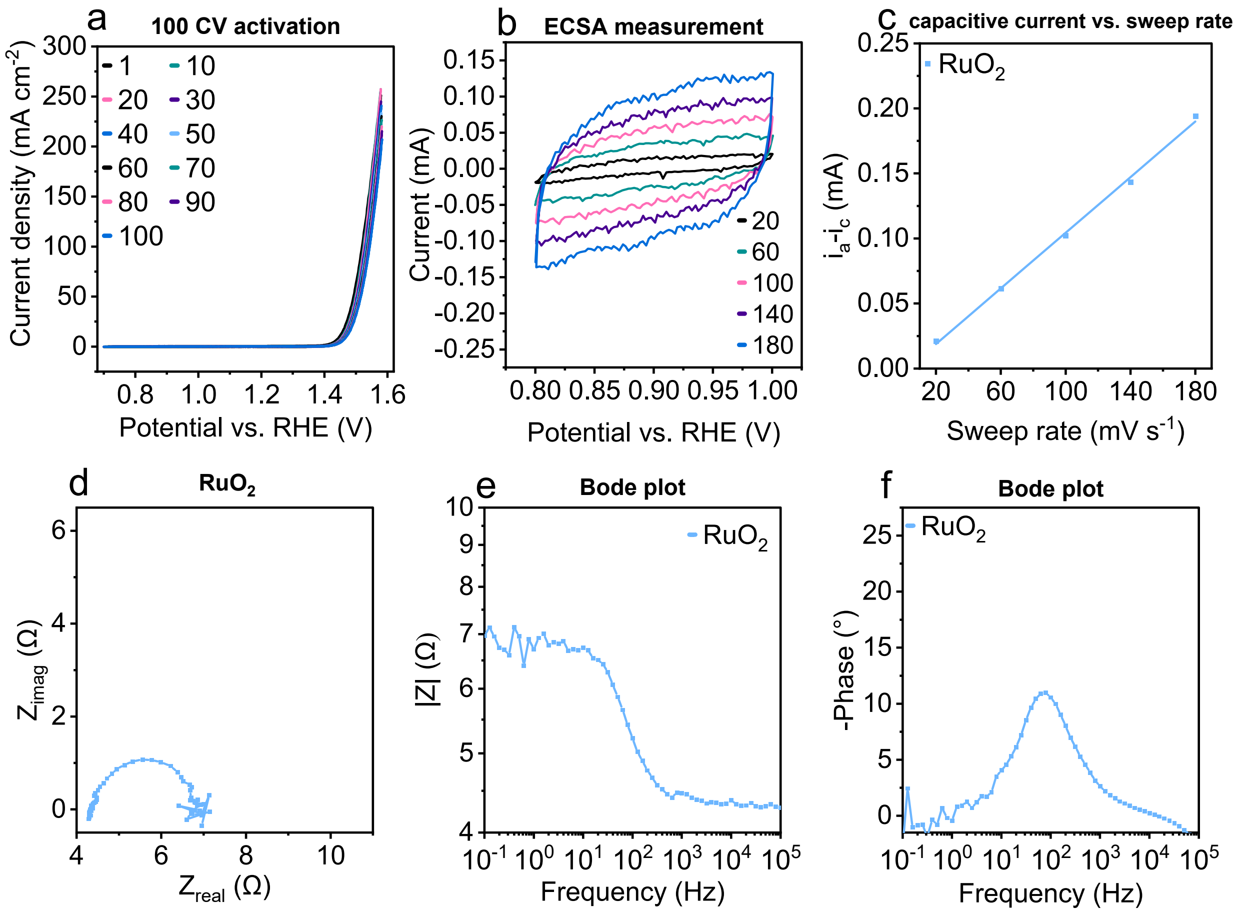

Figure S38. Commercial RuO_2 reference material: a) 100 CV activation, b) CV measurement for ECSA determination, c) capacitive current vs. sweep rate, d) Nyquist plot, e) and f) Bode plots.

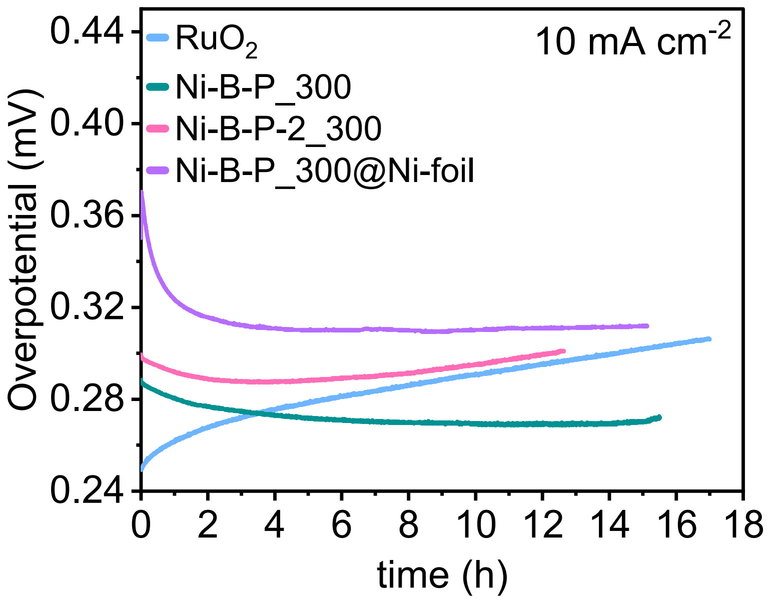

Figure S39. Chronopotentiometric stability test for RuO_2 reference sample and Ni-B-P_300, Ni-B-P-2_300, and Ni-B-P_300@Ni-foil after activation and measurements (100 CVs, 3 LSVs, EIS, CVs (EDLC)) employing 10 mA cm^2.

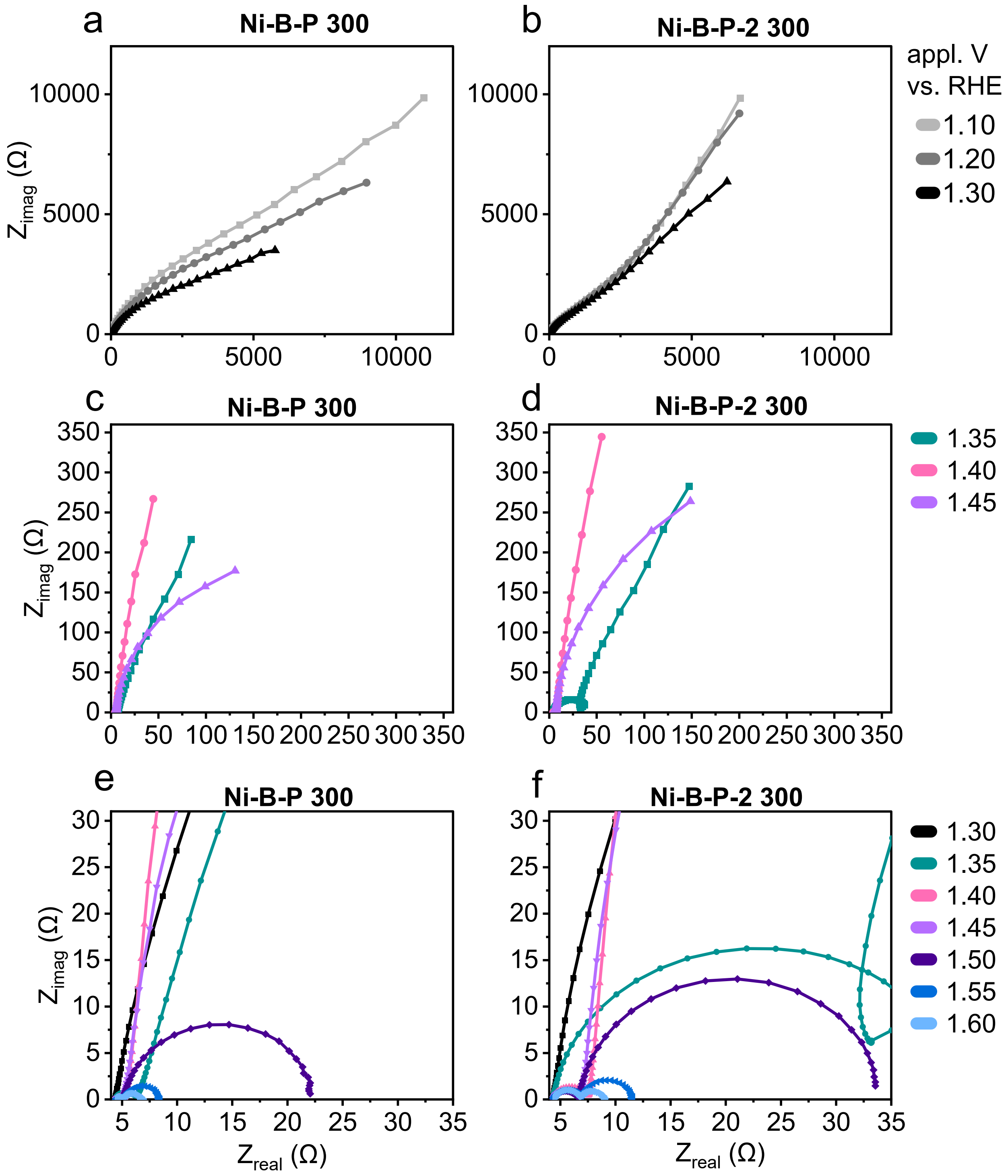

Figure S40. Nyquist plots of Ni-B-P_300 (a, c, and e) and Ni-B-P-2 300 (b, d, and f) obtained from operando EIS measurements conducted at potentials ranging from 1.1 – 1.6 V vs. RHE.

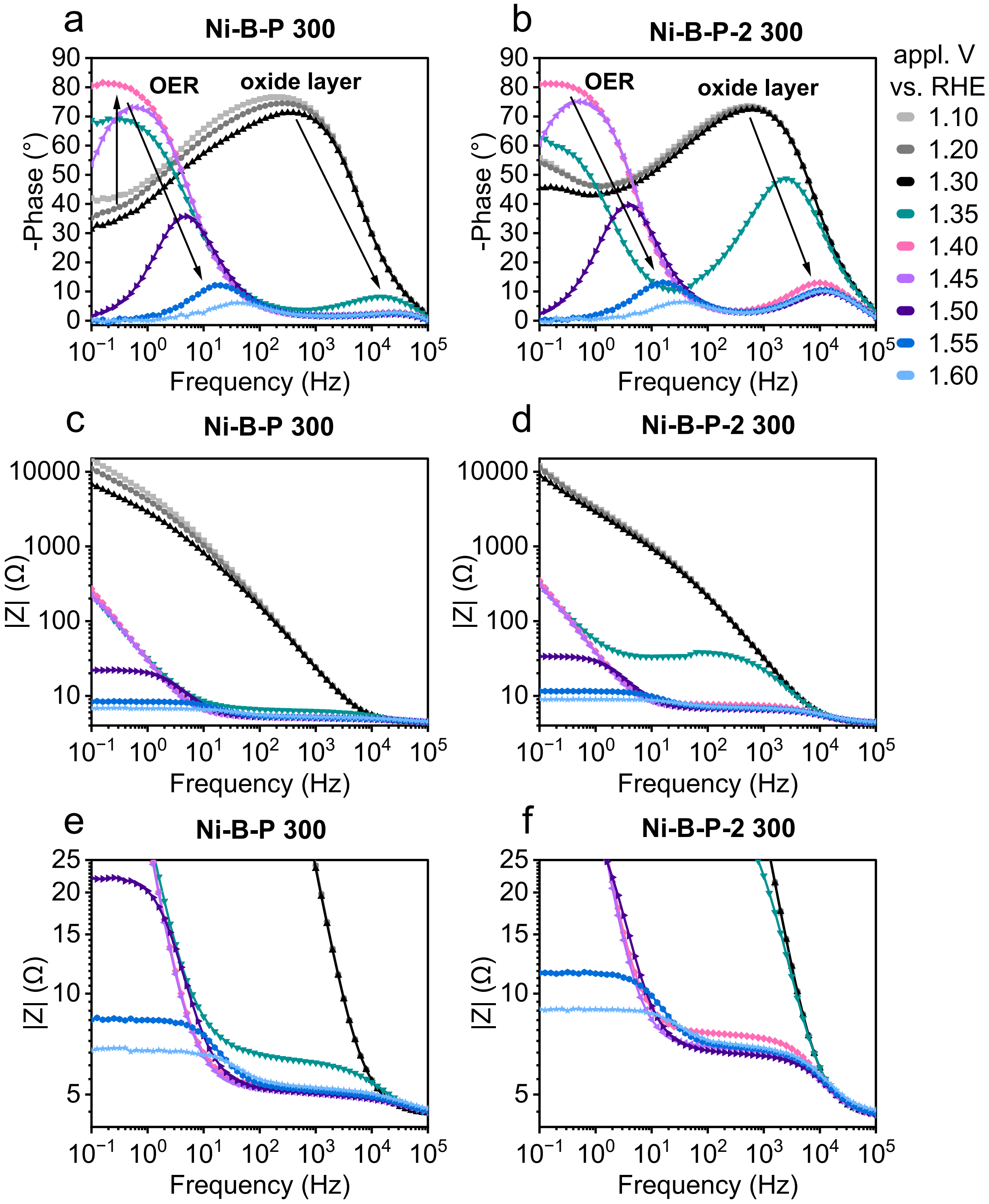

Figure S41. Bode plots of Ni-B-P_300 (a, c, and e) and Ni-B-P-2_300 (b, d, and f) obtained from operando EIS measurements conducted at potentials ranging from 1.1 – 1.6 V vs. RHE.

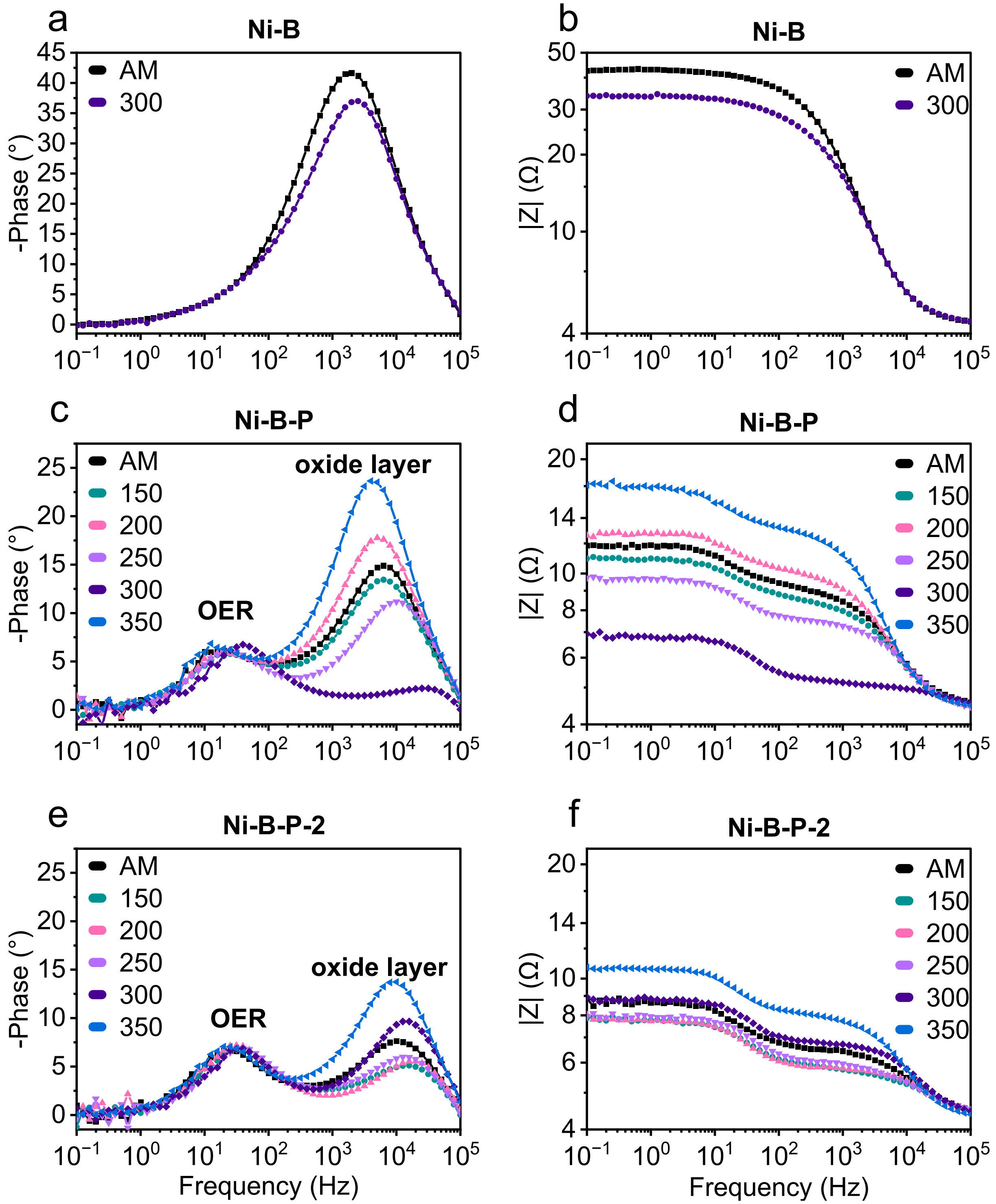

Figure S42. Bode plots of a-b) Ni-B, c-d) Ni-B-P, and e-f) Ni-B-P-2 samples recorded at 1.6 V vs. RHE.

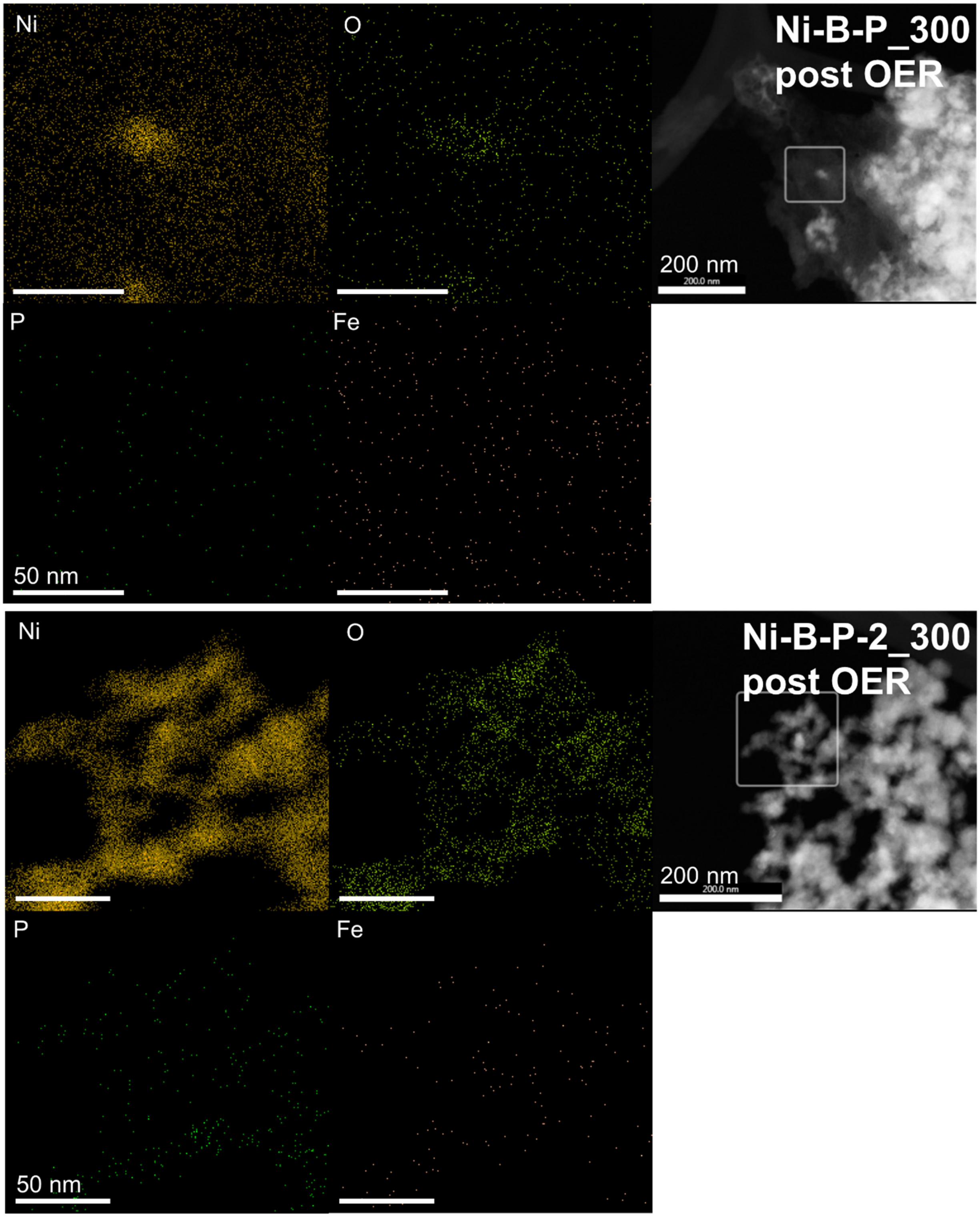

Figure S43. STEM-EDX elemental mapping of Ni-B-P_300 and Ni-B-P-2_300 after OER testing.

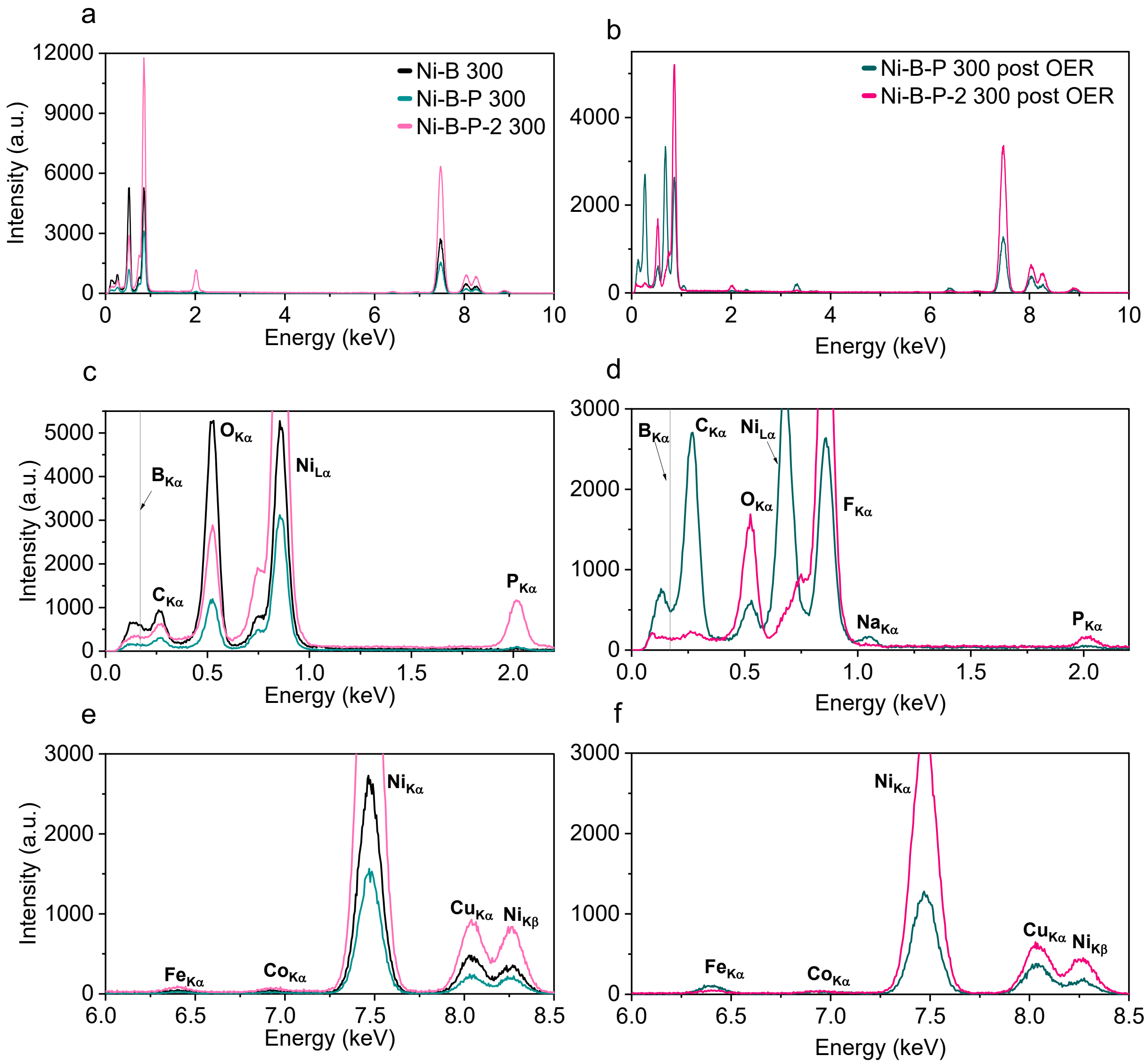

Figure S44. STEM-EDX spectra of Ni-B_300, Ni-B-P_300, and Ni-B-P-2_300 before OER testing a), with magnified sections in c) and e), and Ni-B-P_300 and Ni-B-P-2_300 after OER testing in b) with magnified sections in d) and f).

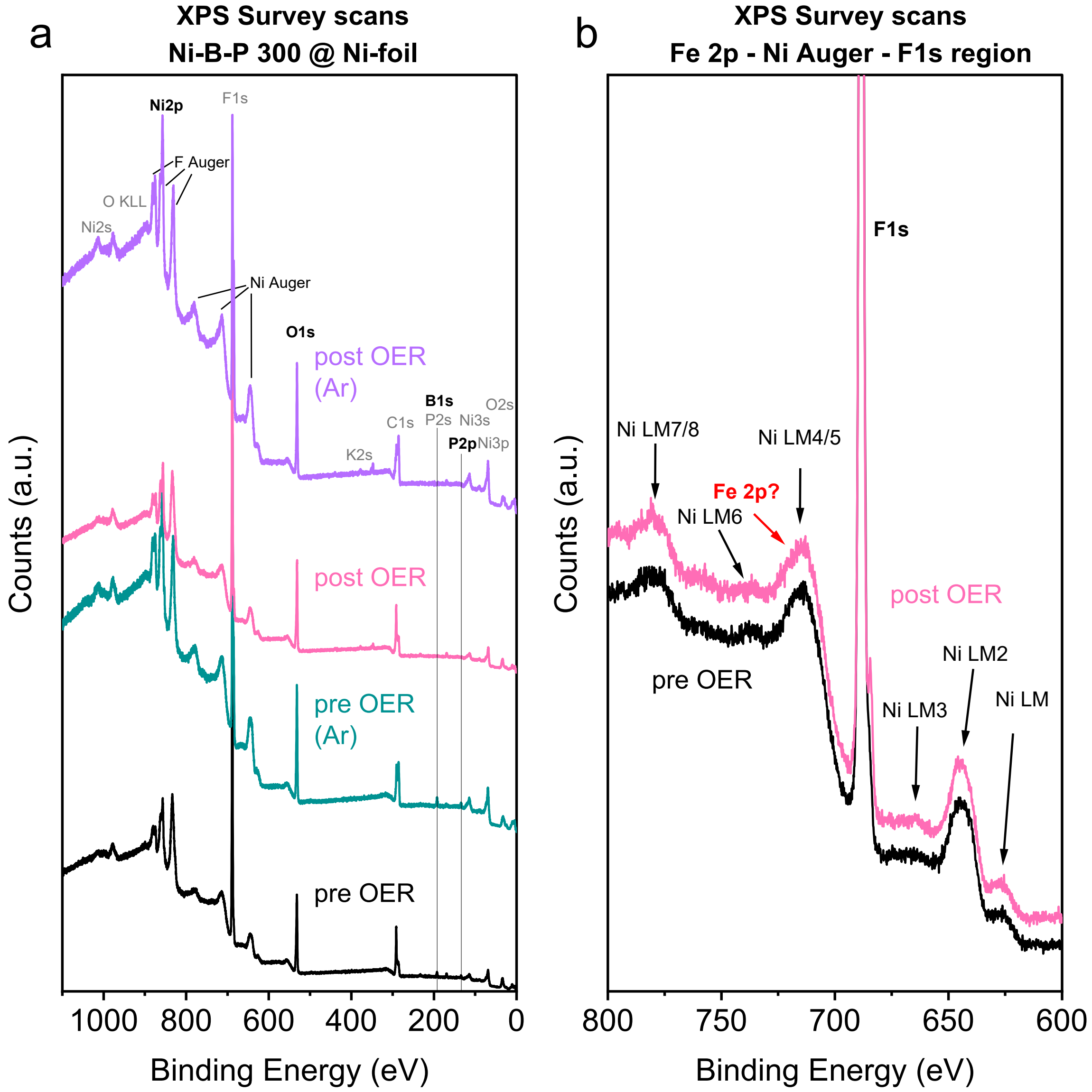

Figure S45. Survey spectra of Ni-B-P_300@Ni-foil pre and post OER measurement with and without Ar bombardment a) and detailed view of the 800 – 600 eV binding energy region b). The graphs are displayed in stacked form to improve visibility.

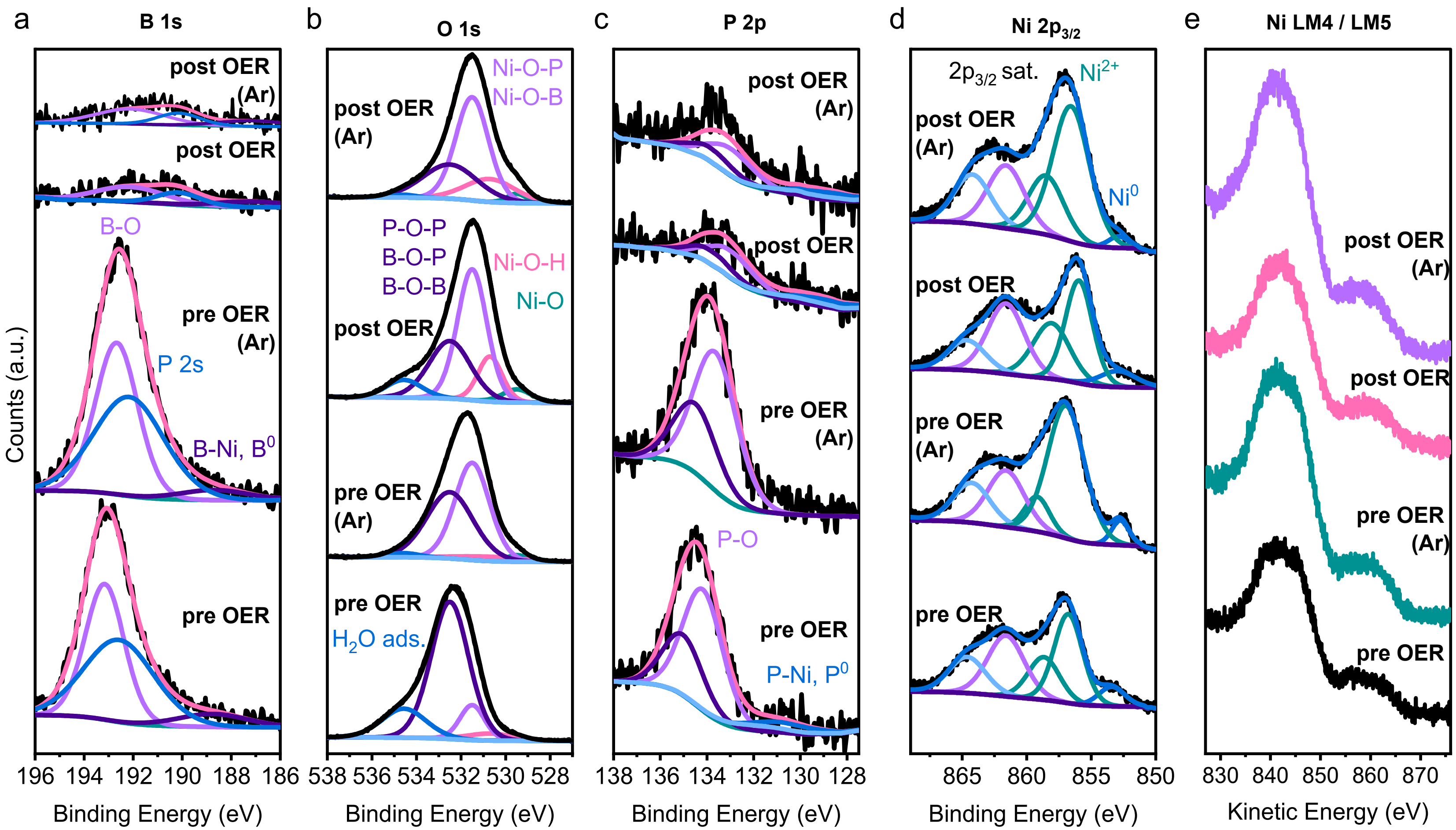

Figure S46. a) B 1s and b) O 1s c) P 2s, d) Ni $2p_{3/2}$, and e) Ni $L_3M_{45}M_{45}$ Auger HR-XPS spectra of Ni-B-P_300@Ni-foil pre and post OER measurement with and without Ar bombardment. The graphs are displayed in stacked form to improve visibility.

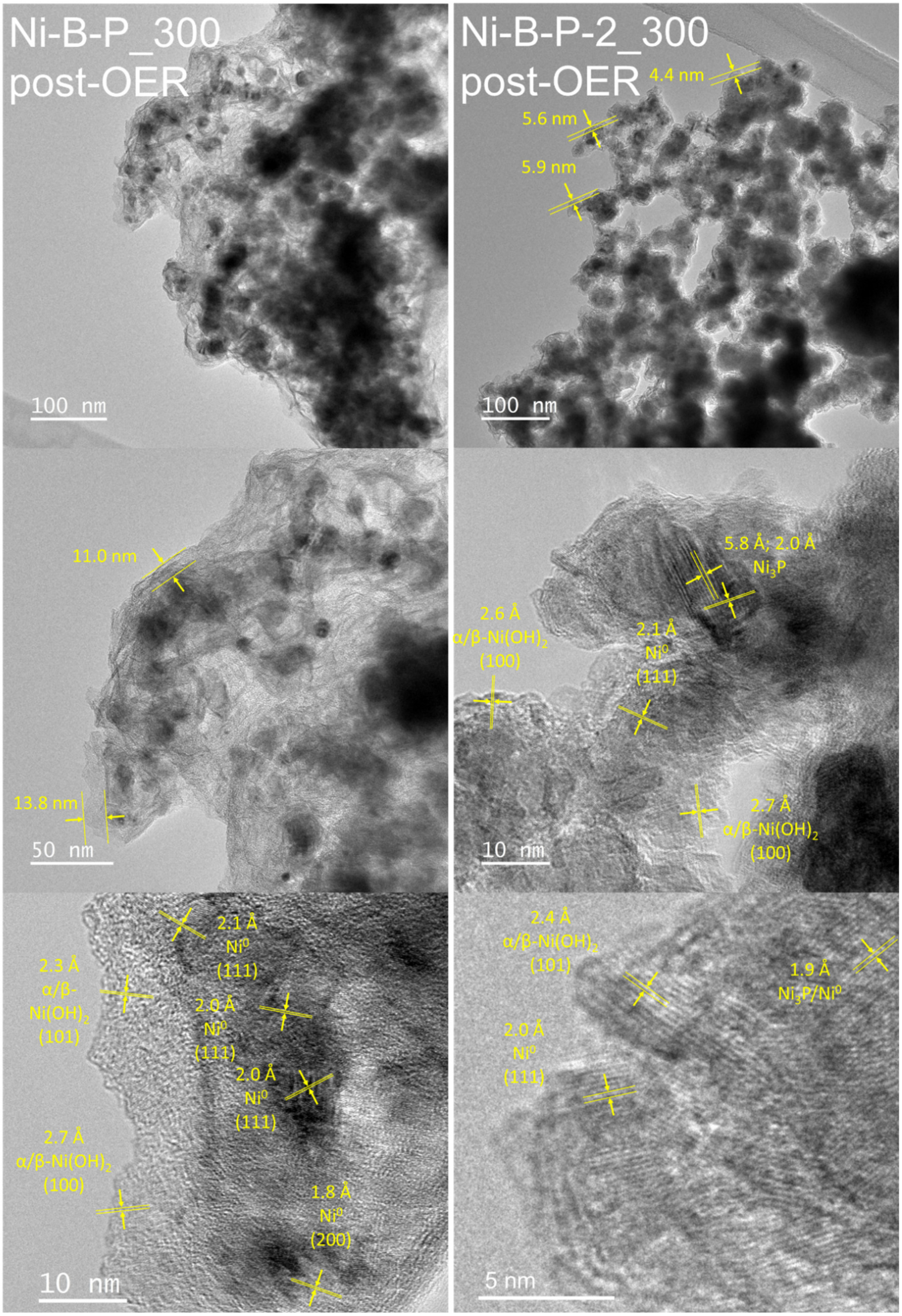

Figure S47. Bright-field TEM images of Ni-B-P_300 and Ni-B-P-2_300 after OER testing.

3. Tables S1 to S34:

Table S1. Weight change in wt% of Ni-B, Ni-B-P, Ni-B-P-2 as-made samples in selected intervals determined by TGA in N2 atmosphere.

Sample		35 – 200 °C	200 – 400 °C	400 – 600 °C
Ni-B	AM	−8.8	−5.3	−9.5
Ni-B-P	AM	−6.5	−2.0	−2.1
Ni-B-P-2	AM	−3.6	−1.2	0.4

Table S2. XRF bulk elemental composition in wt% and at%.

Sample		P [wt%]	Ni [wt%]	P [at%]	Ni [at%]
Ni-B	AM	0.0	100.0	0.0	100.0
Ni-B-P	AM	1.9	98.1	3.5	96.5
Ni-B-P-2	AM	4.5	95.5	8.2	91.8

Table S3. SEM-EDX bulk elemental composition in wt% and at%.

Sample		O [wt%]	Na [wt%]	P [wt%]	Ni [wt%]	O [at%]	Na [at%]	P [at%]	Ni [at%]
Ni-B	AM	34.6	0.5	0.0	64.9	65.7	0.7	0.0	33.6
Ni-B-P	AM	16.1	0.1	2.4	81.5	40.6	0.2	3.1	56.1
Ni-B-P-2	AM	4.9	0.0	6.0	89	15.2	0.0	9.6	75.2

Table S4. ICP-OES bulk elemental composition in wt% and at%.

Sample		B [wt%]	P [wt%]	Ni [wt%]	B [at%]	P [at%]	Ni [at%]
Ni-B	AM	7.6	0.0	92.4	30.9	0.0	69.1
	150	7.6	0.0	92.4	31.0	0.0	69.0
	200	7.6	0.0	92.4	30.9	0.0	69.1
	250	7.6	0.0	92.4	30.8	0.0	69.2
	300	7.5	0.0	92.5	30.6	0.0	69.4
	350	7.6	0.0	92.4	30.7	0.0	69.3
Ni-B-P	AM	5.5	2.2	92.3	23.5	3.3	73.2
	150	5.5	2.2	92.3	23.6	3.3	73.1
	200	5.5	2.2	92.3	23.6	3.4	73.0
	250	5.5	2.2	92.3	23.6	3.3	73.1
	300	5.5	2.2	92.2	23.8	3.3	72.9
	350	5.5	2.2	92.2	23.7	3.3	72.9
Ni-B-P-2	AM	3.6	5.4	91.0	16.1	8.5	75.4
	150	3.6	5.4	90.9	16.3	8.5	75.2
	200	3.6	5.4	91.0	16.2	8.5	75.4
	250	3.6	5.4	91.0	16.2	8.5	75.3
	300	3.6	5.8	90.6	16.1	9.1	74.8
	350	3.6	5.4	91.0	16.2	8.5	75.3

Table S5. Surface area and pore characterization with nitrogen physisorption (-196 °C) for Ni-B, Ni-B-P, and Ni-B-P-2 samples annealed at the indicated temperatures using the adsorption branch of the isotherm.

Sample		BET SSA [m^2 g^{-1}]	NLDFT SSA [m^2 g^{-1}]	NLDFT pore size [nm]	NLDFT V_p [cm^3 g^{-1}]	V_t (d < 80 nm) [cm^3 g^{-1}]
Ni-B	**AM**	93	89	6.6	0.13	0.13
	**150**	135	117	5.9	0.16	0.17
	**200**	97	90	6.1	0.13	0.13
	**250**	116	101	6.1	0.15	0.16
	**300**	134	121	6.1	0.20	0.19
	**350**	168	142	6.1	0.22	0.22
Ni-B-P	**AM**	48	50	10.5	0.38	0.19
	**150**	88	81	6.1	0.47	0.24
	**200**	60	59	7.0	0.36	0.2
	**250**	65	63	7.0	0.39	0.21
	**300**	50	52	9.1	0.35	0.19
	**350**	96	86	6.1	0.41	0.24
Ni-B-P-2	**AM**	26	26	29.4	0.28	0.09
	**150**	35	34	7.0	0.24	0.11
	**200**	31	31	8.1 / 29.4	0.27	0.11
	**250**	25	25	8.1 / 29.4	0.24	0.09
	**300**	23	25	8.1 / 29.4	0.26	0.09
	**350**	29	29	8.1 / 29.4	0.25	0.10

Table S6. N_2 physisorption (-196 °C) results of Ni-B-P-2 samples annealed at the indicated temperatures obtained using the NLDFT equilibrium branch.

Sample		$NLDFT_{equ}$ SSA [m^2 g^{-1}]	$NLDFT_{equ}$ pore size [nm]	$NLDFT_{equ}$ V_p [cm^3 g^{-1}]
Ni-B-P-2	AM	22	3.8	0.12
	150	31	4.9	0.14
	200	30	3.8	0.14
	250	23	3.8	0.11
	300	24	3.8	0.12
	350	26	4.9	0.12

Table S7. Surface composition in wt% and at% determined by XPS survey spectra.

Sample		B 1s [wt%]	O 1s [wt%]	P 2p [wt%]	Ni 2p [wt%]	B 1s [at%]	O 1s [at%]	P 2p [at%]	Ni 2p [at%]
Ni-B	300	6.4	33.6	0.0	59.9	16.0	56.5	0.0	27.5
Ni-B-P	AM	8.1	36.5	4.3	51.1	18.6	56.4	3.4	21.5
	150	9.2	36.0	4.4	50.4	20.8	54.9	3.4	20.9
	200	10.3	37.4	4.2	48.0	22.5	55.0	3.2	19.3
	250	13.1	38.2	4.2	44.5	26.9	53.2	3.0	16.9
	300	15.3	38.9	3.6	42.2	30.2	52.0	2.5	15.4
	350	17.1	41.3	3.9	37.7	32.1	52.3	2.6	13.0
Ni-B-P-2	300	21.0	40.0	8.0	31.0	37.2	47.8	4.9	10.1

Table S8. Surface composition excluding O 1s in wt% and at% determined by XPS survey spectra.

Sample		B 1s [wt%]	P 2p [wt%]	Ni 2p [wt%]	B 1s [at%]	P 2p [at%]	Ni 2p [at%]
Ni-B	300	9.7	0.0	90.3	36.8	0.0	63.2
Ni-B-P	AM	12.8	6.8	80.4	42.7	7.9	49.4
	150	14.4	6.8	78.8	46.0	7.6	46.3
	200	16.5	6.8	76.7	50.0	7.2	42.8
	250	21.2	6.8	72.0	57.5	6.5	36.1
	300	25.0	5.9	69.1	62.9	5.1	32.0
	350	29.1	6.7	64.2	67.3	5.4	27.3
Ni-B-P-2	300	35.1	13.3	51.6	71.3	9.4	19.3

Table S9. Peak position (E_{BE} in eV) of the species identified in the deconvoluted B 1s HR-XPS spectra.

Sample		Ni−B, B^0	B−O
Ni-B	300	-	191.9
Ni-B-P	AM	187.8	192.0
	150	187.7	192.0
	200	187.8	192.1
	250	188.0	192.4
	300	187.6	192.7
	350	187.5	192.6
Ni-B-P-2	300	187.5	192.8

Table S10. Relative abundance in at% of the species assigned to the deconvoluted B 1s HR-XPS spectra.

Sample		Ni−B, B^0	B−O
Ni-B	300	-	100.0
Ni-B-P	AM	13.0	87.0
	150	9.3	90.7
	200	6.3	93.7
	250	4.2	95.8
	300	3.1	96.9
	350	2.4	97.6
Ni-B-P-2	300	15.8	84.2

Table S11. Peak position (E_{BE} in eV) of the species identified the deconvoluted O 1s HR-XPS spectra.

Sample		Ni−O	Ni−OH	Ni−O−B Ni−O−P	O−B O−P
Ni-B	300	529.5	530.7	531.5	532.5
Ni-B-P	AM	529.5	530.7	531.5	532.5
	150	529.5	530.7	531.5	532.5
	200	529.5	530.7	531.5	532.5
	250	529.5	530.7	531.5	532.5
	300	529.5	530.7	531.5	532.5
	350	529.5	530.7	531.5	532.5
Ni-B-P-2	300	529.5	530.7	531.5	532.5

Table S12. Relative abundance in at% of the species assigned to the deconvoluted O 1s HR-XPS spectra.

Sample		Ni−O	Ni−OH	Ni−O−B<br>Ni−O−P	O−B<br>O−P
**Ni-B**	**300**	16.4	35.3	33.6	14.8
**Ni-B-P**	**AM**	2.6	28.9	47.2	21.3
	**150**	2.7	29.8	45.4	22.1
	**200**	2.3	20.8	47.2	29.7
	**250**	1.8	9.3	48.9	40.0
	**300**	0.5	2.7	34.8	62.0
	**350**	1.2	0.9	43.3	54.6
**Ni-B-P-2**	**300**	0.9	0.1	22.4	76.6

Table S13. Peak position binding energies (in eV) of the species identified in deconvoluted P 2p HR-XPS spectra.

Sample		P−Ni, P^0	P−O
**Ni-B**	**300**	-	-
**Ni-B-P**	**AM**	129.5	132.9
	**150**	129.4	132.9
	**200**	129.6	133.1
	**250**	129.6	133.3
	**300**	129.8	133.7
	**350**	129.9	133.6
**Ni-B-P-2**	**300**	129.7	133.6

Table S14. Relative abundance in at% of the species assigned to the deconvoluted P 2p HR-XPS spectra.

Sample		P−Ni, P^0	P−O
**Ni-B**	**300**	-	-
**Ni-B-P**	**AM**	8.5	91.5
	**150**	8.6	91.4
	**200**	8.4	91.6
	**250**	8.2	91.8
	**300**	10.5	89.5
	**350**	11.4	88.7
**Ni-B-P-2**	**300**	58.2	41.8

Table S15. Peak position binding energies (in eV) of the species identified in the Ni $2p_{3/2}$ HR-XPS spectra.

Sample		Ni–P, Ni^0	Ni^{2+}
Ni-B	300		855.3
Ni-B-P	AM	852.4	855.7
	150	852.4	855.8
	200	852.4	855.8
	250	852.5	856.0
	300	852.8	856.5
	350	852.6	856.3
Ni-B-P-2	300	852.7	856.5

Table S16. Relative abundance in at% of the species assigned to the deconvoluted Ni $2p_{3/2}$ HR-XPS spectra.

Sample		Ni–P, Ni^0	Ni^{2+}
Ni-B	300	-	100
Ni-B-P	AM	64.1	35.9
	150	58.0	42.0
	200	66.9	33.1
	250	61.2	38.8
	300	75.4	24.7
	350	68.6	31.4
Ni-B-P-2	300	92.0	8.0

Table S17. LEIS elemental abundance at the surface layer in at%.

Sample		Ni	O	P	B	Na
Ni-B	300	52	36	0	<1	12
Ni-B-P	AM	51	27	11	11	<1
	150	64	29	7	<1	<1
	200	52	37	11	<1	<1
	250	60	33	7	<1	<1
	300	59	36	5	<1	<1
	350	64	32	4	<1	<1
Ni-B-P-2	300	63	20	17	<1	<1

Table S18. Evolution of the electrical charge (in millicoulomb, mC) consumed by the precatalytic oxidation A_2 in the forward CV scan and the reduction $C_2 + C_3$ in the reverse scan with increasing CV cycle number for the Ni-B-P samples.

Ni-B-P Cycle Nr.	AM [mC]		150 [mC]		200 [mC]		250 [mC]		300 [mC]		350 [mC]	
	A_2	C_2+C_3	A_2	C_2+C_3	A_2	C_2+C_3	A_2	C_2+C_3	A_2	C_2+C_3	A_2	C_2+C_3
**2**	6.4	-3.2	5.1	-2.8	6.3	-3.6	5.6	-3.2	5.1	-3.0	3.7	-2.3
**3**	6.6	-3.4	5.9	-3.3	6.8	-3.9	6.7	-3.8	6.1	-3.6	4.8	-2.7
**4**	6.9	-3.5	6.5	-3.6	7.1	-4.1	7.2	-4.0	6.6	-3.9	5.3	-3.1
**5**	7.0	-3.6	6.6	-3.8	7.2	-4.2	7.5	-4.1	6.8	-4.1	5.8	-3.2
**6**	7.0	-3.7	6.9	-3.9	7.4	-4.2	7.6	-4.2	7.0	-4.3	6.0	-3.3
**7**	7.1	-3.7	7.1	-4.0	7.3	-4.2	7.7	-4.3	7.2	-4.4	6.2	-3.4
**8**	7.1	-3.7	7.2	-4.0	7.3	-4.2	7.8	-4.3	7.3	-4.5	6.3	-3.5
**9**	7.1	-3.7	7.2	-4.1	7.3	-4.2	7.8	-4.4	7.4	-4.6	6.4	-3.5
**10**	7.1	-3.7	7.3	-4.1	7.3	-4.2	7.9	-4.4	7.4	-4.6	6.4	-3.5
**20**	7.1	-3.7	7.4	-4.1	7.2	-4.1	8.0	-4.4	7.5	-4.6	6.5	-3.5
**30**	7.0	-3.6	7.4	-4.0	7.0	-3.9	7.9	-4.2	7.4	-4.5	6.3	-3.3
**40**	6.8	-3.6	7.3	-4.0	6.8	-3.7	7.9	-4.1	7.4	-4.5	6.0	-3.2
**50**	6.6	-3.5	7.2	-3.9	6.5	-3.6	7.8	-4.1	7.3	-4.4	5.7	-3.1
**60**	6.4	-3.5	7.2	-3.9	6.2	-3.5	7.7	-4.0	7.3	-4.4	5.4	-3.0
**70**	6.2	-3.5	7.2	-3.9	5.9	-3.4	7.6	-4.0	7.2	-4.3	5.0	-2.9
**80**	5.9	-3.4	7.2	-3.9	5.6	-3.3	7.5	-3.9	7.2	-4.3	4.7	-2.9
**90**	5.6	-3.4	7.2	-3.8	5.3	-3.3	7.4	-3.9	7.2	-4.3	4.4	-2.8
**100**	5.4	-3.4	7.1	-3.8	5.0	-3.2	7.2	-3.9	7.2	-4.3	4.1	-2.8

Table S19. Evolution of the electrical charge (in millicoulomb, mC) consumed by the precatalytic oxidation A_2 in the forward CV scan and the reduction $C_2 + C_3$ in the reverse scan with increasing CV cycle number for the Ni-B-P-2 samples.

Ni-B-P-2 Cycle Nr.	AM [mC]		150 [mC]		200 [mC]		250 [mC]		300 [mC]		350 [mC]	
	A_2	C_2+C_3	A_2	C_2+C_3	A_2	C_2+C_3	A_2	C_2+C_3	A_2	C_2+C_3	A_2	C_2+C_3
**2**	4.8	-3.6	3.0	-2.2	3.2	-2.4	3.1	-2.3	3.5	-2.5	3.3	-2.5
**3**	5.0	-3.6	3.3	-2.3	3.4	-2.7	3.3	-2.5	3.6	-2.7	3.5	-2.6
**4**	5.2	-3.5	3.5	-2.3	3.6	-2.9	3.3	-2.6	3.8	-2.8	3.7	-2.6
**5**	5.4	-3.5	3.7	-2.3	3.8	-2.9	3.5	-2.7	3.9	-2.9	3.7	-2.7
**6**	5.5	-3.4	3.8	-2.3	3.9	-2.9	3.6	-2.8	4.0	-2.9	3.8	-2.7
**7**	5.7	-3.3	3.9	-2.3	4.1	-2.9	3.7	-2.9	4.0	-3.0	3.9	-2.7
**8**	5.8	-3.3	4.1	-2.3	4.2	-2.9	3.8	-2.9	4.1	-3.0	3.9	-2.8
**9**	5.9	-3.2	4.1	-2.3	4.3	-2.9	3.9	-2.9	4.2	-3.0	4.0	-2.8
**10**	6.0	-3.2	4.2	-2.3	4.4	-3.0	3.9	-3.0	4.2	-3.0	4.0	-2.8
**20**	6.8	-3.1	4.8	-2.4	5.1	-3.0	4.4	-3.1	4.6	-3.2	4.5	-2.9
**30**	7.2	-3.2	5.1	-2.5	5.5	-3.0	4.7	-3.1	4.9	-3.3	4.7	-3.0
**40**	7.4	-3.3	5.3	-2.6	5.7	-3.1	4.9	-3.1	5.0	-3.3	4.8	-3.1
**50**	7.4	-3.4	5.3	-2.7	5.8	-3.1	5.0	-3.1	5.1	-3.3	5.0	-3.1
**60**	7.3	-3.4	5.4	-2.8	5.8	-3.2	5.1	-3.1	5.1	-3.3	5.0	-3.2
**70**	7.3	-3.5	5.4	-2.8	5.9	-3.2	5.2	-3.1	5.1	-3.3	5.1	-3.3
**80**	7.2	-3.5	5.4	-2.9	5.9	-3.2	5.3	-3.2	5.2	-3.3	5.2	-3.3
**90**	7.2	-3.6	5.5	-3.0	5.8	-3.3	5.4	-3.1	5.2	-3.3	5.3	-3.3
**100**	7.1	-3.6	5.6	-3.0	5.9	-3.3	5.4	-3.2	5.2	-3.3	5.4	-3.4

Table S20. Electrocatalytic OER performance of Ni-B, Ni-B-P, and Ni-B-P-2 samples, and RuO_2 commercial reference in 1M KOH and 25 °C, including overpotential (η) to achieve 10 mA cm^{-2}, current density (i) at 1.65 V vs. RHE, Tafel slope in the kinetic region, and turnover frequency at 1.65 V vs. RHE.

Sample		η for 10 mA cm^{-2} [mV]	i at 1.65 V vs. RHE [mA cm^{-2}]	Tafel slope [mV dec^{-1}]	TOF at 1.65 V vs. RHE [s^{-1}]
Ni-B	AM	385 (±8)	18 (±3)	91 (±4)	0.04 (±0.01)
	300	375 (±4)	22 (±2)	81 (±2)	0.05 (±0.00)
Ni-B-P	AM	287 (±1)	137 (±5)	54 (±1)	0.20 (±0.01)
	150	286 (±1)	146 (±18)	56 (±1)	0.21 (±0.03)
	200	288 (±2)	117 (±1)	55 (±1)	0.17 (±0.00)
	250	284 (±1)	195 (±5)	48 (±1)	0.29 (±0.01)
	300	281 (±1)	500 (±56)	44 (±1)	0.79 (±0.09)
	350	300 (±1)	77 (±10)	57 (±1)	0.12 (±0.02)
Ni-B-P-2	AM	291 (±1)	235 (±28)	48 (±1)	0.30 (±0.04)
	150	290 (±0)	330 (±3)	45 (±0)	0.45 (±0.00)
	200	291 (±2)	300 (±0)	44 (±0)	0.38 (±0.00)
	250	292 (±1)	259 (±0)	46 (±0)	0.34 (±0.00)
	300	289 (±2)	215 (±29)	48 (±0)	0.33 (±0.04)
	350	292 (±3)	171 (±28)	49 (±3)	0.24 (±0.04)
RuO_2	Ref.	242 (±0)	423 (±16)	50 (±0)	1.19 (±0.04)

Table S21. Electrochemical double layer capacitance, electrochemical active surface area, and roughness factor of Ni-B, Ni-B-P, and Ni-B-P-2 samples, and commercial RuO_2 reference material reflecting the mean values and standard deviations obtained from triplicates.

Sample		C_{dl}·10^{-6} (µF)	ECSA (cm^2)	Rf
Ni-B	AM	27 (±16)	0.7 (±0.4)	3.4 (±2.0)
	300	33 (±18)	0.8 (±0.5)	4.2 (±2.3)
Ni-B-P	AM	30 (±18)	0.7 (±0.4)	3.8 (±2.3)
	150	31 (±20)	0.8 (±0.5)	3.9 (±2.5)
	200	31 (±17)	0.8 (±0.4)	3.9 (±2.2)
	250	30 (±16)	0.7 (±0.4)	3.8 (±2.0)
	300	31 (±16)	0.8 (±0.4)	4.0 (±2.0)
	350	30 (±12)	0.8 (±0.3)	3.9 (±1.6)
Ni-B-P-2	AM	27 (±14)	0.7 (±0.4)	3.4 (±1.8)
	150	35 (±16)	0.9 (±0.4)	4.4 (±2.1)
	200	37 (±19)	0.9 (±0.5)	4.7 (±2.4)
	250	34 (±14)	0.8 (±0.3)	4.3 (±1.7)
	300	34 (±16)	0.9 (±0.4)	4.4 (±2.0)
	350	32 (±12)	0.8 (±0.3)	4.1 (±1.6)
RuO_2	Ref.	667 (±133)	16.7 (±3.3)	84.9 (±16.9)

Table S22. Nyquist plot evaluation for Ni-B-P_300 and Ni-B-P-2_300 in operando EIS measurements performed at 1.40, 1.45, 1.50, 1.55, and 1.60 V vs. RHE using the R(QR)(QR) equivalent circuit.

Sample	E appl. vs. RHE	R_u [Ω]	R_1 [Ω]	Q_1 [µS s^N]	N	R_{ct} [Ω]	Q_2 [µS s^N]	N	χ^2
Ni-B-P 300	**1.40 V**	4.3	1.1	7579	0.43	3756	5857	0.48	0.010
	**1.45 V**	4.4	0.9	15113	0.42	415	5957	0.47	0.008
	**1.50 V**	4.3	1.0	8698	0.42	17.1	4653	0.95	0.007
	**1.55 V**	4.5	0.7	541	0.69	3.2	4379	0.93	0.005
	**1.60 V**	4.6	0.7	91.5	0.84	1.6	5709	0.86	0.005
Ni-B-P-2 300	**1.40 V**	4.3	3.3	31.7	0.86	4591	4512	0.96	0.007
	**1.45 V**	4.3	2.6	46.9	0.84	716	4676	0.34	0.009
	**1.50 V**	4.3	2.3	35.8	0.86	27.5	3881	0.95	0.009
	**1.55 V**	4.4	2.5	24.0	0.89	4.7	3841	0.92	0.005
	**1.60 V**	4.5	2.4	21.7	0.90	2.1	4345	0.89	0.006

Table S23. Bode plot derived admittance (Y), total magnitude |Z|, phase angles (Θ) and peak frequencies (ω_{pf}) for the Ni-B-P 300 and Ni-B-P-2 300 operando EIS measurements performed between 1.1 - 1.6 V vs. RHE.

Sample	E appl. vs. RHE	Y [mS]	|Z| [Ω]	Θ_1 [°]	$\omega_{pf,1}$ [Hz]	Θ_2 [°]	$\omega_{pf,2}$ [Hz]
Ni-B-P 300	**1.10 V**	-	14752	76.6	184	-	-
	**1.20 V**	-	10964	74.7	257	-	-
	**1.30 V**	-	6735	71.3	364	-	-
	**1.35 V**	-	232	8.2	14500	69.2	0.3
	**1.40 V**	-	270	2.9	25700	81.1	0.3
	**1.45 V**	-	220	2.3	25700	73.0	0.6
	**1.50 V**	71.5	22.0	2.3	24800	35.7	4.7
	**1.55 V**	13.3	8.4	2.6	25200	12.3	18.1
	**1.60 V**	10.3	6.8	2.8	25200	6.0	40.6
Ni-B-P-2 300	**1.10 V**	-	11905	74.0	496	-	-
	**1.20 V**	-	11367	73.5	549	-	-
	**1.30 V**	-	8904	72.7	633	-	-
	**1.35 V**	-	319	48.5	2485	-	-
	**1.40 V**	-	349	13.2	10100	80.0	0.1
	**1.45 V**	-	303	10.8	11300	75.1	0.5
	**1.50 V**	117	33.6	10.3	11100	39.6	4.0
	**1.55 V**	17.2	11.5	9.6	12700	13.2	16.1
	**1.60 V**	12.5	9.0	10.3	12500	6.4	40.2

Table S24. Nyquist plots fitting results obtained using the R(QR)(QR) equivalent circuit for the EIS measurements conducted at 1.6 V vs. RHE.

Sample		R_u [Ω]	R_1 [Ω]	Q_1 [µS s^N]	N	R_{ct} [Ω]	Q_2 [µS s^N]	N	χ^2
Ni-B	AM	4.4	26.9	31	0.86	11.6	707	0.75	0.009
	300	4.4	17.5	30	0.87	12.1	637	0.74	0.007
Ni-B-P	AM	4.5	3.8	46	0.85	2.6	7270	0.79	0.007
	150	4.4	5.6	46	0.84	2.8	5474	0.86	0.010
	200	4.4	5.6	46	0.84	2.8	5474	0.86	0.010
	250	4.4	3.1	42	0.84	2.2	6029	0.87	0.007
	300	4.6	0.6	276	0.76	1.7	5058	0.88	0.007
	350	4.5	8.5	28	0.87	4.1	6076	0.81	0.011
Ni-B-P-2	AM	4.5	2.0	57	0.84	2.2	6128	0.87	0.010
	150	4.5	1.2	79	0.83	2.0	5999	0.85	0.007
	200	4.5	1.2	23	0.92	2.0	5394	0.88	0.007
	250	4.5	1.4	56	0.86	2.0	4477	0.88	0.008
	300	4.5	2.2	18	0.91	2.1	4112	0.90	0.005
	350	4.5	2.7	32	0.88	2.2	4775	0.90	0.006

Table S25. Nyquist plots fitting parameters obtained for the RuO_2 commercial reference EIS measurement conducted at 1.6 V vs. RHE using the R(QR) equivalent circuit.

Sample		R_u [Ω]	R_{ct} [Ω]	Q [S s^N]	N
RuO_2	Ref.	4.3	2.5	1970	0.89

Table S26. Bode plot derived admittance (Y), total magnitude |Z|, phase angles (Θ) and peak frequencies (ω_{pf}) for EIS measurements conducted at 1.6 V vs. RHE for Ni-B, Ni-B-P and Ni-B-P-2 samples.

Sample		Y [mS]	|Z| [Ω]	Θ_1 [°]	$\omega_{pf,1}$ [Hz]	Θ_2 [°]	$\omega_{pf,2}$ [Hz]
Ni-B	AM	106.4	42.6	41.7	1900	-	-
	300	78.1	33.9	37.0	2400	-	-
Ni-B-P	AM	16.0	11.9	14.9	6300	6.0	15.8
	150	14.0	11.0	13.4	6300	6.1	25.1
	200	16.3	12.6	17.7	5200	5.8	19.9
	250	12.9	9.8	11.2	10000	6.1	25.1
	300	9.8	6.9	2.3	32000	6.7	39.8
	350	21.8	17.2	23.6	4200	6.8	12.6
Ni-B-P-2	AM	11.7	8.8	7.6	10000	6.7	24.2
	150	11.0	7.9	5.1	15000	6.8	31.5
	200	11.1	7.9	5.6	18800	7.2	32.0
	250	11.2	7.9	6.0	13700	6.9	40.0
	300	12.8	8.9	9.7	14200	6.9	35.9
	350	14.4	10.6	13.7	9000	7.1	22.3
RuO_2	Ref.	17.1	7.0	10.9	70.3	-	-

Table S27. Ni-B-P 300@Ni-foil surface composition pre and post OER in at% determined by XPS survey spectra pre and post OER testing and with and without Ar sputtering.

Sample		B 1s [wt%]	O 1s [wt%]	P 2p [wt%]	Ni 2p [wt%]	B 1s [at%]	O 1s [at%]	P 2p [at%]	Ni 2p [at%]
Ni-B-P 300	pre OER	8.3	31.2	2.8	57.7	20.1	51.5	2.4	25.9
	pre OER (Ar)	8.3	23.6	2.7	65.4	22.4	42.8	2.5	32.3
	post OER	2.5	32.7	1.2	63.7	6.7	60.2	1.1	32.0
	post OER (Ar)	1.7	22.2	1.2	74.9	5.5	48.5	1.4	44.7

Table S28. Ni-B-P 300@Ni-foil Peak position (E_{BE} in eV) of the species identified in the deconvoluted B 1s HR-XPS spectra pre and post OER testing and with and without Ar sputtering.

Sample		Ni-B, B^0	B−O
Ni-B-P 300	pre OER	188.8	193.2
	pre OER (Ar)	188.7	192.7
	post OER	187.2	192.1
	post OER (Ar)	187.2	192.1

Table S29. Ni-B-P 300@Ni-foil relative abundance in at% of the species assigned to the deconvoluted B 1s HR-XPS spectra pre and post OER testing and with and without Ar sputtering.

Sample		Ni-B, B^0	B−O
Ni-B-P 300	pre OER	14.5	85.5
	pre OER (Ar)	9.8	90.2
	post OER	27.3	72.7
	post OER (Ar)	27.3	72.7

Table S30. Ni-B-P 300@Ni-foil peak position (E_{BE} in eV) of the species identified the deconvoluted O 1s HR-XPS spectra pre and post OER testing and with and without Ar sputtering.

Sample		Ni−O	Ni−OH	Ni−O−B Ni−O−P	P−O B−O	H_2O ads.
Ni-B-P 300	pre OER	529.5	530.7	531.5	532.5	534.6
	pre OER (Ar)	529.5	530.7	531.5	532.5	534.6
	post OER	529.5	530.7	531.5	532.5	534.6
	post OER (Ar)	529.5	530.7	531.5	532.5	534.6

Table S31. Ni-B-P 300@Ni-foil relative abundance in at% of the species assigned to the deconvoluted O 1s HR-XPS spectra pre and post OER testing and with and without Ar sputtering.

Sample		Ni−O	Ni−OH	Ni−O−B Ni−O−P	O−P O−B	H_2O ads.
Ni-B-P 300	pre OER	0.6	4.2	11.3	67.3	16.6
	pre OER (Ar)	1.9	3.9	48.3	44.2	1.7
	post OER	4.5	13.1	46.1	28.7	7.6
	post OER (Ar)	2.7	17.2	51.0	28.2	1.0

Table S32. Ni-B-P 300@Ni-foil peak position binding energies (in eV) of the species identified in deconvoluted P 2p HR-XPS spectra pre and post OER testing and with and without Ar sputtering.

Sample		P-Ni, P^0	P−O
Ni-B-P 300	pre OER	131.0	134.2
	pre OER (Ar)	-	133.6
	post OER	129.2	133.0
	post OER (Ar)	129.2	133.0

Table S33. Ni-B-P 300@Ni-foil relative abundance in at% of the species assigned to the deconvoluted P 2p HR-XPS spectra pre and post OER testing and with and without Ar sputtering.

Sample		P-Ni, P^0	P−O
**Ni-B-P 300**	pre OER	12.1	87.9
	pre OER (Ar)	-	100.0
	post OER	28.5	71.6
	post OER (Ar)	28.5	71.6

Table S34. Ni-B-P 300@Ni-foil peak position binding energies (in eV) of the species identified in the Ni $2p_{3/2}$ HR-XPS spectra pre and post OER testing and with and without Ar sputtering.

Sample		Ni^0, Ni-P/B	Ni^{2+}
**Ni-B-P 300**	pre OER	853.4	856.8
	pre OER (Ar)	852.7	857.0
	post OER	852.9	856.0
	post OER (Ar)	852.9	856.6

Table S35. Ni-B-P 300@Ni-foil relative abundance in at% of the species assigned to the deconvoluted Ni $2p_{3/2}$ HR-XPS spectra pre and post OER testing and with and without Ar sputtering.

Sample		Ni^0, Ni-P/B	Ni^{2+}
**Ni-B-P 300**	pre OER	16.6	83.4
	pre OER (Ar)	8.1	91.9
	post OER	17.2	82.8
	post OER (Ar)	4.4	95.6

References:

(1) Miura, Y.; Kusano, H.; Nanba, T.; Matsumoto, S. X-Ray Photoelectron Spectroscopy of Sodium Borosilicate Glasses. *J. Non. Cryst. Solids* **2001**, *290* (1), 1–14. https://doi.org/10.1016/S0022-3093(01)00720-7.

(2) Legrand, J.; Taleb, A.; Gota, S.; Guittet, M. J.; Petit, C. Synthesis and XPS Characterization of Nickel Boride Nanoparticles. *Langmuir* **2002**, *18* (10), 4131–4137. https://doi.org/10.1021/la0117247.

(3) Pfeiffer, H.; Tancret, F.; Brousse, T. Synthesis, Characterization and Thermal Stability of Ni_3P Coatings on Nickel. *Mater. Chem. Phys.* **2005**, *92* (2–3), 534–539. https://doi.org/10.1016/j.matchemphys.2005.01.055.

(4) Glavee, G. N.; Klabunde, K. J.; Sorensen, C. M.; Hadjipanayis, G. C. Borohydride Reduction of Nickel and Copper Ions in Aqueous and Nonaqueous Media. Controllable Chemistry Leading to Nanoscale Metal and Metal Boride Particles. *Langmuir* **1994**, *10* (12), 4726–4730. https://doi.org/10.1021/la00024a055.

(5) Shimizu, M.; Tsushima, Y.; Arai, S. Electrochemical Na-Insertion/Extraction Property of Ni-Coated Black Phosphorus Prepared by an Electroless Deposition Method. *ACS Omega* **2017**, *2* (8), 4306–4315. https://doi.org/10.1021/acsomega.7b00950.

(6) Masa, J.; Sinev, I.; Mistry, H.; Ventosa, E.; de la Mata, M.; Arbiol, J.; Muhler, M.; Roldan Cuenya, B.; Schuhmann, W. Ultrathin High Surface Area Nickel Boride (NixB) Nanosheets as Highly Efficient Electrocatalyst for Oxygen Evolution. *Adv. Energy Mater.* **2017**, *7* (17), 1–8. https://doi.org/10.1002/aenm.201700381.

(7) Biesinger, M. C.; Lau, L. W. M.; Gerson, A. R.; Smart, R. S. C. The Role of the Auger Parameter in XPS Studies of Nickel Metal, Halides and Oxides. *Phys. Chem. Chem. Phys.* **2012**, *14* (7), 2434–2442. https://doi.org/10.1039/c2cp22419d.

(8) Bode, H.; Dehmelt, K.; Witte, J. To the Knowledge of the Nickel Hydroxide Electrode-I. Over the Nickel (II)-Hydroxide Hydrate. *Electrochim. Acta* **1966**, *11* (September 1965), 1079–1087. https://doi.org/https://doi.org/10.1016/0013-4686(66)80045-2.
